# TECPR1 conjugates LC3 to damaged endomembranes upon detection of sphingomyelin exposure

**DOI:** 10.15252/embj.2022113012

**Published:** 2023-07-06

**Authors:** Keith B Boyle, Cara J Ellison, Paul R Elliott, Martina Schuschnig, Krista Grimes, Marc S Dionne, Chihiro Sasakawa, Sean Munro, Sascha Martens, Felix Randow

**Affiliations:** ^1^ Division of Protein and Nucleic Acid Chemistry MRC Laboratory of Molecular Biology Cambridge UK; ^2^ Max Perutz Labs, Vienna BioCenter (VBC) University of Vienna Vienna Austria; ^3^ MRC Centre for Molecular Bacteriology and Infection Imperial College London London UK; ^4^ Medical Mycology Research Center Chiba University Chiba Japan; ^5^ Nippon Institute for Biological Science Ome Japan; ^6^ Center for Molecular Biology, Department of Biochemistry and Cell Biology University of Vienna Vienna Austria; ^7^ Department of Medicine, Addenbrooke's Hospital University of Cambridge Cambridge UK; ^8^ Present address: Department of Biochemistry University of Oxford Oxford UK

**Keywords:** ATG5‐ATG12 E3 ligase, autophagy, DysF, membrane damage, sphingomyelin, Autophagy & Cell Death, Membranes & Trafficking

## Abstract

Invasive bacteria enter the cytosol of host cells through initial uptake into bacteria‐containing vacuoles (BCVs) and subsequent rupture of the BCV membrane, thereby exposing to the cytosol intraluminal, otherwise shielded danger signals such as glycans and sphingomyelin. The detection of glycans by galectin‐8 triggers anti‐bacterial autophagy, but how cells sense and respond to cytosolically exposed sphingomyelin remains unknown. Here, we identify TECPR1 (tectonin beta‐propeller repeat containing 1) as a receptor for cytosolically exposed sphingomyelin, which recruits ATG5 into an E3 ligase complex that mediates lipid conjugation of LC3 independently of ATG16L1. TECPR1 binds sphingomyelin through its N‐terminal DysF domain (N'DysF), a feature not shared by other mammalian DysF domains. Solving the crystal structure of N'DysF, we identified key residues required for the interaction, including a solvent‐exposed tryptophan (W154) essential for binding to sphingomyelin‐positive membranes and the conjugation of LC3 to lipids. Specificity of the ATG5/ATG12‐E3 ligase responsible for the conjugation of LC3 is therefore conferred by interchangeable receptor subunits, that is, the canonical ATG16L1 and the sphingomyelin‐specific TECPR1, in an arrangement reminiscent of certain multi‐subunit ubiquitin E3 ligases.

## Graphical abstract

TECPR1 detects cytosolically‐exposed sphingomyelin and recruits ATG5/ATG12‐E3 ligase to damaged membranes to mediate lipid conjugation of LC3 independently of ATG16L.

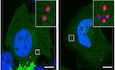

## Introduction

Membrane integrity is essential for cellular homeostasis. Both repair and disposal of damaged membranes rely on the immediate and sensitive detection of even minor membrane perturbations (Ammendolia *et al*, 2021; Zhen *et al*, 2021). While sensing damage to the plasma membrane is essential to avoid imminent cell death from cytosol leakage, damage to internal membranes is often less acute but nevertheless carefully monitored by cells as a tell‐tale sign of pathogen invasion (Randow *et al*, 2013; Andrews & Corrotte, 2018). The latter scenario includes membrane disruptions brought about when pathogens invade the cytosol from an endosomal or phagosomal compartment. During such breakage of compartment borders otherwise intraluminal molecules, collectively known as damage‐associated molecular patterns (DAMPs), become exposed to the cytosol for detection by dedicated cytosolic receptors (Gong *et al*, 2020). Intraluminal glycans are a well‐established class of DAMP that are sensed by cytosolic lectins, notably galectin‐8, which recruits the autophagy cargo receptor NDP52 to direct macroautophagy against the broken vesicle, while simultaneously entrapping the damage‐causing pathogen as by‐catch (Dupont *et al*, 2009; Thurston *et al*, 2009, 2012; Staring *et al*, 2017). Such “nonspecific” DAMP‐dependent detection of membrane damage via galectins enables cells to sense invasion by bacteria and viruses, as well as to detect sterile damage caused by, for example, the uptake of Tau aggregates, crystals or lysosomotropic agents such as LLOMe (Paz *et al*, 2010; Thurston *et al*, 2012; Staring *et al*, 2017; Falcon *et al*, 2018; Wang *et al*, 2020).

In addition to macroautophagy, cells deploy a variety of autophagy‐related processes, sometimes collectively referred to as noncanonical autophagy, when sensing infectious content in phagosomes and/or perturbations in vesicular homeostasis (Cadwell, 2016; Münz, 2021; Nieto‐Torres *et al*, 2021). Despite responding to different stimuli, both macro‐ and noncanonical autophagy rely on the activity of a trimeric E3 ligase, comprised of ATG5, ATG12 and ATG16L1, for the conjugation of LC3/GABARAP proteins to lipids in target membranes (Mizushima *et al*, 2011; Bento *et al*, 2015). Recruitment of ATG16L1 to the correct membrane results in productive LC3/GABARAP conjugation, while enforced recruitment to incorrect sites causes aberrant LC3/GABARAP conjugation to, for example, the plasma membrane (Fujita *et al*, 2008). During macroautophagy, recruitment of ATG16L1 occurs through WIPI proteins, thereby leading to the conjugation of LC3/GABARAP to specialised phagophore membranes (Dooley *et al*, 2018). By contrast, during noncanonical autophagy LC3/GABARAP proteins are conjugated to the limiting membrane of a variety of pre‐existing vesicles (Kageyama *et al*, 2011; Fischer *et al*, 2020; Nieto‐Torres *et al*, 2021; Ulferts *et al*, 2021; Hooper *et al*, 2022). For example, during LC3‐associated phagocytosis (LAP) LC3 is attached to pathogen‐containing phagosomes upon engagement of Toll‐like receptors (Martinez *et al*, 2015), while during LC3‐associated endocytosis (LANDO) LC3 is conjugated to endosomes loaded with amyloid‐beta protein (Heckmann *et al*, 2019). Recruitment of ATG16L1 during noncanonical autophagy relies, at least in some cases, on its C‐terminal WD40 repeats, present only in higher eukaryotes (Xu *et al*, 2019; Fischer *et al*, 2020; Ulferts *et al*, 2021; Hooper *et al*, 2022). However, besides the vacuolar ATPase and the transmembrane protein TMEM59 (Boada‐Romero *et al*, 2013), the identity of the ATG16L1‐recruitment signals remains largely unknown.

The enterobacterium *Salmonella enterica* serovar Typhimurium (*S*. Typhimurium) colonises a vacuolar compartment inside host cells, from where it sporadically escapes into the cytosol. By contrast, the related bacterium *Shigella flexneri* escapes from its vacuole with high efficacy and colonises the cytosol due to its ability to suppress cytosolic defence pathways (Fredlund & Enninga, 2014; Schaible & Haas, 2019). Both uptake of *S*. Typhimurium into and escape from the vacuole require the bacterial SpiI Type III Secretion System, a syringe‐like structure that transfers bacterial effector proteins into the host cytosol (Du *et al*, 2016). Vacuolar *S*. Typhimurium are targeted by a LAP‐like process in which vacuoles with impaired transmembrane ion gradients undergo structural changes in their vacuolar ATPase that trigger the recruitment of ATG16L1 and conjugation of LC3 (Kageyama *et al*, 2011; Xu *et al*, 2019; Ulferts *et al*, 2021; Hooper *et al*, 2022). The ability of the *S*. Typhimurium effector protein SopF to antagonise the pathway through ADP ribosylation of the vacuolar ATPase highlights its biological significance (Lau *et al*, 2019; Xu *et al*, 2019; Hooper *et al*, 2022).

We recently discovered that preceding the catastrophic membrane damage caused by the physical escape of bacteria from vacuoles, sphingomyelin, an asymmetrically distributed sphingolipid enriched in the luminal leaflet of vacuolar membranes, undergoes transbilayer movement to the cytosolic face (Ellison *et al*, 2020). Display of cytosolic sphingomyelin on stressed, but otherwise largely intact, membranes and exposure of glycans on fully ruptured membranes have revealed that the transition of bacteria from their vacuole into the host cytosol is a precisely choreographed multi‐step process. Early exposure of sphingomyelin to the cytosol could therefore provide an advanced warning signal of imminent bacterial entry independent of, but potentially synergizing with, glycan exposure. However, the detection of sphingomyelin in the host cytosol relied on ectopically expressed lysenin, a sphingomyelin‐binding protein from the earthworm *Eisenia fetida* (Ellison *et al*, 2020). Since it remains unknown if and how cells sense cytosolically exposed sphingomyelin, we sought to identify and characterise the cellular sphingomyelin receptor(s) in the host cell cytosol. Here, we report that TECPR1 is a sphingomyelin‐binding protein that recruits the ATG5/ATG12‐E3 ligase to damaged membranes for the conjugation of LC3 to lipids. TECPR1‐dependent conjugation of LC3 occurs independently of ATG16L1, suggesting that the activity of the ATG5/ATG12‐E3 ligase is controlled by the interchangeable receptor subunits TECPR1 and ATG16L1 in a manner conceptually similar to certain multi‐subunit ubiquitin E3 ligases.

## Results

### TECPR1 detects exposure of sphingomyelin to the cytosol

**Figure 1:**
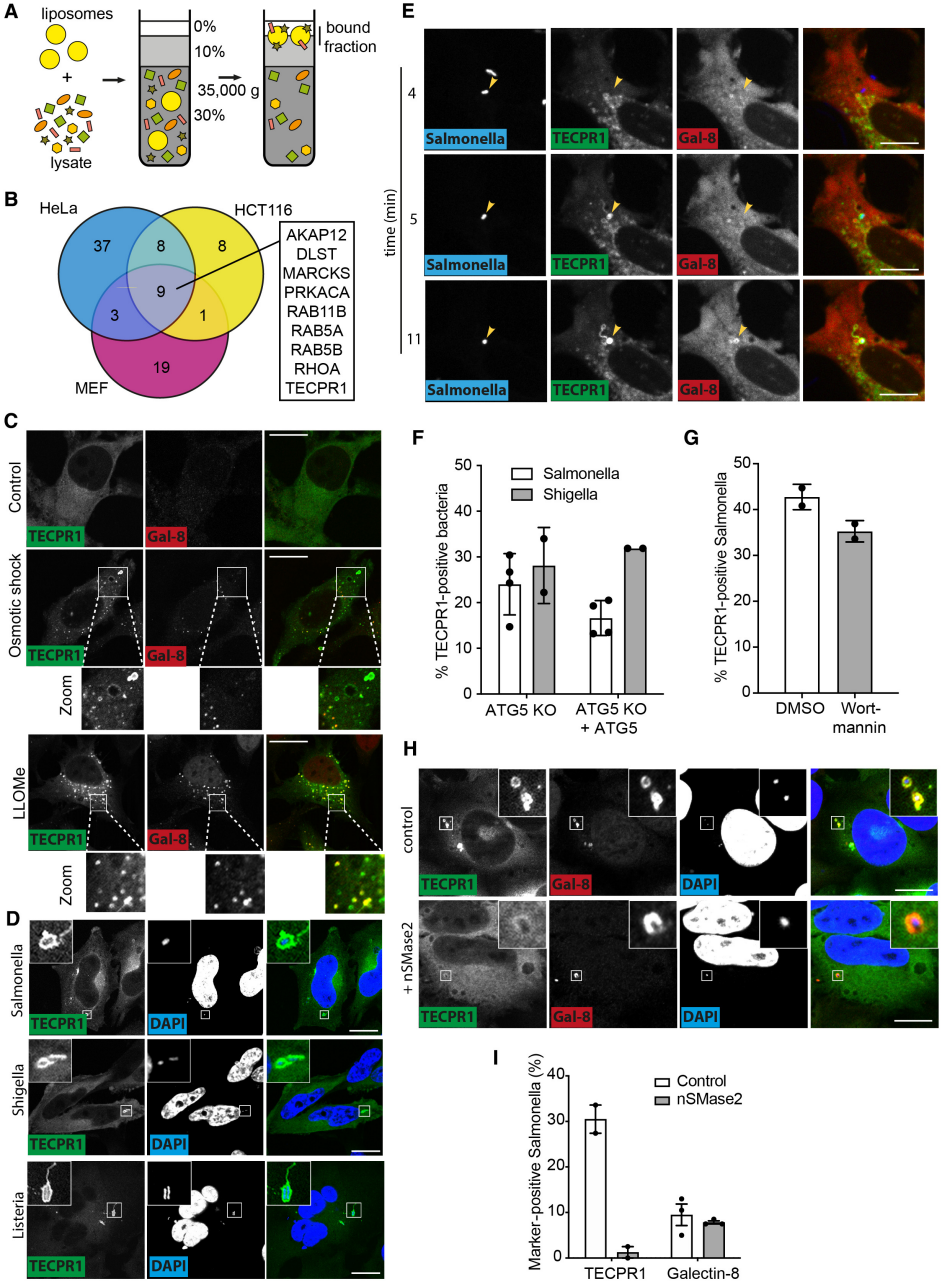
TECPR1 detects sphingomyelin on the cytosolic face of bacteria‐containing vacuoles ASchematic of liposome‐binding assay.BVenn diagram of liposome‐binding proteins identified in the cell lines shown with an enrichment value of > 1.4 in sphingomyelin versus control liposomes. Those proteins enriched in all three cell lines are indicated.C–H(C, D, H) Confocal micrographs of HeLa cells expressing GFP‐TECPR1 either alone or together with FLAG‐tagged neutral sphingomyelinase 2 (nSMase2 [H]) and treated with osmotic shock assay or LLOMe (C), or infected with the bacteria indicated (D) and fixed at 1 h (*S*. Typhimurium and *L. monocytogenes*) or 0.5 h (*S. flexneri*) postinfection. Cells were stained for Galectin‐8 and DNA (DAPI). Scale bar, 20 μm. (E) Selected frames from live imaging of HeLa cells expressing GFP‐TECPR1 and mCherry‐Galectin 8 infected with BFP‐expressing *S*. Typhimurium. Yellow arrowhead indicates a bacterium to which TECPR1 has been recruited prior to Galectin 8. Scale bar, 10 μm (F) Percentage of bacteria positive for mCherry‐TECPR1 in ATG5 knockout (KO) or ATG5‐complemented MEF cells at 1 h (*S*. Typhimurium) or 0.5 h (*S. flexneri*) postinfection. Mean ± SD of four (*S*. Typhimurium) or two (*S. flexneri*) independent experiments. *n* > 100 bacteria per coverslip. (G) Percentage of *S*. Typhimurium positive for GFP‐TECPR1 at 1 h postinfection in HeLa cells pretreated or not with 100 nM Wortmannin. Mean ± SD of two independent experiments. *n* > 100 bacteria per coverslip.IPercentage of *S*. Typhimurium positive for GFP‐TECPR1 or endogenous Galectin‐8 at 1 h postinfection in HeLa cells expressing FLAG‐nSMase2 or not. Mean ± SD of two (TECPR1) or three (Gal8) independent experiments. *n* > 100 bacteria per coverslip. Source data are available online for this figure.

**Figure EV1:**
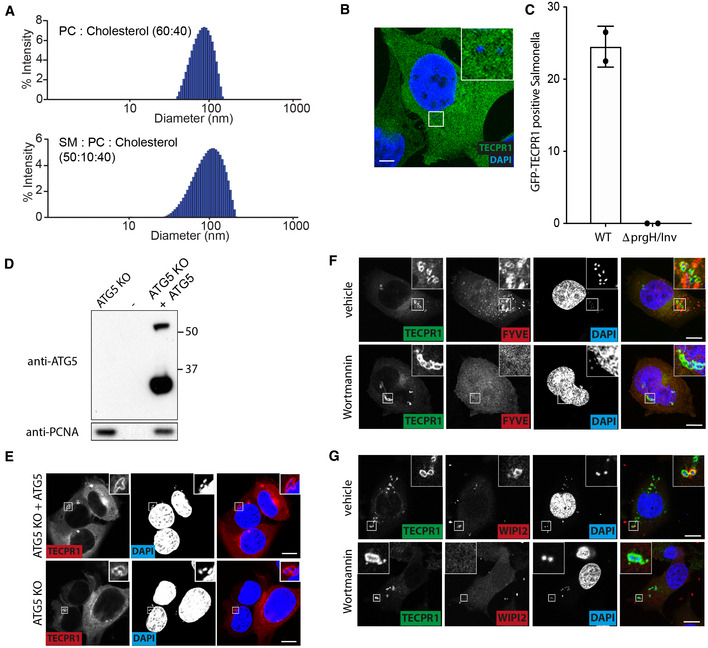
(corresponding to Fig 1). TECPR1 detects sphingomyelin on the cytosolic face of bacteria‐containing vacuoles AHistograms displaying the diameter of liposomes composed of phosphatidylcholine:cholesterol (60:40; top) or sphingomyelin:phosphatidylcholine: cholesterol (50:10:40; bottom), as measured by dynamic light scattering.BConfocal micrograph of HeLa cells expressing GFP‐TECPR1 infected with *S*. Typhimurium Δ*prgH* + *inv*, fixed at 1 h postinfection and stained with DAPI. Scale bar, 10 μm.CPercentage of *S*. Typhimurium WT or Δ*prgH* + *inv* positive for GFP‐TECPR1 in Hela cells fixed at 1 h postinfection. Mean ± SD of two independent experiments performed in duplicate. *n* > 100 bacteria per coverslip.DATG5‐deficient mouse embryonic fibroblasts were retrovirally transduced with an AU1‐tagged ATG5 construct or not, lysates prepared and analysed by SDS–PAGE and Western blotted with the indicated antibodies. Upper band in anti‐ATG5 blot is conjugated ATG5‐ATG12 while the lower is monomeric ATG5. PCNA serves as a loading control.EConfocal micrographs of ATG5‐deficient MEF cells, complemented with AU1‐ATG5 or not and expressing mCherry‐TECPR1, were infected with *S*. Typhimurium, fixed at 1 h postinfection and stained with DAPI to label bacteria. Scale bar, 20 μm.F, GConfocal micrographs of HeLa cells expressing GFP‐TECPR1 alone (G) or together with mCherry‐tagged FYVE domain from WDFY1 (F) were pretreated with 100 nM Wortmannin or mock control (DMSO) infected with *S*. Typhimurium for 1 h, fixed and stained with anti‐WIPI2 (G) and DAPI to label bacteria. Scale bar, 20 μm.

Bacteria‐mediated damage to the membrane of *Salmonella*‐ or *Shigella*‐containing vacuoles causes the transfer (“flipping”) of sphingomyelin from the luminal into the cytosolic leaflet of the membrane wherein, we propose here, sphingomyelin is recognised by specific cellular receptor(s) (Ellison *et al*, 2020). To identify such hypothetical sphingomyelin‐binding proteins, we developed a liposome‐based assay (Fig 1A). Liposomes formulated from either sphingomyelin:phosphatidylcholine:cholesterol (50:10:40 mol%) or phosphatidylcholine:cholesterol (60:40 mol%) as a control were mixed with detergent‐free lysates of human epithelial cells (HeLa, HCT116) or murine fibroblasts (Fig EV1A). Liposomes were purified by density gradient centrifugation and bound proteins identified by mass spectrometry. Subsequent calculation of enrichment values on sphingomyelin‐containing versus control liposomes (with a cut‐off value of > 1.4 fold) identified putative sphingomyelin‐binding proteins (Fig 1B). Among the nine proteins consistently enriched from all three cell types was TECPR1, a cytosolic protein previously suggested to have anti‐bacterial activity but not known to interact with sphingomyelin (Ogawa *et al*, 2011).

We expect putative cytosolic sphingomyelin receptors to mimic the sphingomyelin‐dependent recruitment of Lysenin to damaged host membranes (Ellison *et al*, 2020). We therefore investigated TECPR1 behaviour after either osmotic shock or treatment with the lysosomotropic agent L‐leucyl‐leucine methyl ester (LLOMe), known to cause sphingomyelin exposure and Lysenin accumulation on damaged endosomes and lysosomes, respectively (Ellison *et al*, 2020). We observed redistribution of GFP‐TECPR1 from a predominantly diffuse to a punctate cytosolic localisation typical for damaged vesicles (Fig 1C). Many of the smaller GFP‐TECPR1 punctae were also positive for Galectin‐8, indicative of glycan exposure upon catastrophic damage to the endosomal membrane, while GFP‐TECPR1^positive^ Galectin‐8^negative^ structures were often larger in size and had preserved a visible lumen, indicative of sphingomyelin exposure on vesicles that had maintained sufficient integrity to still shield intraluminal glycans. We propose that TECPR1, similar to Lysenin, detects minor, as well as catastrophic damage to both endosomes and lysosomes, characterised by TECPR1 recruitment in the absence or presence of Galectin‐8, respectively.

To test whether TECPR1 is also capable of detecting sphingomyelin exposure caused by pathogens, we investigated TECPR1 recruitment to bacteria that damage the limiting BCV membrane while gaining access to the host cytosol. TECPR1 was recruited to *Listeria monocytogenes*, *S. flexneri* and *S*. Typhimurium, that is, to both Gram‐positive and Gram‐negative bacteria (Fig 1D). Recruitment of TECPR1 to *S*. Typhimurium required the membrane‐penetrating SPI‐1 type 3 secretion system (Fig EV1B and C), consistent with a lack of sphingomyelin exposure in *S*. Typhimurium *ΔprgH + inv* (Ellison *et al*, 2020), a strain that invades epithelial cells by means of the *Yersinia* invasin gene, *Inv*, but remains confined to the SCV because of a nonfunctional SPI‐1 needle (Isberg & Falkow, 1985; Isberg *et al*, 1987). Since SPI‐1‐mediated damage of the SCV membrane exposes sphingomyelin prior to glycans (Ellison *et al*, 2020), we tested whether recruitment of TECPR1 similarly precedes that of Galectin‐8. Indeed, live‐cell imaging of *S*. Typhimurium infection showed sequential recruitment of TECPR1 and Galectin‐8 (Fig 1E; Movie EV1).

The recruitment of TECPR1 to immotile *S. flexneri* Δ*icsA* has been reported to be dependent on ATG5, a well‐established TECPR1 interactor, and on the upstream autophagy regulator WIPI‐2 (Ogawa *et al*, 2011). However, we found that neither protein was essential for the recruitment of TECPR1 to either *S. flexneri* or *S*. Typhimurium, as TECPR1 recruitment was maintained in ATG5^−/−^ MEFs (Figs 1F and EV1D and E) and in cells treated with the PI3 kinase inhibitor wortmannin, which prevented recruitment of the PI(3)P reporter mCherry‐FYVE and the PI(3)P‐binding protein WIPI‐2 to bacteria‐containing vacuoles (Figs 1G and EV1F and G). Thus, neither ATG5 nor the presence of WIPI‐2 at BCVs is required for the recruitment of TECPR1. By contrast, ectopic expression of neutral sphingomyelinase 2 (SMPD3), which removes sphingomyelin from the cytosolic leaflet of damaged membranes (Ellison *et al*, 2020), prevented recruitment of TECPR1 but not galectin‐8 (Fig 1H and I), thereby revealing cytosolic exposure of sphingomyelin as essential for TECPR1 recruitment to damaged bacteria‐containing vacuoles.

### The N‐terminal DysF domain of TECPR1 binds sphingomyelin

**Figure 2:**
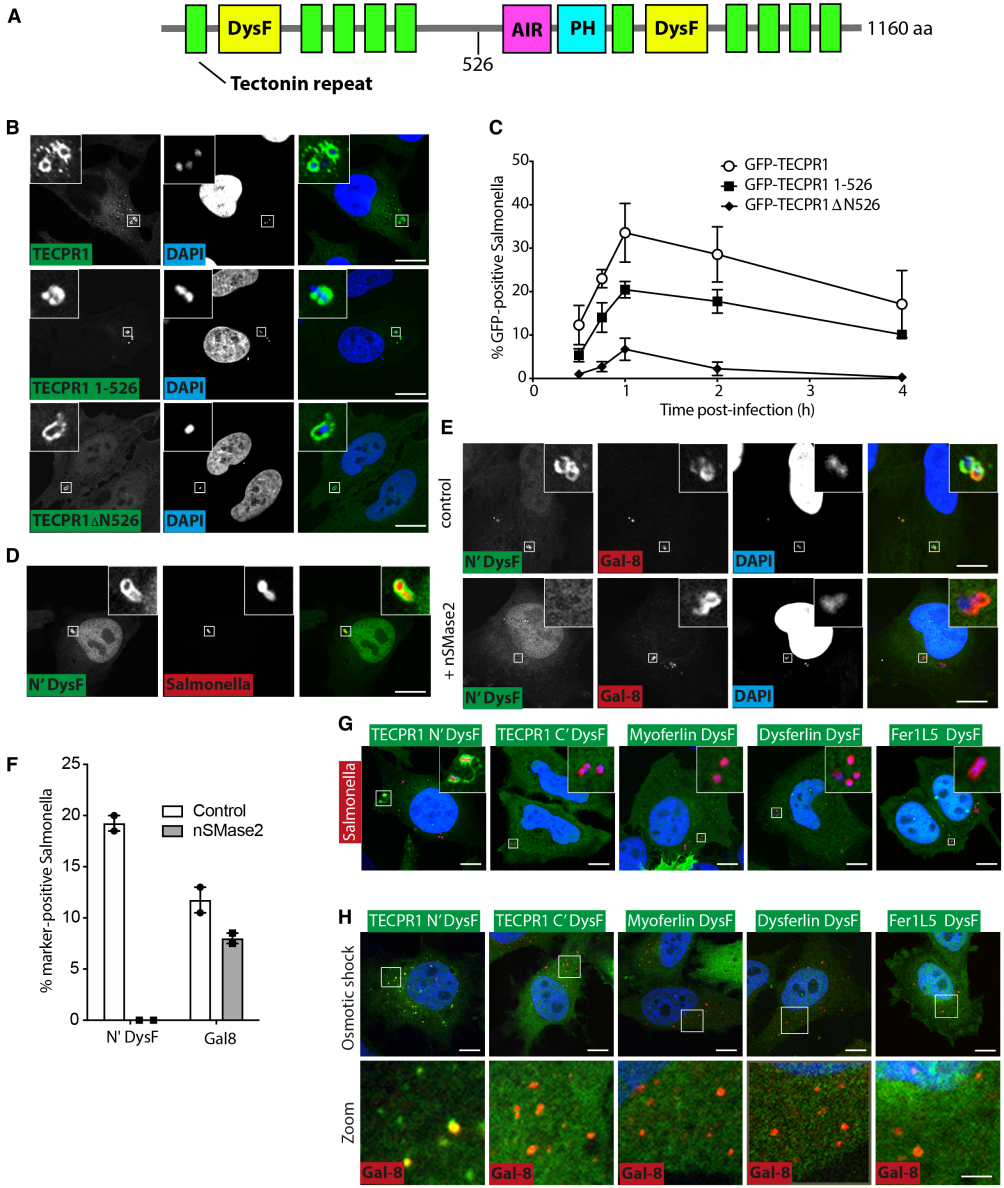
The N‐terminal DysF domain of TECPR1 detects sphingomyelin ASchematic of TECPR1 domain structure. AIR, ATG5‐interacting region; PH, Plekstrin homology domain. Tectonin repeat indicated.BConfocal micrographs of HeLa cells expressing the indicated GFP‐tagged TECPR1 constructs, infected with *S*. Typhimurium, fixed at 1 h postinfection and DNA stained with DAPI. Scale bar, 20 μm.CPercentage of *S*. Typhimurium positive for the indicated constructs in HeLa cells, fixed at the indicated timepoints postinfection. Mean ± SEM of 3–4 independent experiments performed in duplicate. *n* > 100 bacteria per coverslip.DConfocal micrographs of HeLa cells expressing TECPR1 N‐terminal (N′) DysF‐GFP infected with mCherry‐expressing *S*. Typhimurium, fixed at 30 min postinfection. Scale bar, 20 μm.EConfocal micrographs of HeLa cells expressing TECPR1 N′ DysF‐GFP with or without FLAG‐nSMase2, infected with *S*. Typhimurium, fixed at 30 min postinfection and stained with anti‐Galectin 8 antibody. DNA stained with DAPI. Scale bar, 20 μm.FPercentage of *S*. Typhimurium positive for N′ DysF‐GFP in Hela cells, expressing FLAG‐nSMase 2 or not, fixed at 30 min postinfection. Mean ± SD of two independent experiments performed in duplicate.G, HConfocal micrographs of HeLa cells expressing the indicated GFP‐tagged DysF domains, infected with mCherry‐*S*. Typhimurium and fixed at 30 min postinfection (G) or treated with osmotic shock assay, fixed and stained with anti‐Galectin 8 antibody (H). The bottom row in H depicts an enlarged version of the boxed region shown above. Scale bar, 20 μm. Source data are available online for this figure.

**Figure EV2:**
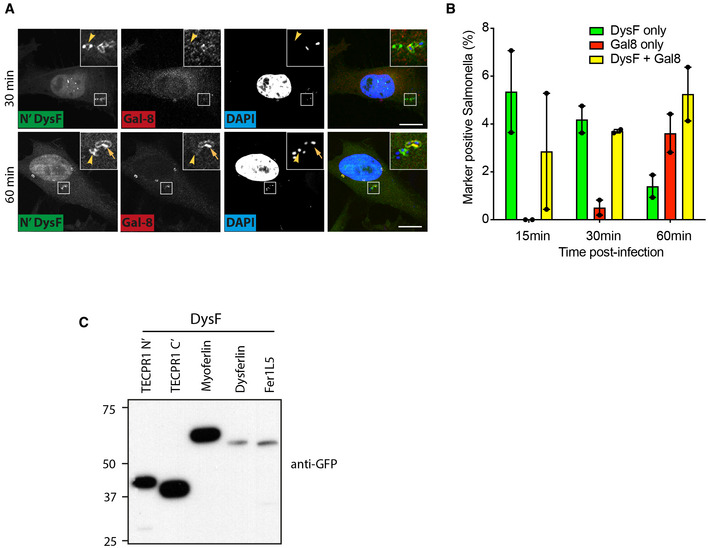
(corresponding to Fig 2). The N‐terminal DysF domain of TECPR1 detects sphingomyelin AConfocal micrographs of Hela cells expressing N‐terminal DysF domain of TECPR1 (N'DysF) as a C‐terminal GFP fusion were infected with *S*. Typhimurium, fixed at the indicated times postinfection, stained with anti‐Galectin 8 and DAPI. Arrowhead indicates bacteria to which DysF‐GFP but not Galectin 8 is recruited, whereas arrow indicates those to which both proteins are recruited. Scale bar, 20 μm.B Percentage of *S*. Typhimurium positive for N‐terminal DysF‐GFP and/or anti‐Galectin 8 in HeLa cells fixed at the indicated times postinfection. Mean ± SD of two independent experiments performed in duplicate.CHela cells stably expressing the indicated GFP‐tagged DysF constructs were prepared for SDS–PAGE and Western blotted with anti‐GFP antibody.

TECPR1 is comprised of a central ATG5‐interacting region (AIR) and an adjacent pleckstrin homology (PH) domain with affinity for PI(3)P and/or PI(4)P (Chen *et al*, 2012; Wetzel *et al*, 2020; Fig 2A). These domains are flanked by two 5‐bladed beta‐propellor domains, whose blades are formed from WD40‐like TECPR repeats. Inserted between the first and second blades of each propellor lie DysF domains, named after their occurrence in the protein Dysferlin and so far without known functions. To determine how TECPR1 binds *Salmonella*‐containing vacuoles, we performed a deletion analysis of the protein. Recruitment of full‐length TECPR1 to *Salmonella*‐containing vacuoles peaked at 1 h p.i. and was sustained over several hours (Fig 2B and C). We found that nonoverlapping N‐terminal and C‐terminal fragments of TECPR1 both accumulated around *S*. Typhimurium, suggesting two independent modes of recruitment for TECPR1, although the N‐terminal construct accounted for the majority of TECPR1 recruitment (Fig 2B and C). Further deletion analysis of the N‐terminal fragment was thought ill‐advised due to the likely collapse of the propellor structure upon deletion of any of its constituent blades. However, we observed that the N‐terminal DysF domain (N'DysF) on its own was sufficient for recruitment to *S*. Typhimurium (Fig 2D). Similar to full‐length TECPR1, the recruitment of N'DysF occurred prior to Galectin‐8 (Fig EV2A and B) and was also abolished by co‐expression of neutral sphingomyelinase 2 (Figs 1H and I, and 2E and F), thereby demonstrating that the N‐terminal DysF domain is the minimal sphingomyelin‐binding domain of TECPR1.

In addition to TECPR1, the human genome encodes three further DysF domain‐containing proteins, namely Dysferlin, Myoferlin and Fer1L5. Ferlins are an ancient protein family involved in vesicle fusion and membrane repair (Bulankina & Thoms, 2020; Dominguez *et al*, 2022). Mutations in the DysF domain of Dysferlin cause limb girdle muscular dystrophy type 2B2 and Miyoshi myopathy (Izumi *et al*, 2020; Ivanova *et al*, 2022). We therefore tested which human DysF domains bind sphingomyelin. In contrast to TECPR1, where the two DysF are spatially separated, the Ferlin DysF domains occur as nested repeats, with one DysF domain inserted into a loop of another DysF domain, a.k.a. the inner and the outer DysF domains, respectively (Dominguez *et al*, 2022). We thus compared the entire dual DysF domains from Ferlins with individual N‐ and C‐terminal DysF domains from TECPR1. We found that only the N‐terminal DysF domain of TECPR1 accumulated on damaged SCVs and on osmotically damaged endosomes (Figs 2G and H, and EV2C), revealing the N‐terminal DysF of TECPR1 to be the only human DysF domain capable of recognising sphingomyelin exposed on damaged endomembranes.

**Figure 3:**
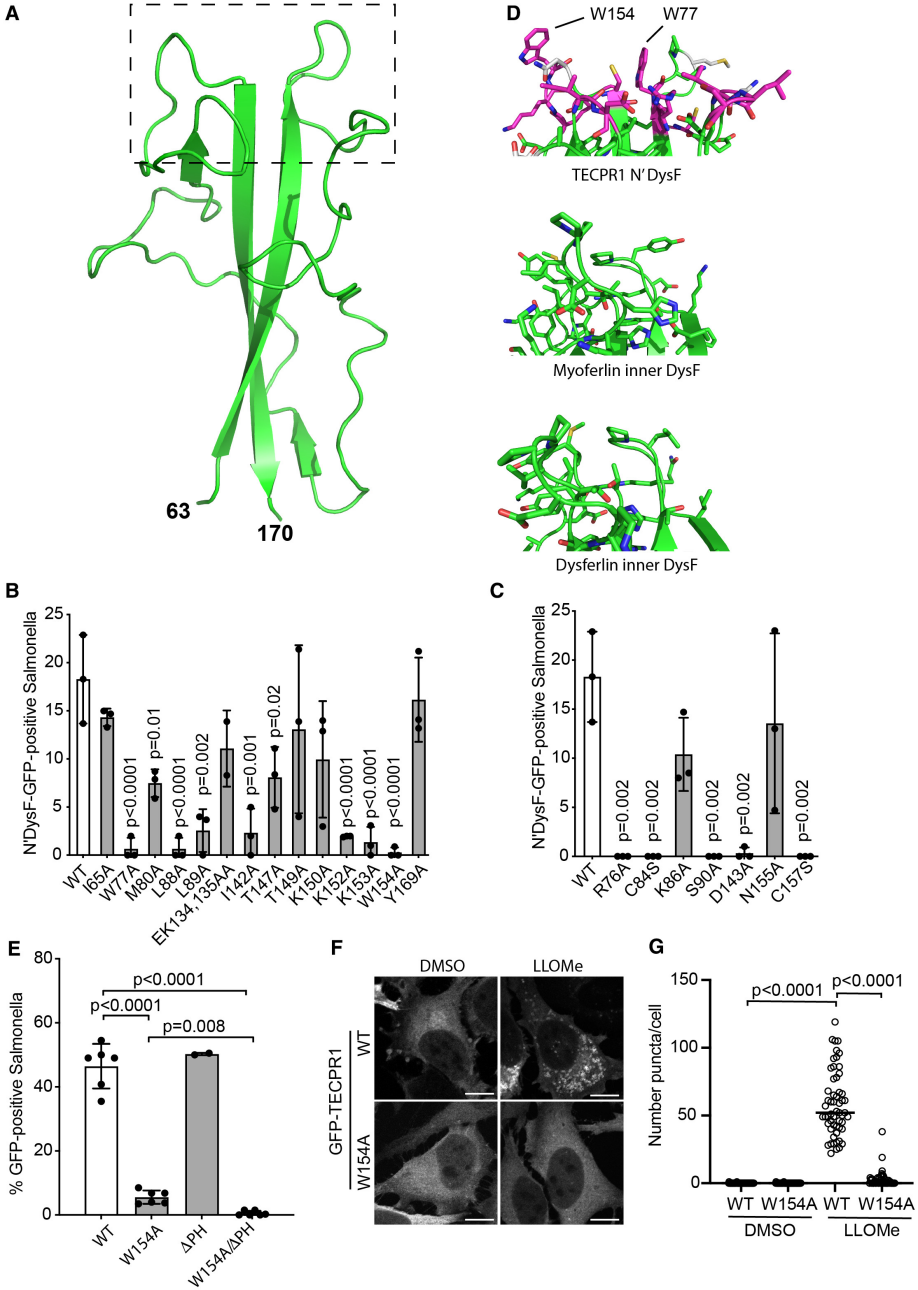
Identification of the sphingomyelin‐binding site in the N‐terminal DysF domain ARibbon diagram of the crystal structure of the N‐terminal DysF domain with amino acid positions corresponding to full‐length TECPR1 indicated.B, CPercentage of *S*. Typhimurium positive for the indicated GFP‐tagged N‐terminal DysF constructs at 30 min postinfection. Mean ± SD of two (EK134,135AA) or three independent experiments. *n* > 100 bacteria per coverslip. *P*‐value from one‐way ANOVA with Dunnett's multiple comparison test.DRibbon diagram of the distal tip of N‐terminal DysF domain structure (boxed area in [A]) showing those residues required for recruitment to *S*. Typhimurium depicted with side chains in magenta and those not in grey (top). The two exposed tryptophan residues are highlighted. The comparable region from the structures of the inner DysF domains of Myoferlin (PDB:2K2O; middle) and Dysferlin (PDB:4CAH; bottom) are depicted.EPercentage of *S*. Typhimurium positive for indicated GFP‐TECPR1 constructs in HeLa cells at 30 min postinfection. Mean ± SEM of two (ΔPH) or six independent experiments performed in duplicate. *n* > 100 bacteria per coverslip. *P*‐value from one‐way ANOVA with Tukey's multiple comparison test.FConfocal micrographs of HeLa cells expressing either GFP‐TECPR1 WT or W154A and treated with LLOMe or DMSO vehicle control for 10 min and fixed. Scale bar, 20 μm.GNumber of GFP‐positive puncta per cell was enumerated in samples depicted in (F). Data from 50 cells from a single experiment, representative of two. *P*‐value from one‐way ANOVA with Tukey's multiple comparison test. Source data are available online for this figure.

**Figure EV3:**
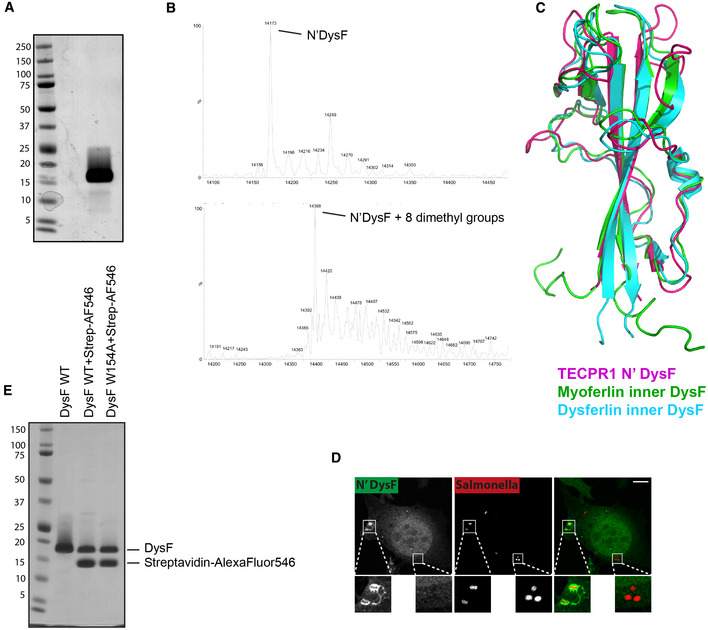
(corresponding to Fig 3). Identification of the sphingomyelin‐binding site in the N‐terminal DysF domain ACoomassie stain of SDS–PAGE gel of recombinant TECPR1 N‐terminal DysF domain purified from *E. coli*.BMass spectrometry of recombinant TECPR1 N‐terminal DysF protein before (top) and after (bottom) reductive methylation. Note the shift in mass of major species from 14,173 to 14,398 Da (225 Da) corresponding to eight dimethyl groups attached to seven lysine and N‐terminal methionine residues.COverlay of the crystal structure of TECPR1 N‐terminal DysF domain and that of the NMR structure of the inner DysF domain of myoferlin (PDB: 2K2O) and the crystal structure of the inner DysF domain of Dysferlin (PDB: 4CAH).DConfocal micrographs of Hela cells expressing N'DysF‐GFP infected with mCherry‐Salmonella for 30 min and fixed. Micrograph is same as that shown in Fig 2G. Insets depict SCVs that are scored as being either positive (left) or negative for DysF‐GFP (right). Scale bar, 20 μm.ECoomassie stain of SDS–PAGE gels of recombinant N'DysF WT or W154A protein before and after labelling with streptavidin^AlexaFluor46^.

To understand how the N‐terminal DysF domain of TECPR1 recognises sphingomyelin, we solved its crystal structure. Our initial attempts to generate crystals proved fruitless, possibly due to the abundance of positively charged amino acids in the domain that may prevent crystallisation. However, upon reductive methylation of primary amino groups, confirmed by matrix‐assisted laser desorption/ionisation (MALDI) time‐of‐flight (TOF) and in‐source decay (ISD) mass spectrometry (Fig EV3A and B), the N‐terminal DysF domain of TECPR1 yielded diffracting crystals, the structure of which was solved to 1.9 Å resolution by molecular replacement with the NMR structure of the inner DysF domain of myoferlin (Fig 3A, Table 1). The N‐terminal DysF domain of TECPR1 represents an elongated finger‐shaped domain, which, overall, bore high similarity to the inner DysF domains of myoferlin and dysferlin (Fig EV3C) (Patel *et al*, 2008; Sula *et al*, 2014a). The N‐terminal DysF domain of TECPR1 is comprised of a central pair of antiparallel beta strands connected via a long loop that wraps around the beta‐sheet and covers large parts of the domain surface. The loop is precisely positioned and held in place through planar stacking interactions between multiple tryptophan and arginine residues (specifically Trp 116, 118, 122, 138 and Arg 159, 161, 165, 167), a feature also found in Ferlin DysF domains (Patel *et al*, 2008; Sula *et al*, 2014a).

**Table 1:** Data collection statistics. Numbers in brackets are for the highest resolution bin.

	Tecpr1 60–173
Data collection	
Beamline	Diamond I04‐1
Space group	P 65
*a*, *b*, *c* (Å)	75.72, 75.72, 122.86
*α*, *β*, *γ* (°)	90.00, 90.00, 120.00
Wavelength	0.9200
Resolution (Å)	44.83–1.90 (1.94–1.90)
*R*_merge_	0.09 (0.73)
*I*/σ*I*	12.0 (2.3)
Completeness (%)	99.8 (100)
Redundancy	5.9 (5.9)
Refinement	
Resolution (Å)	44.83–1.90
No. reflections	31,331
*R*_work_/*R*_free_	16.6/20.8
No. atoms	
Protein	3,619
Ligand/ion	27
Water	304
*B*‐factors	
Wilson *B*	24.89
Protein	34.5
Ligand/ion	53.9
Water	37.0
R.m.s deviations	
Bond lengths (Å)	0.011
Bond angles (°)	1.074
Ramachandran statistics (favoured/allowed/outliers)	97.87/2.13/0.00

To identify the sphingomyelin‐binding site in the N‐terminal DysF domain of TECPR1, we mutated several surface‐exposed residues and examined their effect on the recruitment of the GFP‐tagged N'DysF domain to *Salmonella*‐containing vacuoles. SCVs were scored as being either positive or negative for recruitment of N'DysF‐GFP constructs (Fig EV3D). Mutations of W77, L88, L89, I142, K152, K153 and W154 into alanine all abrogated recruitment, while mutations of I65, M80, E134, K135, T147, T149, K150, Y169 had little or no effect (Fig 3B). We conclude that the functionally important residues cluster at the distal end of the domain. Subsequent mutations of nearby residues R76, S90 and D143 into alanine and C84 and C157 into serine also abolished recruitment (Fig 3C). Together these mutants identify a binding site located at the distal end of the domain, that is, the tip of the DysF finger. Consistent with the zwitterionic nature of sphingomyelin, the binding site is characterised by positively (R76, K152, K153) and negatively charged (D143), as well as hydrophobic residues. Notably, W77 and W154 are solvent‐exposed and upon binding may become inserted into target membranes (Fig 3D). A comparison of the sphingomyelin‐binding site in the N'DysF domain of TECPR1 to the equivalent region in the inner DysF domains of myoferlin and dysferlin reveals little conservation of loop structures and exposed side chains (Fig 3D), thus explaining why the latter domains are not recruited to damaged membranes (Fig 2G).

To assess the contribution of the sphingomyelin‐binding site to the detection of membrane damage by full‐length TECPR1, we generated TECPR1 W154A, which we found was severely impaired in its recruitment to both SCVs (Fig 3E) and lysosomes damaged by LLOMe (Fig 3F and G). The remaining minor activity in TECPR1 W154A depends on its PH domain as the recruitment of a double mutant (TECPR1 W154A/ΔPH) to SCVs was completely abrogated (Fig 3E). We conclude that the recruitment of TECPR1 to damaged membranes is overwhelmingly driven by sphingomyelin and the N‐terminal DysF domain, with only a minor contribution from its PH domain.

**Figure 4:**
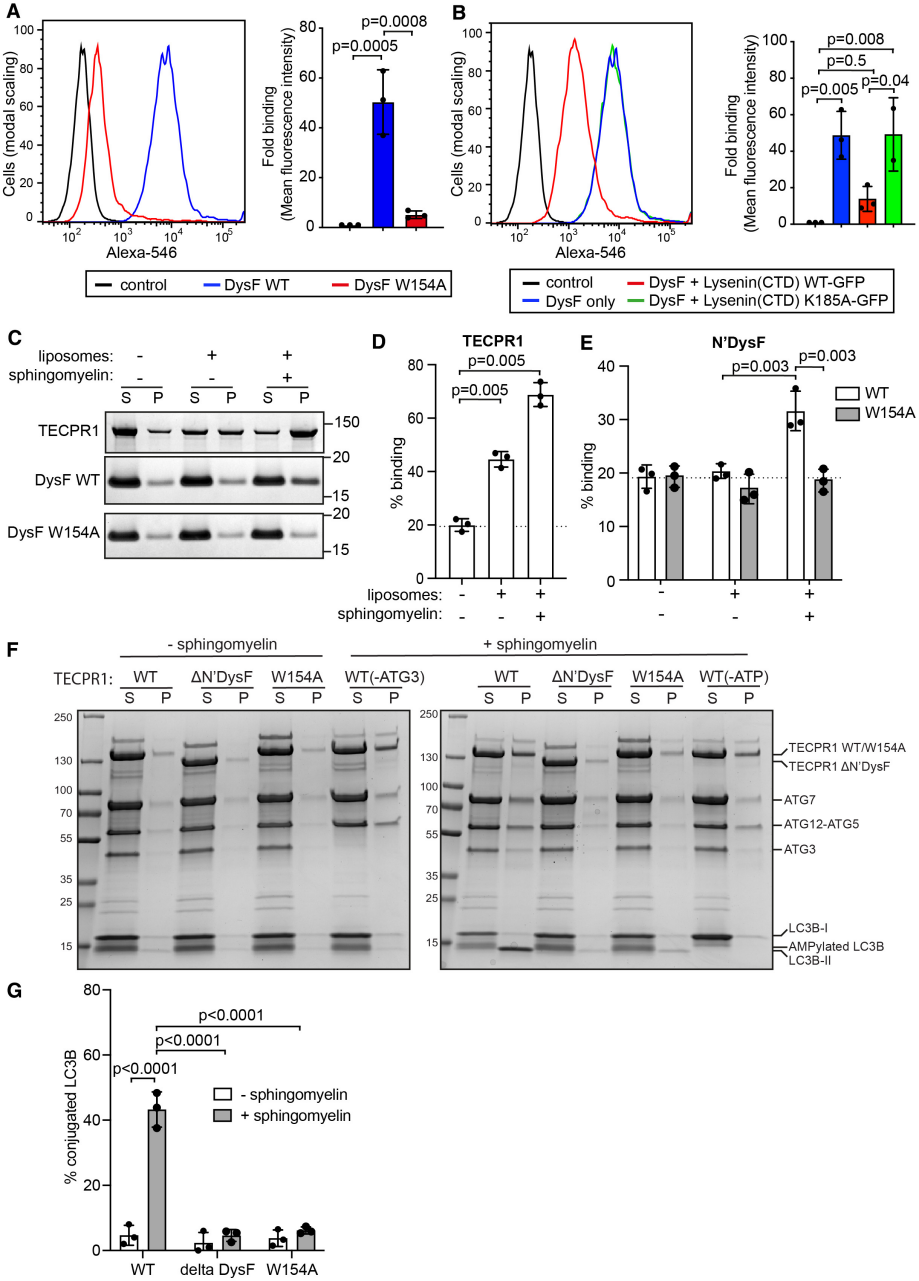
TECPR1 in complex with ATG5/ATG12 catalyses the conjugation of LC3 to lipids in sphingomyelin‐displaying membranes A, BBinding of the indicated streptavidin^Alexa546^‐conjugated N‐terminal DysF proteins to Hela cells in the presence or absence of a 10‐fold molar excess of indicated Lysenin^CTD^‐GFP proteins. Representative histograms of DysF protein binding (left) and mean fluorescence intensity ± SD of three independent experiments (right). *P*‐value from one‐way ANOVA with Tukey's multiple comparison test.C–ELiposome sedimentation assay. The indicated proteins were incubated with either liposomes (containing sphingomyelin or not) or buffer only, pelleted by centrifugation and equal portions of supernatant and pellet fractions analysed by SDS–PAGE and Coomassie staining. Representative gels depicted (C). Fraction of TECPR1 (D) or DysF WT or W154A (E) proteins present in pellet under the indicated conditions, expressed as % binding. Note pelleting in the absence of liposomes likely due to the affinity of proteins for tube walls. Mean ± SD of three independent experiments. *P‐*value from one‐way ANOVA with Tukey's multiple comparison test.F
*In vitro* LC3 lipid conjugation assay. Liposomes containing sphingomyelin or not were incubated with LC3 lipid conjugation machinery: ATG7 (E1), ATG3 (E2), ATG5‐ATG12 (E3), LC3B, MgCl_2_, ATP and the indicated TECPR1 proteins. Control reactions without ATG3 or ATP were included. Mixes were incubated at 37°C for 3 h, centrifuged and equal fractions of both supernatant and pellet analysed by SDS–PAGE and Coomassie staining. Lipidated LC3B‐II appears in pellet fraction whereas both soluble LC3B‐I and AMPylated LC3B are in the supernatant, the latter of the same apparent molecular weight as LC3B‐II.GFraction of LC3B protein present in pellet from complete *in vitro* LC3 lipid conjugation assay with the indicated TECPR1 proteins in either absence or presence of sphingomyelin. Mean ± SD from three independent experiments. *P*‐value from one‐way ANOVA with Tukey's multiple comparison test. Source data are available online for this figure.

To test whether the N‐terminal DysF domain interacts directly with sphingomyelin, we purified the recombinant domain from *E. coli* and assessed binding to both naturally occurring and reconstituted sphingomyelin‐containing membranes. Fluorescently‐labelled wild‐type, but not W154A mutant, N'DysF domain bound strongly to the surface of mammalian cells (Figs 4A and EV3E). Binding was outcompeted by the sphingomyelin‐binding C‐terminal domain of wild‐type Lysenin (161–297) but not by a sphingomyelin‐insensitive K185A mutant (Ellison *et al*, 2020; Fig 4B). In liposome‐binding assays, the interaction of recombinant full‐length TECPR1 with membranes was potentiated by the presence of sphingomyelin (Fig 4C and D). Similarly, the N'DysF domain is preferentially bound to sphingomyelin‐containing liposomes. Binding was abrogated in the W154A mutant (Fig 4C and E). Taken together, we conclude that the N‐terminal DysF domain of TECPR1 acts as a sphingomyelin sensor that enables TECPR1 to accumulate on stressed endomembranes with sphingomyelin exposure in their cytosolic leaflet.

### TECPR1 conjugates LC3 to sphingomyelin‐containing liposomes

During macroautophagy, cells conjugate LC3s and GABARAPs to double‐membrane structures known as phagophores while certain noncanonical autophagy pathways coalesce on the conjugation of LC3/GABARAP to single membranes (Florey *et al*, 2014; Martinez *et al*, 2015; Nieto‐Torres *et al*, 2021; Ulferts *et al*, 2021). In either pathway, LC3/GABARAP conjugation is catalysed by a trimeric E3 ligase complex comprised of ATG5, ATG12 and ATG16L1. Specificity for target membranes is generally provided by ATG16L1, for example in macroautophagy through interaction with WIPI2 that marks sites of phagophore formation (Dooley *et al*, 2018). However, the ability of TECPR1 to compete with ATG16 for binding to ATG5/ATG12 (Chen *et al*, 2012; Kim *et al*, 2014) and the sphingomyelin‐dependent recruitment of TECPR1 to damaged endomembranes suggest that the TECPR1/ATG5/ATG12 complex may serve as a sphingomyelin‐dependent E3 ligase on damaged endomembranes.

To test whether TECPR1 directs E3 ligase activity of the ATG5/ATG12 complex towards sphingomyelin‐containing membranes, we reconstituted the conjugation of LC3B *in vitro* using liposomes containing phosphatidylethanolamine as substrate for LC3 lipidation (Fracchiolla *et al*, 2020). Reactions were comprised of recombinant purified ATG7 and ATG3 to serve as E1 and E2 enzymes, respectively, as well as ATG5/ATG12 and TECPR1. In the presence of sphingomyelin‐containing liposomes, LC3B became lipid‐modified, as revealed by the appearance of a faster migrating band in the pellet fraction, which is commonly referred to as LC3B‐II (Fig 4F and G). The band of similar electrophoretic motility in the supernatant fraction represents AMPylated LC3B as its formation required the presence of ATG7 and ATP but not ATG3 or liposomes. No lipid‐modified LC3B‐II was formed on liposomes deficient in sphingomyelin or when either ATP or ATG3 were omitted from the reaction. Both TECPR1 ΔN'DysF (65–170) and TECPR1 W154A failed to lipidate LC3B. We conclude that TECPR1 promotes the conjugation of LC3B to sphingomyelin‐containing liposomes, thereby identifying the TECPR1/ATG5/ATG12 complex as a sphingomyelin‐activated E3 ligase for the conjugation of LC3B.

### TECPR1 mediates ATG16L1‐independent conjugation of LC3 at *Salmonella*‐containing vacuoles

**Figure 5:**
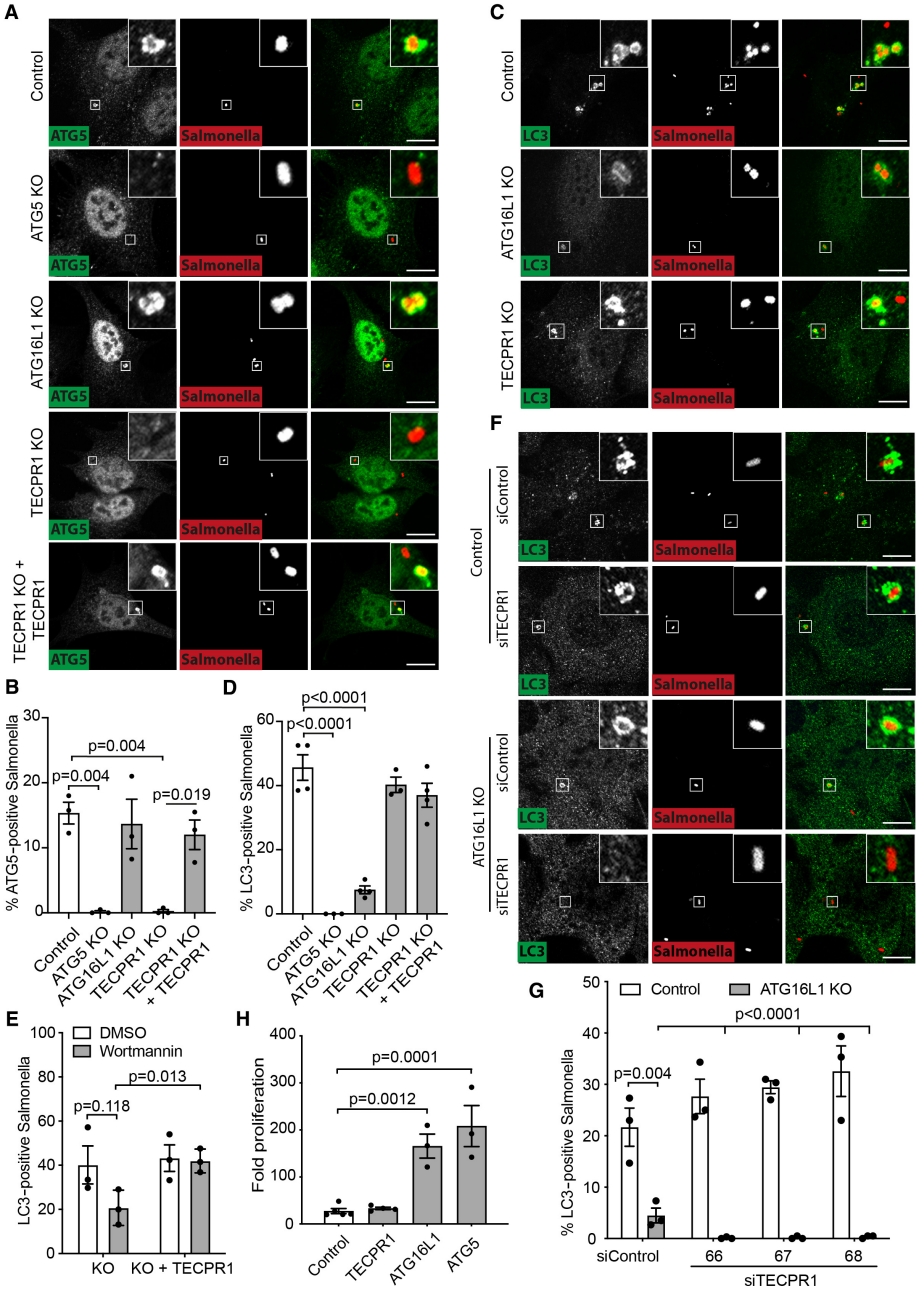
TECPR1 recruits ATG5 to *Salmonella*‐containing vacuoles for ATG16L1‐independent LC3 conjugation A–D(A, C) Confocal micrographs of indicated MEF cell lines infected with mCherry‐expressing *S*. Typhimurium, fixed at 30 min postinfection and stained with anti‐ATG5 antibody (A) or anti‐LC3 antibody (C). Scale bar, 20 μm. (B, D) Percentage of *S*. Typhimurium positive for endogenous ATG5 (B) or LC3 (D) in indicated MEF cells at 30 min postinfection. Mean ± SEM of 3 (B) or 3–4 (D) independent experiments performed in duplicate. *n* > 200 bacteria per coverslip. *P*‐value from one‐way ANOVA with Tukey's multiple comparison test.EPercentage of *S*. Typhimurium positive for endogenous LC3 in indicated MEF cells treated with 100 nM Wortmannin or DMSO vehicle control, fixed at 1 h postinfection. Mean ± SEM of three independent experiments performed in duplicate. *n* > 100 bacteria per coverslip. *P*‐value from one‐way ANOVA with Tukey's multiple comparison test.FConfocal micrographs of either control of ATG16L1‐deficient MEF cells, transfected with either control or TECPR1 siRNA (siRNA no. 66 depicted), infected with mCherry‐expressing *S*. Typhimurium for 30 min, fixed and stained with anti‐LC3 antibody. Scale bar, 20 μm.GPercentage of *S*. Typhimurium positive for LC3 in either control or ATG16L1‐deficient MEF cells transfected with indicated siRNAs and infected with mCherry‐expressing *S*. Typhimurium for 30 min. Mean ± SEM of three independent experiments performed in at least duplicate and enumerated after blind labelling of coverslips. *P*‐value from one‐way ANOVA with Dunnett's multiple comparison test.HFold intracellular proliferation of *S*. Typhimurium in indicated MEF cells as assessed by colony forming unit assay, expressed as a ratio of intracellular bacteria present at 6 h versus 1 h. Mean ± SEM of three (ATG16L1 KO, ATG5 KO), four (TECPR1 KO) or five (Control) independent experiments. *P*‐value from one‐way ANOVA with Tukey's multiple comparison test. Source data are available online for this figure.

**Figure EV4:**
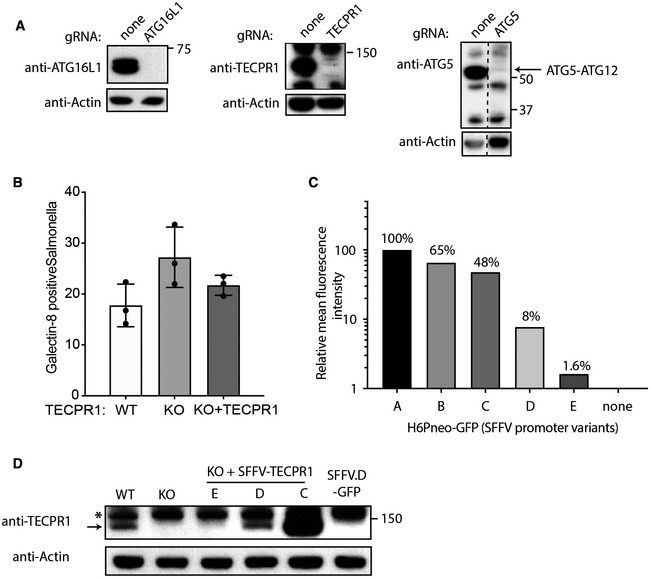
(corresponding to Fig 5). TECPR1 recruits ATG5 to Salmonella‐containing vacuoles for ATG16L1‐independent LC3 conjugation AWestern blot of lysates from MEF cells firstly lentivirally transduced to stably express Cas9 followed by stable lentiviral expression of indicated gRNAs for 7 days and blotted with antibodies shown. Hatched line in (F) denotes where intervening, irrelevant lanes of the blot were excised.BPercentage of *S*. Typhimurium positive for anti‐Galectin 8 in TECPR1 WT MEF cells, KO or KO complemented with TECPR1 construct fixed at 1 h postinfection. Mean + SEM of three independent experiments performed in duplicate. Statistical significance, based on a one‐way ANOVA with the Tukey's multiple comparison test, was not reached between any of the groups.CRelative expression level of GFP in HeLa cells stably transduced with H6Pneo lentivirus, selected with G418 and measured by flow cytometry. H6Pneo harbours GFP under the control of one of five different, progressively shorter portions of the spleen focus‐forming virus (SFFV) promoter (A–E) as described in Materials and Methods.DWestern blot of TECPR1 WT or KO MEFs or KO complemented with TECPR1 or GFP control from indicated H6P‐based lentiviruses harbouring SFFV promoters corresponding to those in (B). Arrow indicates specific band corresponding to TECPR1 at 135 kDa, and star denotes nonspecific band. Actin serves as loading control.

The identification of a novel sphingomyelin‐activated TECPR1/ATG5/ATG12 E3 ligase prompted us to investigate how ATG5 is recruited to *Salmonella*‐containing vacuoles. To this end, isogenic polyclonal MEFs deficient in ATG5, ATG16L1 or TECPR1 were generated by CRISPR‐Cas9 (Fig EV4A). ATG16L1‐deficient MEFs had unaltered recruitment of ATG5 to SCVs, while those deficient in TECPR1 failed to recruit ATG5 (Fig 5A and B) despite similar cytosolic entry of bacteria between the two conditions (Fig EV4B). The phenotype was rescued through complementation with TECPR1 expressed at near endogenous levels (Figs 5A and B, and EV4C and D). We conclude that TECPR1 is essential for the recruitment of ATG5 to *Salmonella*‐containing vacuoles, with little or no contribution from ATG16L1. By contrast, the loss of ATG16L1 but not TECPR1 substantially impaired the recruitment of LC3 (Fig 5C and D). As expected, the lack of ATG5 completely abrogated LC3 recruitment. Thus, while TECPR1 is uniquely required for the recruitment of ATG5 to SCVs, the majority of LC3 recruitment occurs in an ATG16L1‐dependent manner. We therefore speculated that any conjugation of LC3 by means of TECPR1‐localised ATG5 on SCVs may be masked by redundancy with the ATG16L1 pathway. Consistent with redundant pathways for LC3 conjugation, we found that wortmannin, which inhibits the formation of PI(3)P and prevented recruitment of the ATG16L1‐binding autophagy factor WIPI2 (Fig EV1F and G), reduced recruitment of LC3 to SCVs by approximately 50% only when TECPR1 was absent from the cell (Fig 5E). We next directly assessed redundancy between TECPR1 and ATG16L1 by analysing the TECPR1‐dependent recruitment of LC3 to SCVs in control or ATG16L1 KO cells. While the majority of LC3‐positive SCVs were formed in an ATG16L1‐dependent process, about one‐quarter of LC3‐positive SCVs were created in a TECPR1‐dependent (and ATG16L1‐independent) manner (Fig 5F and G). We conclude that TECPR1 in complex with ATG5/ATG12 conjugates LC3 to *Salmonella*‐containing vacuoles although the majority of LC3 recruitment is dependent on ATG16L1. However, since ATG16L1 does not mediate recruitment of the essential ATG5 subunit to *Salmonella*‐containing vacuoles, we propose that ATG16L1 in complex with ATG5‐ATG12 conjugates LC3 to membranes at a location distant from the SCV and that membranes harbouring LC3 lipidated in a ATG16L1‐dependent manner are subsequently trafficked to the damaged SCV.

We next tested whether TECPR1 affects the fate of *S*. Typhimurium in host cells. However, no significant difference in the proliferation of *S*. Typhimurium between control and TECPR1 KO cells was observed, in contrast to marked hyperproliferation in either ATG5‐ or ATG16L1‐deficient cells (Fig 5H). We conclude that TECPR1, when targeted towards damaged sphingomyelin‐positive endomembranes, recruits ATG5 and functions as an alternative E3 ligase for LC3 but that the ATG16L1‐mediated pathway is sufficient to restrict bacterial replication, at least in this cultured cell system.

### TECPR1 can damage SCV membranes

**Figure 6:**
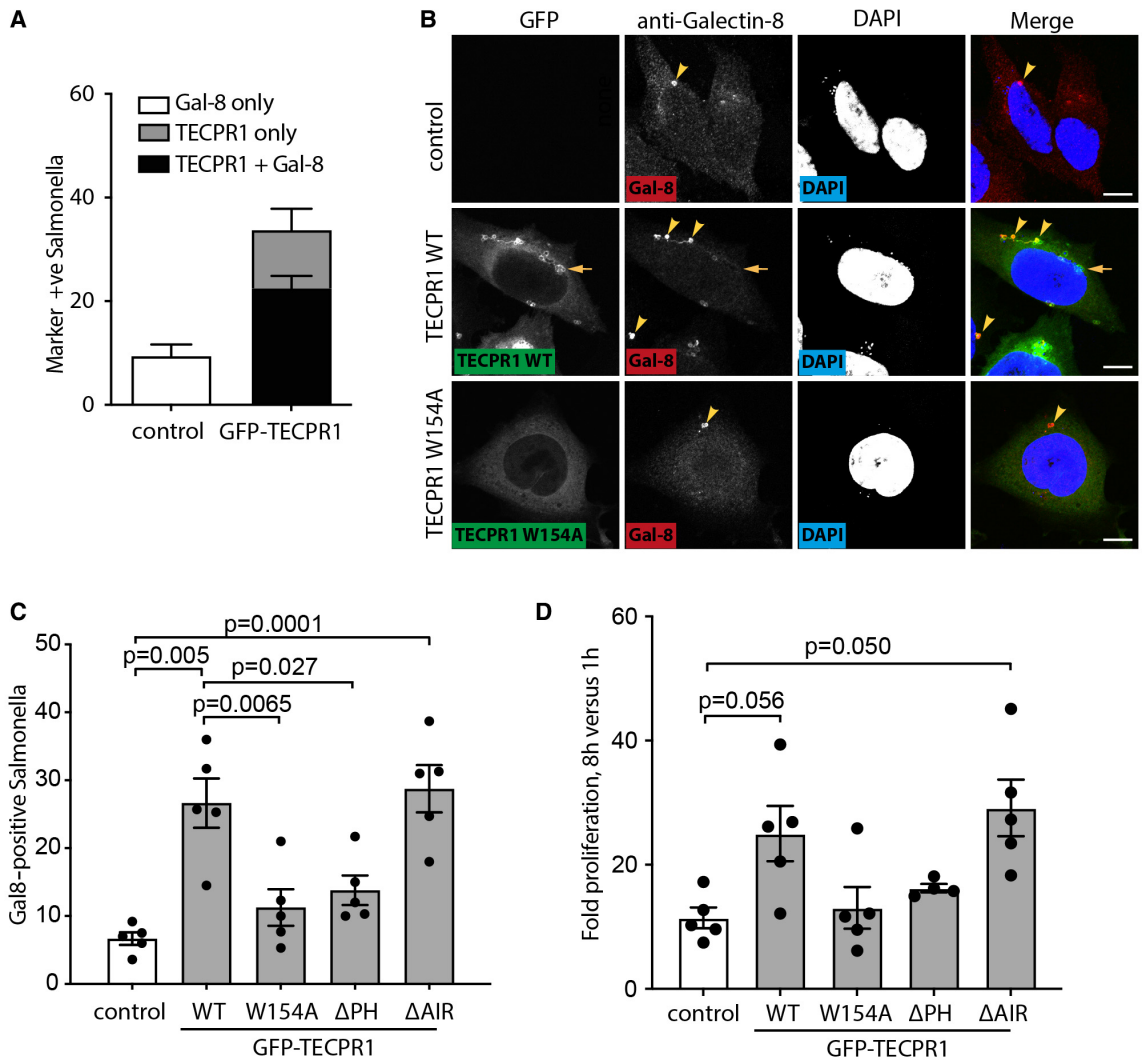
TECPR1 can damage sphingomyelin‐positive membranes APercentage of *S*. Typhimurium positive for Galectin‐8 and/or GFP‐TECPR1 in either control Hela cells or those expressing GFP‐TECPR1 at 1 h postinfection. Mean ± SEM of three independent experiments performed in duplicate. *n* > 100 bacteria per coverslip.BConfocal micrographs of HeLa cells expressing the indicated GFP‐TECPR1 constructs or not (control), infected with *S*. Typhimurium for 30 min, fixed and stained with anti‐Galectin‐8 and DAPI. Scale bar, 20 μm. Arrowheads indicate bacteria positive for Galectin 8, of which more are present in cells expressing GFP‐TECPR1 WT but not W154A. Arrow indicates bacterium to which GFP‐TECPR1 but not Galectin 8 has been recruited.CPercentage of *S*. Typhimurium positive for Galectin‐8 in Hela cells expressing the indicated GFP‐TECPR1 constructs or not (control) and infected with *S*. Typhimurium for 1 h. Mean ± SEM of five independent experiments performed in triplicate. *P*‐value from one‐way ANOVA with Tukey's multiple comparison test.DFold intracellular proliferation of *S*. Typhimurium in HeLa cells expressing the indicated GFP‐TECPR1 constructs or not (control) as assessed by colony forming unit assay. Mean ± SEM of four (ΔPH) or five (control, WT, W154A and ΔAIR) independent experiments. *P*‐value from one‐way ANOVA with Dunnett's multiple comparison test. Source data are available online for this figure.

About 10% of intracellular *S*. Typhimurium invade the host cytosol under control conditions, as indicated by the recruitment of Galectin‐8 to SCVs that had undergone catastrophic membrane damage (Figs 1H and 6A). By contrast, overexpressed GFP‐TECPR1 was recruited to 30–40% of SCVs, the majority of which also were Galectin‐8 positive, revealing that TECPR1 enhances recruitment of Galectin‐8 to SCVs, presumably due to increased damage to the SCV membrane (Figs 1H and 6A). To dissect how TECPR1 triggers enhanced Galectin‐8 recruitment, we tested several TECPR1 alleles. We found that TECPR1 W154A and TECPR1 ΔPH but not TECPR1 ΔAIR were impaired in potentiating recruitment of Galectin‐8 above control levels (Fig 6B and C). Any membrane‐rupturing effect of TECPR1 on SCVs is expected to yield an increase in cytosolic entry and ensuing proliferation of *S*. Typhimurium and, indeed, overexpression of membrane‐damaging TECPR1 alleles (WT and ΔAIR) but not membrane‐inert alleles (W154A and ΔPH) resulted in higher bacterial burden (Fig 6D). We conclude that when present at high levels TECPR1 accentuates damage to the SCV in a sphingomyelin‐dependent manner. We furthermore speculate that under appropriate circumstances, such as high local levels of sphingomyelin or increased protein expression, the endogenous protein may exhibit similar effects.

## Discussion

The exposure of sphingomyelin on the cytosolic leaflet of stressed membranes may alert cells to imminent membrane breakage (Ellison *et al*, 2020). However, the identity of the receptor detecting such cytosolically exposed sphingomyelin had remained unknown. Here, we report that the N‐terminal DysF domain of TECPR1 binds sphingomyelin directly, resulting in the recruitment of TECPR1 together with its binding partner ATG5 to sphingomyelin‐positive membranes and subsequent ATG16L1‐independent conjugation of LC3/GABARAP. We therefore propose that the cytosolic sphingomyelin receptor TECPR1, in complex with ATG5 and presumably ATG12, forms an ATG16L1‐independent E3 ligase for the conjugation of LC3/GABARAP to sphingomyelin‐positive membranes. Our findings are in accordance with an accompanying report examining the role of TECPR1 in the conjugation of LC3 to endolysosomal membranes upon perturbation by a variety of “sterile” agents (Kaur *et al*, 2023).

Mammalian cells have evolved multiple, often overlapping, lines of defence against attempted colonisation of their cytosol by intracellular pathogens (Randow *et al*, 2013; Fredlund & Enninga, 2014). Such multi‐layered systems permit the recognition of phyologenetically diverse pathogens while also providing cells with a certain resilience against attempts by pathogens to subvert individual defence pathways. The work described here reports cytosolically exposed sphingomyelin and TECPR1 as a novel DAMP ligand/danger receptor pair and positions it among the events known to occur when selective autophagy is deployed against damaged endomembranes. Conceptually, the detection of cytosolically exposed sphingomyelin by TECPR1 appears strikingly similar to the ATG16L1‐dependent detection of a perturbed vacuolar ATPase in the membrane of bacteria‐containing vacuoles since both TECPR1 and ATG16L1 act as receptor subunits of the ATG5‐ATG12 LC3 ligase complex to detect relatively minor perturbations in the homeostasis of phagosomes (Lau *et al*, 2019; Xu *et al*, 2019; Ulferts *et al*, 2021; Hooper *et al*, 2022) By contrast, the galectin‐8/NDP52–initiated autophagy pathway responds to glycan exposure on catastrophically ruptured membranes (Thurston *et al*, 2009, 2012; Ravenhill *et al*, 2019).

We discovered that the N‐terminal DysF domain of TECPR1 interacts with sphingomyelin, solved its crystal structure and mapped the binding site. So far, no function has been ascribed to any other mammalian DysF domain, despite considerable interest in Ferlins, the major family of DysF domain‐containing proteins. In particular, defects in the Dysferlin gene, including missense mutations in its DysF domain, cause various forms of muscular dystrophy due to shortcomings in membrane repair (Bulankina & Thoms, 2020; Ivanova *et al*, 2022). The structure of the TECPR1 N‐terminal DysF domain bears marked overall similarity to the DysF domains of Dysferlin and Myoferlin (Patel *et al*, 2008; Sula *et al*, 2014a). However, neither of these domains, nor the DysF domain from Fer1L5 or the C‐terminal DysF of TECPR1, are recruited to the membranes of damaged endosomes or bacteria‐containing vacuoles. Differences in residues required for the accumulation of the N‐terminal DysF domain of TECPR1 on sphingomyelin‐positive membranes explain the lack of recruitment of other DysF domains, although it is interesting to note that certain mutations in the Dysferlin DysF domain (M968L, R1022Q, P1029L) are surface exposed, therefore unlikely to cause structural instability and hence could mediate binding to an unknown ligand in the context of membrane repair (Data ref: Sula *et al*, 2014b).

Mutations in TECPR2 cause hereditary spastic paraplegia type 49 (SPG49), a neurodegenerative disorder of intellectual disability, chronic respiratory disease and decreased pain sensitivity (Heimer *et al*, 2016). TECPR2 encodes a C‐terminal LIR motif, indicating that TECPR2, just like TECPR1, serves autophagy‐related functions (Stadel *et al*, 2015; Heimer *et al*, 2016). However, the TECPR repeats of TECPR2 do not carry DysF domain insertions nor has any interaction with ATG5 been reported, suggesting that TECPR1 and TECPR2 perform quite distinct functions.

Both macroautophagy and various forms of noncanonical autophagy rely on the conjugation of LC3/GABARAPs to target membranes (Nieto‐Torres *et al*, 2021). While considerable redundancy exists in the precise mechanisms by which LC3 is recruited to vacuoles containing *S*. Typhimurium it was generally assumed that the process was entirely dependent on ATG16L1. Thus, our discovery of an ATG16L1‐independent mode of LC3 recruitment, by means of LC3‐conjugation through the newly identified TECPR1‐containing ATG5/ATG12 E3 ligase, challenges the ATG16L1‐centric view of LC3 conjugation and may guide future experiments as to why deficiency of ATG5 causes a stronger phenotype than defects in ATG16L1. Failure to recruit ATG5 to damaged Salmonella‐containing vacuoles suggests that TECPR1 facilitates LC3 lipidation in the immediate vicinity of the sphingomyelin‐positive structure. However, the defect in recruiting ATG5 to bacteria‐containing vacuoles in TECPR1^−/−^ cells does not cause a corresponding defect in LC3 recruitment, suggesting that LC3 lipidation by the ATG16L1/ATG5/ATG12 E3 ligase at locations distant from the damaged vacuole and subsequent membrane traffic perhaps compensates for lack of ATG5 recruitment in TECPR1−/− cells.

TECPR1 has previously been implicated in the recognition of *Shigella flexneri* Δ*icsB*, an autophagy‐sensitive strain, which was proposed to recruit TECPR1 through interactions with ATG5 and WIPI‐2, presumably on forming phagophores (Ogawa *et al*, 2011). However, such a model does not fit our data since recruitment of TECPR1 to vacuoles containing *S*. Typhimurium or *S. flexneri* was unaffected by either deletion of ATG5 or prevention of WIPI‐2 recruitment through PI3‐kinase inhibition. Rather, we propose TECPR1 as a sensor of sphingomyelin exposure on damaged membranes that is agnostic to the identity of the insult causing membrane damage. It thus will be important to characterise the contribution of TECPR1 in the cellular response to pathogens and other membranolytic agents, including those that may facilitate transbilayer movement of sphingomyelin but do not necessarily result in catastrophic membrane breakdown.

Most intracellular *S*. Typhimurium inhabit membrane‐surrounded vacuoles and only a minority colonises the cytosol, even though the majority of invading bacteria express the SPI‐1 Type III secretion apparatus responsible for vacuole rupture. A fine balance therefore seems to exist between the need to inject bacterial effector proteins into the host cell and alerting the cell to the presence of a pathogen inside the phagosome through the exposure of sphingomyelin. It is in this context that we made the surprising observation that the overexpression of TECPR1 exacerbated damage to the *Salmonella*‐containing vacuole. Binding of TECPR1 to sphingomyelin on the SCV was required to exacerbate membrane damage, as was the TECPR1 PH domain. Initial studies on TECPR1 suggested that its PH domain binds PI(3)P, at least in the context of immobilised “lipid strips” (Chen *et al*, 2012), while more recently the PH domain was found to bind specifically PI(4)P on giant unilamellar vesicles (Wetzel *et al*, 2020). Consistent with this scenario, *Salmonella*‐containing vacuoles, in contrast to other vesicles, accumulate PI(4)P (Domingues *et al*, 2016). It is thus tempting to speculate that the membrane‐damaging effect of TECPR1 requires coincident detection of PI(4)P and sphingomyelin, indicative of pathogen occupancy and membrane stress, respectively. Whether endogenous TECPR1 can cause membrane damage remains unclear, however, since depletion of TECPR1 did not affect the frequency with which *S*. Typhimurium accessed the cytosol. We anticipate any membrane‐damaging function of TECPR1 to be separate from its proposed role in autophagosomal maturation and lysosomal fusion (Chen *et al*, 2012; Wetzel *et al*, 2020). Recent evidence has suggested that cells may, in fact, harbour a sphingomyelin‐dependent membrane repair pathway (Niekamp *et al*, 2022). In this context, it also remains to be seen whether TECPR1 fosters membrane damage under specific circumstances, for example in dendritic cells during indirect antigen presentation where extracellular antigen was proposed to exit specifically from sphingomyelin‐positive vesicles into the cytosol (Canton *et al*, 2021).

## Materials and Methods

### Cell lines

HeLa (RRID:CVCL_0030), 293ET (RRID:CVCL_6996) and MEF cells were grown in Iscove's Modified Dulbecco's Medium (IMDM) with 10% foetal calf serum and gentamicin (30 μg/ml) at 37°C, 5% CO2. Stable cell lines were generated by retroviral transduction and antibiotic selection. All cell lines were routinely tested to be free of mycoplasma. TECPR1^+/+^ and TECPR1^−/−^ MEF cells were provided by Chihiro Sasakawa (Nippon Institute for Biological Science, Tokyo, Japan). ATG5^−/−^ MEF cells were provided by Noboru Mizushima (University of Tokyo, Tokyo, Japan).

### Bacteria

*S*. Typhimurium strains 12023 and 12023 Δ*prgH* + *inv* (encoded on pRI203, gifts from David Holden, Imperial College, London) were grown at 37°C on LB agar plates or in Luria Broth (LB). *S*. Typhimurium strain 12023 Δ*prgH* + *inv* lacks a functional SPI1‐T3SS and expresses the invasin (*inv*) gene of *Yersinia pseudotuberculosis* [31, 32]. This strain is referred to as Δ*inv* throughout this publication. *S*. Typhimurium 12023 strains either not expressing a fluorescent protein or expressing mCherry or BFP fluorescent proteins from a pFPV25.1 plasmid were used. Strains harbouring plasmids were grown in LB with 100 μg/ml ampicillin. *S*. *flexneri* strain M90T (gift from Chris Tang, Sir William Dunn School of Pathology, Oxford) was grown at 37°C on TSB agar plates containing 0.003% congo red or in Tryptic Soy Broth (TSB). *Listeria monocytogenes* BUG600 strain was provided by Pascal Cossart (Institut Pasteur, Paris, France) and grown on brain‐heart agar or broth.

### Cloning and gene expression

Plasmids used in this study are listed in Table EV1. M6P plasmids were used to generate recombinant MLV for the expression of proteins in mammalian cells (Randow and Sale). Open reading frames encoding TECPR1, ATG5, neutral sphingomyelinase 2 (SMPD3), the nested repeat DysF domains of dysferlin and myoferlin were amplified by PCR from a human brain cDNA library. The nested repeat DysF domain of Fer1L5 was generated by gene synthesis (Integrated DNA Technologies). Lysenin from *Eisenia fetida* was described (Ellison *et al*, 2020). Truncation and point mutants were generated by PCR using primers described in Table EV2. M6P‐based plasmids were cotransfected with pMD2‐VSVG and pMD‐OGP retroviral packaging plasmids into 293ET cells with polyethylenamine and retrovirus‐containing supernatant collected 48 h later. To overcome relatively low expression of the TECPR1 N‐terminal DysF‐GFP constructs HeLa cells were transduced twice with M6P‐based virus several days apart.

The HIV1‐based lentiviral plasmid H6P‐SFFV‐GFP‐PGKprom‐Neo and H6P‐SFFV‐GFP‐PGKprom‐hygro described here were derived from pHRSIN‐CSGW (gift from Prof. Paul Lehner, Cambridge Institute of Therapeutic Immunology & Infectious Disease, University of Cambridge, UK). The SFFV promoter was excised and replaced by PCR‐generated fragments of the promoter, progressively shortened from the 5′ end to give promoter lengths of 500 (A), 300 (B), 200 (C), 100 (D) and 80 bp (E). Recombinant lentivirus was generated using pMD‐CMV‐dR8.91 and pCMV‐VSVG as packaging plasmids transfected into 293ET cells with polyethylenamine and lentivirus‐containing supernatant collected 48 h later.

A bicistronic pETM11‐based bacterial expression plasmid for the expression of biotinylated N'DysF protein was generated as follows: TECPR1 N'DysF domain was PCR amplified using primers Kei381 and Kei407, to include a NcoI and MBAS‐NotI sequences, respectively. The *birA* gene was amplified using primers Kei409 and Kei410 to include an N‐terminal ribosomal binding site (RBS) and BfuAI site, respectively. Both PCR products were assembled by PCR and introduced to pETM11 using NcoI and BfuAI restriction sites.

### CRISPR

Guide RNAs against murine TECPR1, ATG5 or ATG16L1 were designed using the online ChopChop tool. gRNAs were cloned into the BbsI sites of pLKV‐sgRNA EF1Alpha‐puro‐T2A‐BFP with a panel of 5–8 gRNAs initially tested per gene. MEF cells stably expressing Cas9 were generated using pHRSIN‐SFFVprom‐Cas9‐PKGprom‐hygro (gift from Prof. Paul Lehner, Cambridge Institute for Medical Research). Shortly after drug selection, so as to ensure high expression of Cas9, cells were transduced with pLKV‐sgRNA EF1Alpha‐puro‐T2A‐BFP harbouring relevant gRNAs at a high multiplicity of infection. Cells were expanded in puromycin‐containing medium and samples prepared for Western blotting on day 7 post‐transduction with gRNA virus. Those cells harbouring gRNAs giving the most efficient knockout of their target gene were selected for further experiments. TECPR1 gRNA‐expressing cells were evaluated for their ability to support recruitment of ATG5 to *S*. Typhimurium prior to confirmation of the most effective gRNA by Western blotting.

TECPR1‐deficient MEF cells, stably expressing Cas9 under puromycin resistance and TECPR1 gRNA under hygromycin resistance, were complemented with different H6P‐SFFV‐muTECPR1^gRNA resistant^‐PGKprom‐Neo derived lentiviruses containing each of the different SFFV promoters described above. gRNA resistant murine TECPR1 was amplified by assembly PCR of reactions Kei1030/1027 and 1026/1028 to mutate the gRNA site from AGG GTGGCCCATATAAGGTC to AG**C** GT**T**GCCCA**G**AT**C**A**A**GTC (PAM site underlined and mutations in bold).

### RNA interference

MEF cells were seeded in 24‐well format to approximate 20% confluency prior to transfection with 10 nM siRNA using Lipofectamine RNAiMAX. Medium was changed after 48 h and cells infected after further 24 h.

### Bacterial infections

*S*. Typhimurium strains 12023 and 12023 Δ*prgH* + *inv* were grown overnight at 37°C, 180 rpm in Luria Broth (LB) with the addition of 100 μg/ml ampicillin in the case of 12023 Δ*prgH* + *inv* or where a fluorescent protein is expressed. *S*. Typhimurium were subinoculated at a ratio of 1:33 into fresh LB, 3.5 h preinfection. HeLa and MEF cells grown in 24‐well format in the absence of antibiotics, either on glass coverslips or not, were infected with 20 μl of a 1:5 dilution of subculture per well for 15 min at 37°C. Cells were washed twice in warm phosphate‐buffered saline (PBS, pH 7.4) and cultured in IMDM/10% FCS/100 μg/ml gentamycin for 1 h, after which the medium was exchanged for IMDM/10%FCS/20 μg/ml gentamycin. Cells were treated with 100 nM wortmannin or DMSO vehicle control 15 min prior to infection.

*Salmonella flexneri* strain M90T was grown overnight at 37°C, 180 rpm in Tryptic Soy Broth (TSB) with the addition of 100 μg/ml ampicillin in the case of those strains where a fluorescent protein is expressed. *S. flexneri* were subinoculated at a ratio of 1:100 into fresh TSB, 2.5 h preinfection. HeLa and MEF cells grown in 24‐well format in the absence of antibiotics were infected with 100 μl of subculture and centrifuged for 10 min at 2,000 rpm, 20°C followed by incubation at 37°C for 30 min. Infection thereafter was as described for *S*. Typhimurium.

*Listeria monocytogenes* strain BUG600 was grown overnight at 30°C, 180 rpm in brain‐heart broth, subcultured at 1:33 dilution and grown for a further 3.5 h at 30°C, 180 rpm. HeLa cells grown in 24‐well format in the absence of antibiotics were infected with 5 μl of subculture and centrifuged for 10 min at 2,000 rpm, 20°C followed by incubation at 37°C for 30 min. Medium was exchanged for that containing 100 μg/ml gentamycin until desired time point.

### Enumeration of intracellular *S*. Typhimurium (colony forming unit assay)

At relevant time points postinfection with *S*. Typhimurium or *S. flexneri*, HeLa cells seeded in triplicate wells were lysed in 1 ml cold PBS containing 0.1% Triton X‐100, described in detail in (Boyle & Randow, 2019). Serial dilutions were plated on LB agar plates. Plates were incubated overnight at 37°C and colonies were counted using an aCOLyte3 colony counter (Symbiosis).

### Osmotic shock and lysosomal damage assay

Cells were cultured on glass coverslips for 48 h prior to assay. Medium on cells was replaced with hypertonic medium (0.5 M sucrose in PBS, with 10% (w/v) polyethyleneglycol 1,000) for 10 min at 37°C. Cells were then washed and incubated in 60% PBS for 3 min followed by incubation in complete IMDM medium for 20 min at 37°C. For lysosomal damage, cells were treated with either 250 μM LLOMe or DMSO vehicle control for 10 min at 37°C.

### Microscopy

At designated time points cells were fixed in PBS/4% paraformaldehyde for 15 min at room temperature. PFA‐fixed samples were washed twice with PBS and quenched in PBS/100 mM glycine. Samples were permeabilised in PBS/0.2% Triton X00 for 5 min, washed and blocked in PBS/2% BSA for 30 min. Samples were stained with primary antibody (1:50 for anti‐Galectin‐8 [AF1305 R&D Systems], 1:200 for anti‐WIPI2 [ab105459, Abcam], 1:200 anti‐ATG5 [A0731, Sigma]) for 1 h, rinsed in PBS, stained with relevant Alexa‐conjugated secondary antibody for 30 min, rinsed again in PBS followed by water and mounted either in DAPI mounting medium (Vector Laboratories) or ProLong Gold Antifade Mountant (Invitrogen). For staining with anti‐LC3 (CTB‐LC3‐2‐IC, Cosmo Biosciences), PFA‐fixed cells were permeabilised in PBS/2% BSA/0.05% saponin for 30 min, incubated with anti‐LC3 primary (1:50) in PBS/2% BSA/0.05% saponin for 1 h followed by relevant Alexa‐conjugated secondary in PBS/2% BSA/0.05% saponin for 30 min. Marker‐positive bacteria were enumerated by eye among at least 100 bacteria per coverslip using a wide‐field microscope, with coverslips blinded where indicated in Figure Legends. Confocal images were taken with a 63x, 1.4 numerical aperture objective on either a Zeiss 710 or a Zeiss 780 microscope. Live‐cell imaging was achieved using a 60x, water objective of Nikon Eclipse Ti equipped with an Andor Revolution XD system and a Yokogawa CSU‐X1 spinning disk unit. Movies were analysed using Imaris software version 8.

### Western blotting

Cells grown in 6‐well format were washed in ice‐cold PBS and scraped in 100 μl of Lysis Buffer (20 mM HEPES pH 7.4, 150 mM NaCl, 1% Triton X‐100, 5 mM EDTA and protease inhibitors) at the relevant time. Protein concentration was estimated using 660 nm Protein Assay Reagent (Pierce) and 20–30 μg of protein loaded on SDS–PAGE gel. Antibodies used were anti‐TECPR1 (8097, Cell Signalling), anti‐ATG5 (A0731, Sigma), anti‐ATG16L1 (8089, Cell Signalling), anti‐GFP (JL8, Clontech) and anti‐β Actin (ab8227, Abcam).

### Cell surface binding

HeLa cells were trypsinised and washed twice in 20 mM HEPES (pH 7.4), 150 mM NaCl. Cells were incubated at room temperature with 400 nM DysF^Strep546^ WT or DysF^Strep546^ W154A, 200 nM GFP‐conjugated Lysenin C‐terminal domain (161–297) WT or K185A, in the presence or absence of a 10‐fold molar excess (4 μM) of Lysenin^CTD WT^‐GFP or Lysenin^CTD K185A^‐GFP. Cells were washed twice in 20 mM HEPES (pH 7.4), 150 mM NaCl, once in PBS/1% BSA and resuspended in PBS/1% BSA before analysis on a Becton Dickinson Fortessa flow cytometer. Data were analysed on FlowJo software.

### Protein purification

TECPR1 N‐terminal DysF WT (60–173 aa) and W154A were expressed from pETM11 vector in BL21 *E. coli*. Cells were grown at 37°C in 2xTY supplemented with 30 μg/ml kanamycin to OD600 value of 0.7. Cultures were induced with IPTG (100 μM) at 18°C and harvested after 16 h. Proteins were purified by immobilised Nickel metal‐affinity chromatography using 5 ml HisTrap column. Imidazole‐eluted protein was incubated with TEV protease overnight at 4°C to cleave the 6His tag, followed by incubation with Nickel resin again and collection of DysF protein from the flow‐through. DysF proteins were further purified by size exclusion chromatography using a Superdex 75 16/600 column (GE Healthcare) in 20 mM Tris–pH 7.4, 150 mM NaCl, 2 mM DTT. For the generation of streptavidin‐conjugated DysF protein (DysF^Strep546^) a minimal biotin acceptor site was cloned at the C‐terminus of DysF and the protein expressed together with *birA* in BL21 *E. coli* in the presence of 20 μM biotin. The protein was purified as above, incubated with AlexaFluor‐546‐conjugated streptavidin (1:1 weight/weight; Thermo Fisher Scientific) at 4°C for 3 h and further purified by size exclusion chromatography using a Superdex 75 16/600 column (GE Healthcare) in 20 mM Tris–pH 7.4, 150 mM NaCl, 2 mM DTT. Lysenin^CTD WT^‐GFP and Lysenin^CTD K185A^‐GFP proteins were generated as described previously (Ellison *et al*, 2020).

TECPR1 WT, ΔN‐terminal DysF (Δ65‐170‐ > GSGG) and W154A were cloned into pGB‐05‐06‐ccdB vector (LMBP accession 12,029; Belgian Coordinated Collection of Microorganisms) including a GST tag and a TEV cleavage site. The proteins were expressed by means of a baculovirus expression system using Sf9 cells. For purification, pellets were thawed in 50 mM Tris–HCl (pH 8.0), 300 mM NaCl, 10% glycerol, 2 mM MgCl2, 1 mM β‐mercaptoethanol, complete protease inhibitors (Roche), Protease Inhibitor Cocktail (Sigma) and Benzonase Nuclease (Sigma). Cells were lysed on ice by extrusion in a tissue homogeniser, and lysates were cleared by centrifugation at 20,000 rpm for 45 min at 4°C. Supernatant was incubated with Glutathione sepharose beads (Cytiva) for 1 h at 4°C. Beads were washed in 50 mM Tris–HCl (pH 8.0), 300 mM NaCl and 1 mM DTT; the protein was eluted by adding 50 mM glutathione; and the GST tag was cleaved off using TEV protease. The protein was applied onto a Superdex 200 Increase 10/300 GL column (Cytiva) and eluted in 25 mM Tris–HCl (pH 8.0), 200 mM NaCl and 1 mM DTT. Fractions containing the final protein were pooled, concentrated, snap‐frozen in liquid nitrogen and stored at −70°C. ATG7, ATG3, ATG12‐5 and LC3B were expressed and purified as previously described (Fracchiolla *et al*, 2020).

### Reductive methylation of DysF protein

Recombinant DysF protein was buffer exchanged for 50 mM Hepes pH 7.4, 250 mM NaCl, 20 μl of 1 M borane‐dimethylamine complex per mg of protein added followed by 40 μl of 1 M formaldehyde and incubated for 2 h at 4°C. A further 10 μl 1 M borane‐dimethylamine complex was added, overnight at 4°C. Protein was immediately applied to a Superdex 75 column for size exclusion chromatography in 20 mM Tris–pH 7.4, 200 mM NaCl, concentrated to 8 mg/ml and seeded in crystal trays. Methylated protein was analysed by MALDI mass spectrometry and electrospray mass spectroscopy.

### Crystallisation and structure determination

Crystals of methylated DysF protein were grown by sitting‐drop vapour diffusion in conditions containing 25% PEG6000, 0.1 M Sodium HEPES pH 7.5, 0.1 M LiCl and were transferred to reservoir solution containing 25% (v/v) glycerol prior to cryo‐cooling. Diffraction data were collected at Diamond Light Source beamline I04‐1. Diffraction images were processed using XDS (Kabsch, 2010) and scaled using AIMLESS (Evans & Murshudov, 2013). The structure of the DysF domain was solved by molecular replacement using PHASER (McCoy *et al*, 2007) placing three copies of the myoferlin DysF domain (PDB ID: 2K2O). Iterative rounds of model building and refinement were performed with COOT (Emsley *et al*, 2010) and PHENIX (Adams *et al*, 2010), respectively. Data collection and refinement statistics can be found in Table 1. The PDB accession code for TECPR1 N‐terminal DysF domain is 8P5P.

### Liposome flotation assays

Sphingomyelin from egg yolk, phosphatidylcholine (PC) from porcine brain and cholesterol (Chol) were obtained from Avanti Polar Lipids. Lipids were mixed in chloroform at the following ratios: PC and Chol (60:40); sphingomyelin, PC and Chol (50:10:40). Solvent was evaporated under nitrogen flow and lipids were further dried for 1 h under a vacuum. Lipid mixtures were rehydrated in rehydration buffer (50 mM HEPES, 100 mM KoAC, 1 mM DTT, 10% OptiPrep [SIGMA]) and added to a final lipid concentration of 1 mg/ml for 1 h. Liposomes were prepared with a Mini‐Extruder (Avanti Polar Lipids, Inc.) using Nucleopore track‐etched membranes with 400 nm followed by 100 nm pores (Whatman). Liposomes were validated by dynamic light scattering using a DynaPro Plate Reader II instrument (Wyatt Technology). Liposomes containing 10% OptiPrep were incubated with recombinant proteins at 20 μg/ml or cell lysates for 1 h at r.t. OptiPrep was then added to a final concentration of 30% and the mixture was overlaid with a 10% OptiPrep layer and a 0% Optiprep layer (rehydration buffer only). Liposomes were floated in a SW60Ti swinging bucket rotor (51,000 rpm, 30 min, 4°C). Liposomes floating at the 10–0% Optiprep interface were collected and washed by relayering of the OptiPrep gradient and refloating. Floating liposomes were collected and bound proteins were precipitated via methanol:chloroform extraction.

For liposome sedimentation assays liposomes were generated at the following ratios: PC and Chol (60:40); sphingomyelin, PC and Chol (5:55:40). Forty microlitre of liposomes at a concentration of 1 mM was incubated with the indicated proteins (TECPR1 full‐length at 1 μM, Lysenin^CTD^‐GFP at 2 μM or TECPR1 N‐terminal DysF WT or W154A at 5 μM; all proteins were precleared by centrifugation at 66,000 rpm in a TLA100 rotor) in a final volume of 50 μl for 30 min at room temperature in ultracentrifuge tubes (Becton Coulter, 343775), ultracentrifuged at 66,000 rpm in a TLA 100 rotor (Becton Coulter) for 30 min, the supernatant removed and both the pellet and supernatant fractions resuspended in an equal volume of Laemmli loading buffer. Equal volumes of fractions were loaded on SDS–PAGE gels and proteins detected with Instant Blue (Expedeon). Quantification of protein bands was performed with ImageLab (BioRad Laboratories) software.

### LC3B conjugation assay

Lipids used for liposome preparation were from Avanti Polar Lipids: POPC (850457C, 10 mg/ml), POPS (840034C, 10 mg/ml), POPE (850757C, 10 mg/ml), PI(3)P (850150P, 1 mg/ml) and SM (860061C, 10 mg/ml). Liposomes with and without sphingomyelin (SM) were prepared as described (Fracchiolla *et al*, 2020) in 25 mM HEPES, 150 mM NaCl and 0.1 mM DTT and the following lipid compositions: 40/60% POPC, 15% POPS, 20% POPE, 5% PI(3)P and 20/0% SM. The final concentration was 1 mM. Forty microlitre of SUVs was mixed with 1 μM of TECPR1 wt/Δ65‐179/W154A, ATG7, ATG3, ATG12‐5 and 5 μM of LC3B in the presence of 1 mM MgCl_2_ and 5 mM ATP in a final volume of 50 μl, and incubated for 3 h at 37°C. As controls, mixtures without ATG3 or ATP were prepared. Samples were pelleted at 68,000 rpm for 15 min using a Beckman ultracentrifuge with a TLA100.1 rotor. Supernatant and pellet fractions were run on 4–15% SDS–polyacrylamide gels (BioRad) and stained with Coomassie Brilliant Blue.

### Lysate preparation for liposome flotation and mass spectrometry

Approximately 2 × 10^8^ HeLa or HCT116 cells were trypsinised, treated with 7 μg/ml bacterial sphingomyelinase (bSMase) for 30 min and washed five times with PBS (1,500 rpm, 5 min, 4°C). Pellet was resuspended in Resuspension Buffer (20 mM Tris–pH 7.4, 1 mM DTT, 1 μg/ml aproptinin, 1 mM benzamidine, 5 μg/ml leupeptin and 1 mM PMSF) and subjected to five rounds of freeze–thaw in liquid nitrogen. Cytosolic fraction was collected after centrifugation at 13,000 rpm, 15 min, 4°C followed by 45,000 rpm, 45 min, 4°C. Supernatant was diluted to 3.5 ml volume with Resuspension Buffer for binding assay.

### TMT labelling and mass spectrometry

Protein pellets were resuspended in 8 M urea, 50 mM ammonium bicarbonate, 10 mM DTT at 56°C for 30 min followed by the addition of 200 mM iodoacetamide for 30 min at 22°C in the dark. The reaction was quenched by the addition of 100 mM DTT for 10 min at 22°C and samples digested with 0.25 mg/ml trypsin (Promega) in 50 mM ammonium bicarbonate overnight at 37°C. Digestion was terminated with trifluoroacetic acid (TFA); the peptide mixtures desalted using poros R3 resin (Applied Biosystem); and bound peptides eluted with 60% acetonitrile (MeCN) in 0.1% TFA and lyophilised. Peptides were resuspended in 5% MeCN and peptide concentrations determined by Pierce Quantitative Colorimetric Peptide assay (Thermo Scientific) according to manufacturer instructions. TMT duplex reagents (Thermo Fisher Scientific) were reconstituted to 19.5 mg/ml in MeCN. Twenty microlitre of TMT duplex reagent was added to each peptide mixture in 170 mM triethylammonium bicarbonate (TEAB) and incubated for 1.5 h at 22°C. Labelling reactions were terminated with 5% hydroxylamine for 15 min and samples were pooled followed by MeCN removal by speed vacuum and were desalted. Eluted peptides were partially dried down in a speed vacuum and prepared for LC‐MSMS.

The labelled peptides were analysed on a Q Exactive Plus hybrid quadrupole‐Orbitrap mass spectrometer (Thermo Fisher Scientific). Liquid chromatography was performed on a fully automated Ultimate 3000 RSLC nano System (Thermo Scientific) fitted with a 100 μm × 2 cm PepMap100 C18 nano trap column and a 75 μm × 25 cm reverse phase C18 nano column (Aclaim PepMap, Thermo Scientific). Samples were separated using a binary gradient consisting of buffer A (2% MeCN, 0.1% formic acid) and buffer B (80% MeCN, 0.1% formic acid). Peptides were eluted with a linear gradient from 4 to 50% buffer B over 90–108 min with a flow rate of 300 nl/min. The HPLC system was coupled to a Q Exactive Plus mass spectrometer (Thermo Scientific) equipped with a nanospray ion source. The mass spectrometer was operated in standard data‐dependent mode and performed a survey full‐scan (MS, *m*/*z* = 350–1,600) with a resolution of 140,000, followed by MS2 acquisitions of the 15 most intense ions with a resolution of 35,000 and NCE of 32%. MS target values of 3 × 106 and MS2 target values of 1 × 105 were used. Dynamic exclusion was enabled for 40 s.

The acquired MSMS raw files were processed using Proteome Discoverer (version 2.1, Thermo Scientific). MSMS spectra were searched against Uniprot human 2016 database using Mascot (version 2.4, Matrix Science). The abundance values of the TMT reporter ion were scaled to the control channel and normalised to total peptide amount. Only high confident peptides with false discovery rate (FDR) of 1% were included in the results.

## Author contributions

**Felix Randow:** Conceptualization; supervision; funding acquisition; writing – original draft; project administration; writing – review and editing. **Keith B Boyle:** Conceptualization; investigation; methodology; writing – original draft; writing – review and editing. **Cara J Ellison:** Investigation. **Paul R Elliott:** Investigation. **Martina Schuschnig:** Investigation. **Krista Grimes:** Investigation. **Marc S Dionne:** Supervision; investigation. **Chihiro Sasakawa:** Resources. **Sean Munro:** Conceptualization; supervision. **Sascha Martens:** Supervision; investigation.

## Disclosure and competing interests statement

Sascha Martens is a member of the Scientific Advisory Board of Casma Therapeutics.

## Supporting information



Expanded View Figures PDF

Table EV1

Table EV2

Movie EV1

PDF+

Source Data for Figure 1

Source Data for Figure 2

Source Data for Figure 3

Source Data for Figure 4

Source Data for Figure 5

Source Data for Figure 6

## Data Availability

TECPR1 N‐terminal DysF domain protein crystallographic structure: PBD identifier 8P5P (https://www.rcsb.org/structure//8P5P). Cells, nucleic acids and other reagents that were used in the research reported and that are not available from commercial suppliers will be made freely available to colleagues in academic research upon reasonable request.
