# Supplementary material for: TECPR1 conjugates LC3 to damaged endomembranes upon detection of sphingomyelin exposure
Source: EMBO J. 2023 Jul 6;42(17):e113012. doi: 10.15252/embj.2022113012 (PMC10476172; doi:10.15252/embj.2022113012)
Supplement: Supplementary file 1 — Expanded View Figures PDF [file EMBJ-42-e113012-s001.pdf]

Expanded View Figures

Figure EV1. (corresponding to Fig 1). TECPR1 detects sphingomyelin on the cytosolic face of bacteria-containing vacuoles.

A Histograms displaying the diameter of liposomes composed of phosphatidylcholine:cholesterol (60:40; top) or sphingomyelin:phosphatidylcholine: cholesterol (50:10:40; bottom), as measured by dynamic light scattering.

B Confocal micrograph of HeLa cells expressing GFP-TECPR1 infected with *S.* Typhimurium Δ*prgH* + *inv*, fixed at 1 h postinfection and stained with DAPI. Scale bar, 10 μm.

C Percentage of *S.* Typhimurium WT or Δ*prgH* + *inv* positive for GFP-TECPR1 in Hela cells fixed at 1 h postinfection. Mean ± SD of two independent experiments performed in duplicate. $n > 100$ bacteria per coverslip.

D ATG5-deficient mouse embryonic fibroblasts were retrovirally transduced with an AU1-tagged ATG5 construct or not, lysates prepared and analysed by SDS–PAGE and Western blotted with the indicated antibodies. Upper band in anti-ATG5 blot is conjugated ATG5-ATG12 while the lower is monomeric ATG5. PCNA serves as a loading control.

E Confocal micrographs of ATG5-deficient MEF cells, complemented with AU1-ATG5 or not and expressing mCherry-TECPR1, were infected with *S.* Typhimurium, fixed at 1 h postinfection and stained with DAPI to label bacteria. Scale bar, 20 μm.

F, G Confocal micrographs of HeLa cells expressing GFP-TECPR1 alone (G) or together with mCherry-tagged FYVE domain from WDFY1 (F) were pretreated with 100 nM Wortmannin or mock control (DMSO) infected with *S.* Typhimurium for 1 h, fixed and stained with anti-WIPI2 (G) and DAPI to label bacteria. Scale bar, 20 μm.

 © 2023 MRC Laboratory of Molecular Biology and The Authors

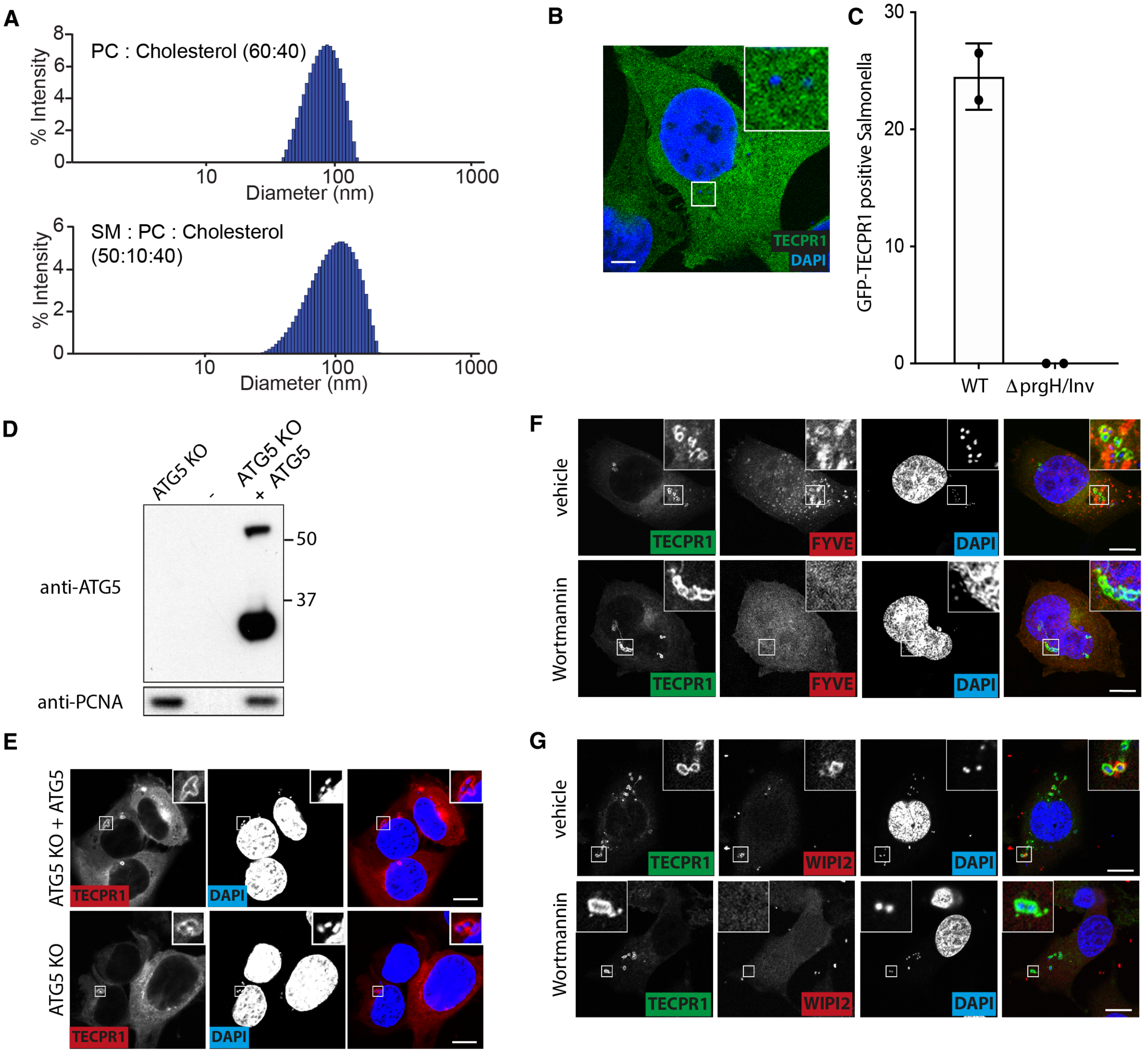
A
PC : Cholesterol (60:40)
% Intensity
Diameter (nm)
SM : PC : Cholesterol
(50:10:40)
B
TECPR1
DAPI
C
GFP-TECPR1 positive Salmonella
WT
ΔprgH/Inv
D
ATG5 KO
ATG5 KO
-
+ ATG5
anti-ATG5
anti-PCNA
50
37
E
ATG5 KO + ATG5
ATG5 KO
TECPR1
DAPI
F
vehicle
Wortmannin
TECPR1
FYVE
DAPI
G
WIPI2

Figure EV1.

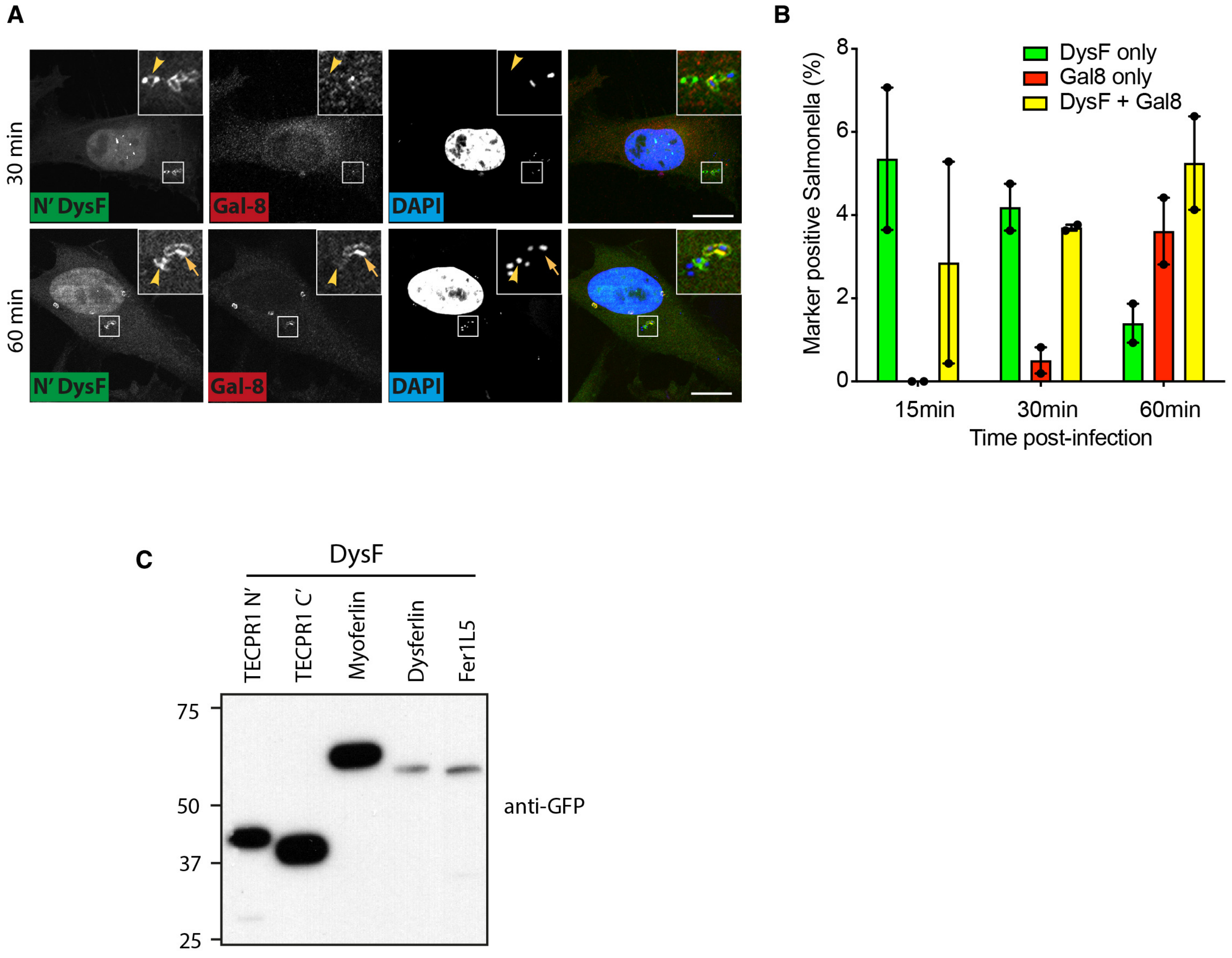

Figure EV2. (corresponding to Fig 2). The N-terminal DysF domain of TECPR1 detects sphingomyelin.

A Confocal micrographs of Hela cells expressing N-terminal DysF domain of TECPR1 (N'DysF) as a C-terminal GFP fusion were infected with *S.* Typhimurium, fixed at the indicated times postinfection, stained with anti-Galectin 8 and DAPI. Arrowhead indicates bacteria to which DysF-GFP but not Galectin 8 is recruited, whereas arrow indicates those to which both proteins are recruited. Scale bar, 20 μm.

B Percentage of *S.* Typhimurium positive for N-terminal DysF-GFP and/or anti-Galectin 8 in HeLa cells fixed at the indicated times postinfection. Mean ± SD of two independent experiments performed in duplicate.

C Hela cells stably expressing the indicated GFP-tagged DysF constructs were prepared for SDS–PAGE and Western blotted with anti-GFP antibody.

 © 2023 MRC Laboratory of Molecular Biology and The Authors

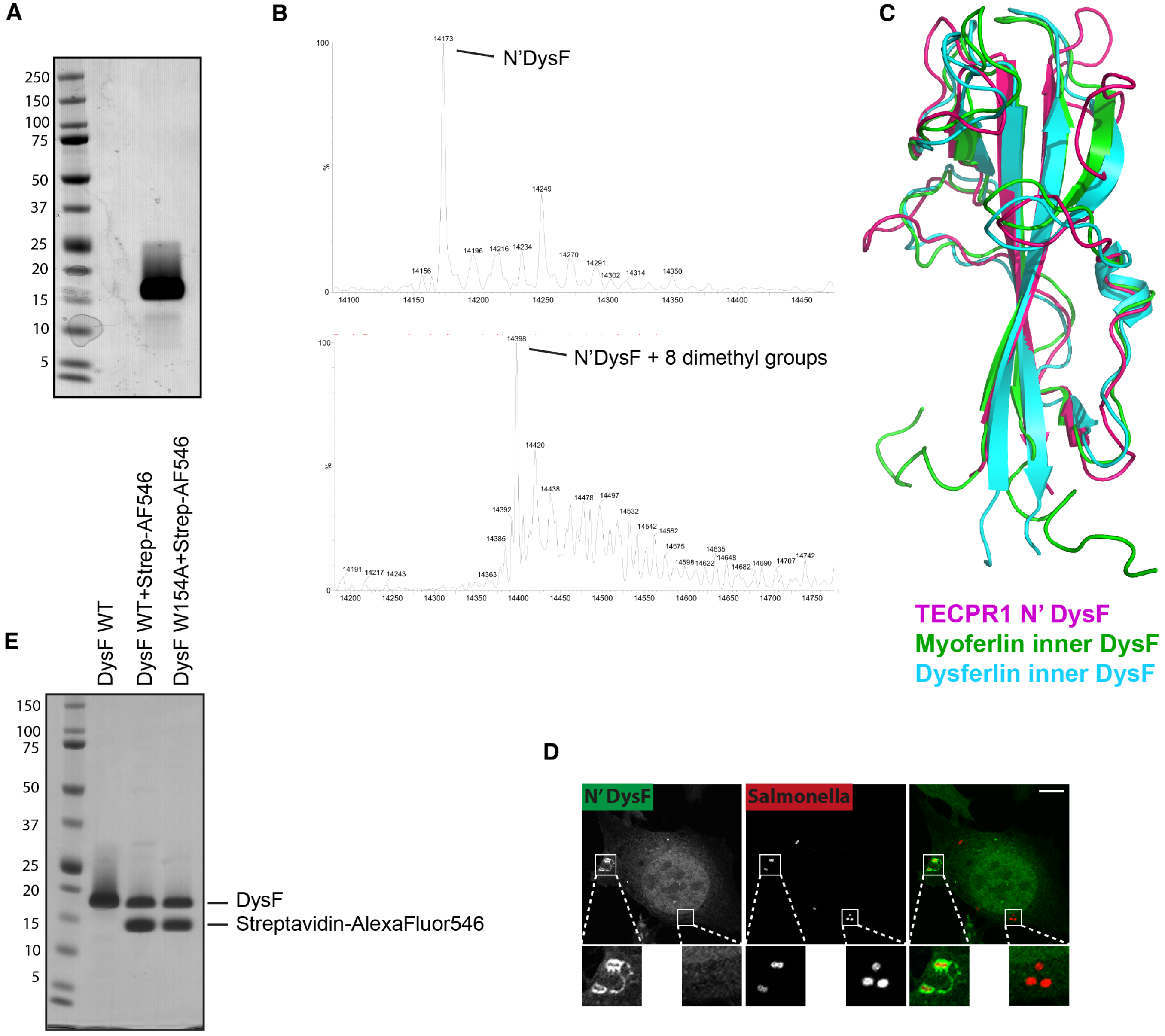

Figure EV3. (corresponding to Fig 3). Identification of the sphingomyelin-binding site in the N-terminal DysF domain.

- A Coomassie stain of SDS–PAGE gel of recombinant TECPR1 N-terminal DysF domain purified from *E. coli*.
- B Mass spectrometry of recombinant TECPR1 N-terminal DysF protein before (top) and after (bottom) reductive methylation. Note the shift in mass of major species from 14,173 to 14,398 Da (225 Da) corresponding to eight dimethyl groups attached to seven lysine and N-terminal methionine residues.
- C Overlay of the crystal structure of TECPR1 N-terminal DysF domain and that of the NMR structure of the inner DysF domain of myoferlin (PDB: 2K2O) and the crystal structure of the inner DysF domain of Dysferlin (PDB: 4CAH).
- D Confocal micrographs of Hela cells expressing N'DysF-GFP infected with mCherry-Salmonella for 30 min and fixed. Micrograph is same as that shown in Fig 2G. Insets depict SCVs that are scored as being either positive (left) or negative for DysF-GFP (right). Scale bar, 20 μm.
- E Coomassie stain of SDS–PAGE gels of recombinant N'DysF WT or W154A protein before and after labelling with streptavidin$^{\text{AlexaFluor46}}$.

© 2023 MRC Laboratory of Molecular Biology and The Authors

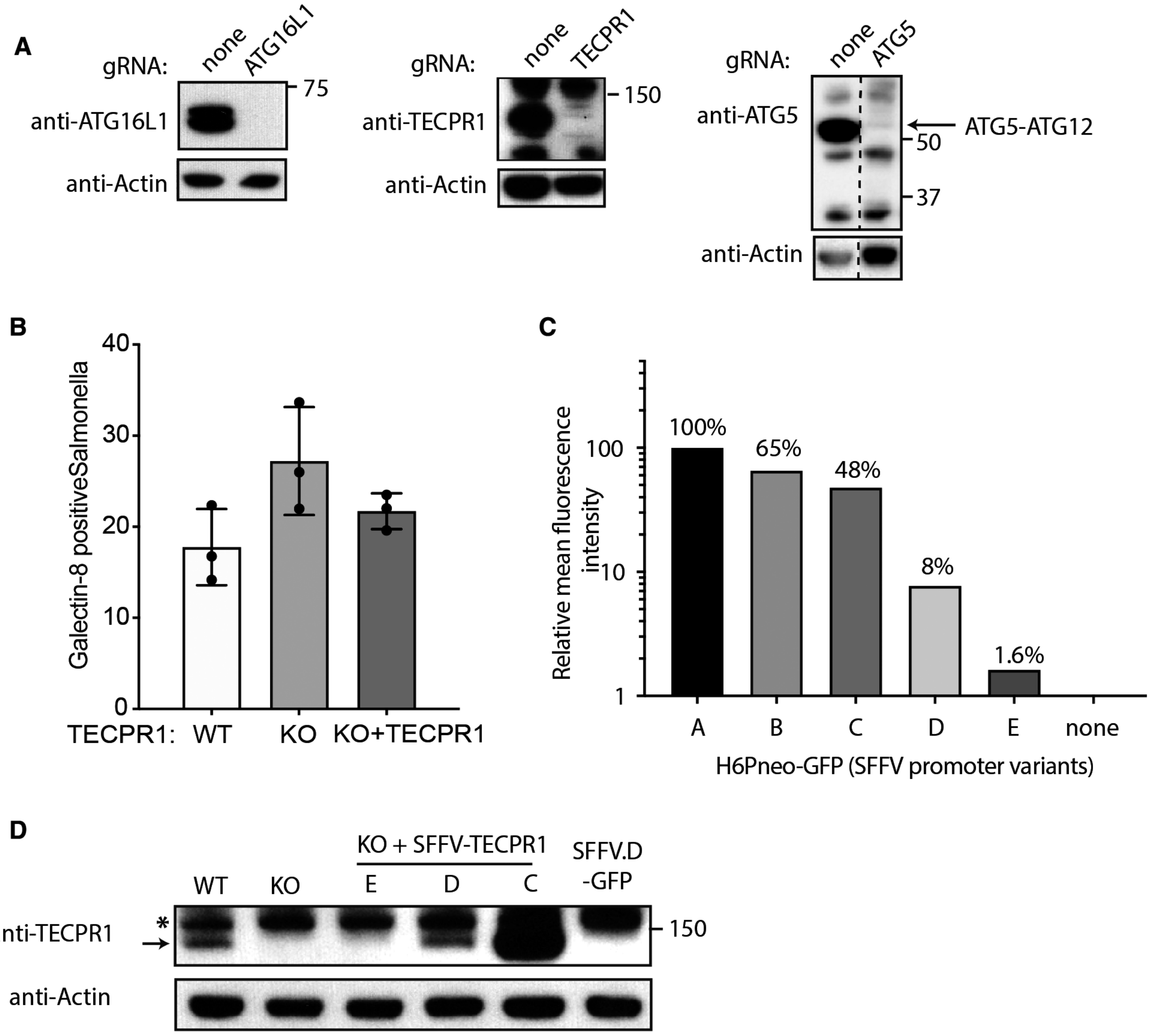

Figure EV4. (corresponding to Fig 5). TECPR1 recruits ATG5 to Salmonella-containing vacuoles for ATG16L1-independent LC3 conjugation.

A Western blot of lysates from MEF cells firstly lentivirally transduced to stably express Cas9 followed by stable lentiviral expression of indicated gRNAs for 7 days and blotted with antibodies shown. Hatched line in (F) denotes where intervening, irrelevant lanes of the blot were excised.

B Percentage of *S.* Typhimurium positive for anti-Galectin 8 in TECPR1 WT MEF cells, KO or KO complemented with TECPR1 construct fixed at 1 h postinfection. Mean + SEM of three independent experiments performed in duplicate. Statistical significance, based on a one-way ANOVA with the Tukey's multiple comparison test, was not reached between any of the groups.

C Relative expression level of GFP in HeLa cells stably transduced with H6Pneo lentivirus, selected with G418 and measured by flow cytometry. H6Pneo harbours GFP under the control of one of five different, progressively shorter portions of the spleen focus-forming virus (SFFV) promoter (A–E) as described in Materials and Methods.

D Western blot of TECPR1 WT or KO MEFs or KO complemented with TECPR1 or GFP control from indicated H6P-based lentiviruses harbouring SFFV promoters corresponding to those in (B). Arrow indicates specific band corresponding to TECPR1 at 135 kDa, and star denotes nonspecific band. Actin serves as loading control.

 © 2023 MRC Laboratory of Molecular Biology and The Authors
