# Supplementary material for: TECPR1 conjugates LC3 to damaged endomembranes upon detection of sphingomyelin exposure
Source: EMBO J. 2023 Jul 6;42(17):e113012. doi: 10.15252/embj.2022113012 (PMC10476172; doi:10.15252/embj.2022113012)
Supplement: Supplementary file 6 — Source Data for Figure 1 [file EMBJ-42-e113012-s005.zip › Figure 1/1D/1D README.rtf]

Original image corresponding to Figure 1D_top, Salmonella infection, could not be found.Figure 1D_middle is original uncropped image of Shigella infectionFigure 1D_bottom is original uncropped image of Listeria infection
