# Supplementary material for: TECPR1 conjugates LC3 to damaged endomembranes upon detection of sphingomyelin exposure
Source: EMBO J. 2023 Jul 6;42(17):e113012. doi: 10.15252/embj.2022113012 (PMC10476172; doi:10.15252/embj.2022113012)
Supplement: Supplementary file 6 — Source Data for Figure 1 [file EMBJ-42-e113012-s005.zip › Figure 1/1C/1C README.rtf]

Figure 1C_top is original uncropped image of control condition with DAPI channel includedFigure 1C_middle is original uncropped image of Osmotic shockFigure 1C_bottom is original uncropped image of LLOMe treatment with DAPI channel included, rotated by 270 degrees
