# Supplementary figures and images for: TECPR1 conjugates LC3 to damaged endomembranes upon detection of sphingomyelin exposure
Source: EMBO J. 2023 Jul 6;42(17):e113012. doi: 10.15252/embj.2022113012 (PMC10476172; doi:10.15252/embj.2022113012)

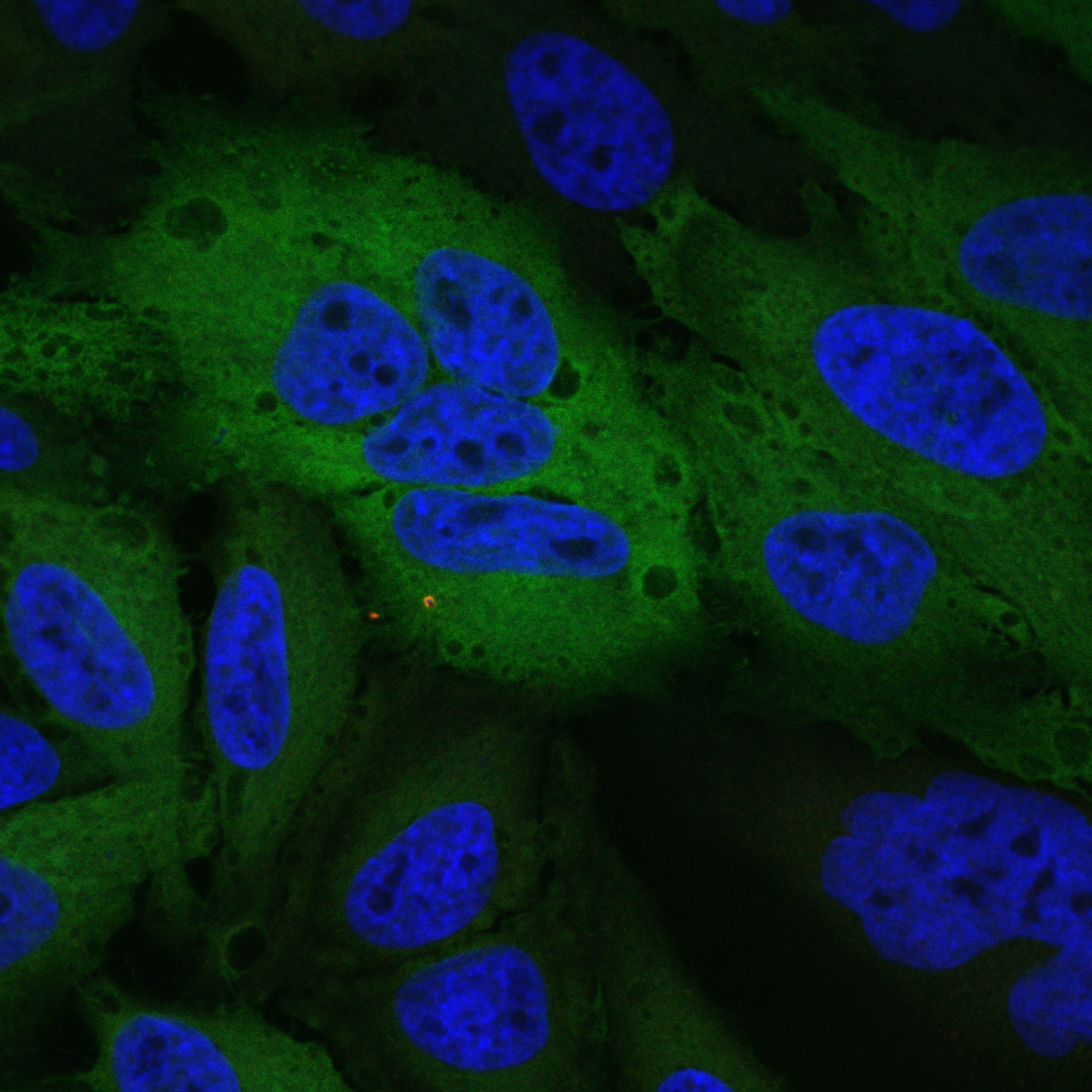

Supplement: Supplementary file 6 — Source Data for Figure 1 [file EMBJ-42-e113012-s005.zip › Figure 1/1H/Figure 1H_bottom.tif]

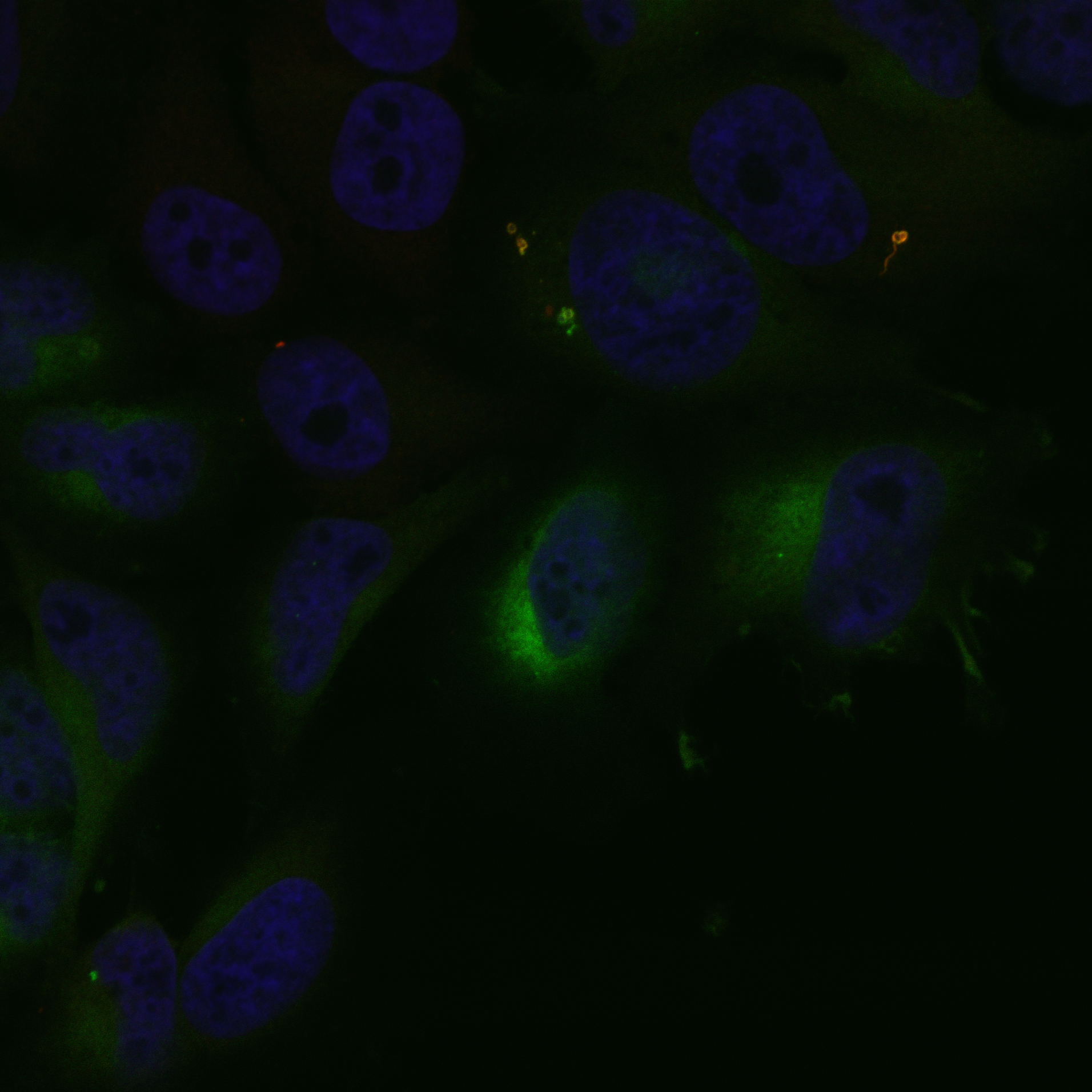

Supplement: Supplementary file 6 — Source Data for Figure 1 [file EMBJ-42-e113012-s005.zip › Figure 1/1H/Figure 1H_top.tif]

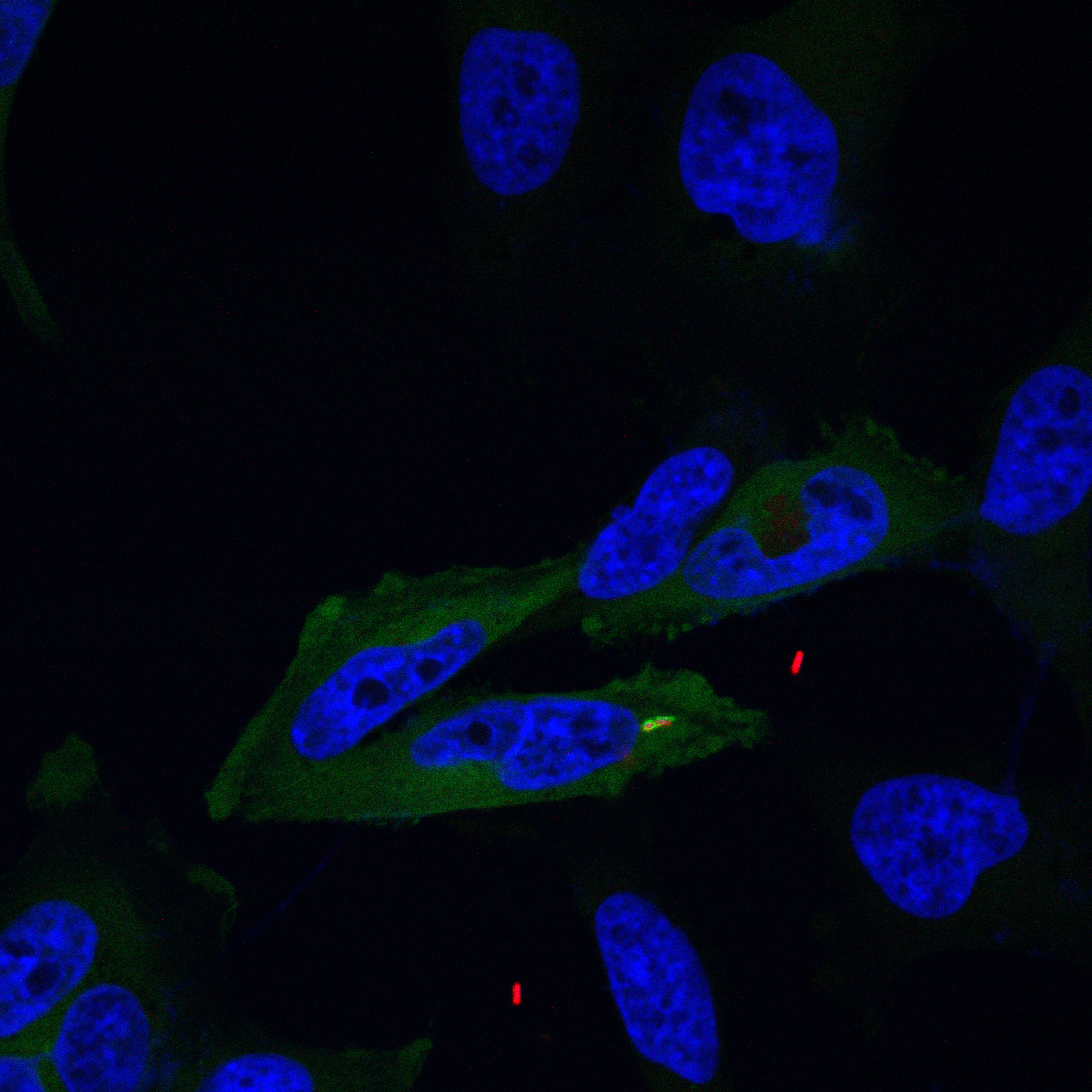

Supplement: Supplementary file 6 — Source Data for Figure 1 [file EMBJ-42-e113012-s005.zip › Figure 1/1D/Figure 1D_middle.tif]

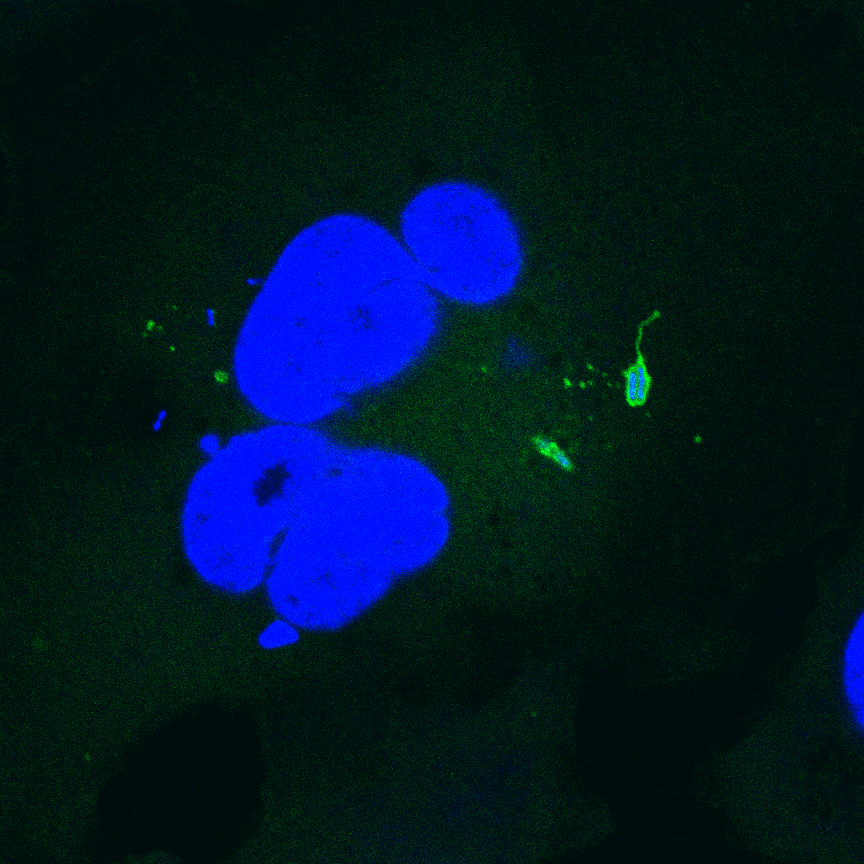

Supplement: Supplementary file 6 — Source Data for Figure 1 [file EMBJ-42-e113012-s005.zip › Figure 1/1D/Figure 1D_bottom.tif]

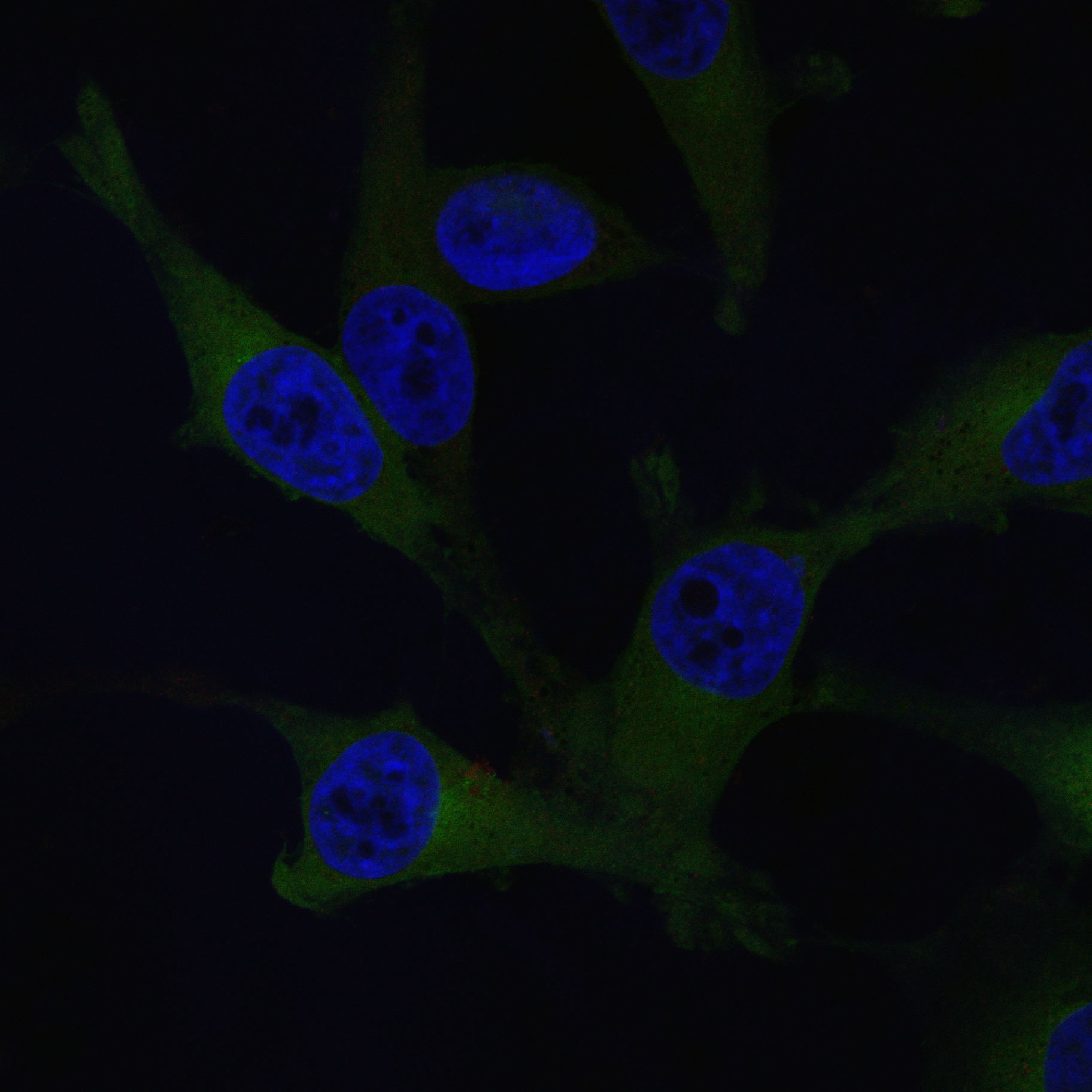

Supplement: Supplementary file 6 — Source Data for Figure 1 [file EMBJ-42-e113012-s005.zip › Figure 1/1C/Figure 1C_top.tif]

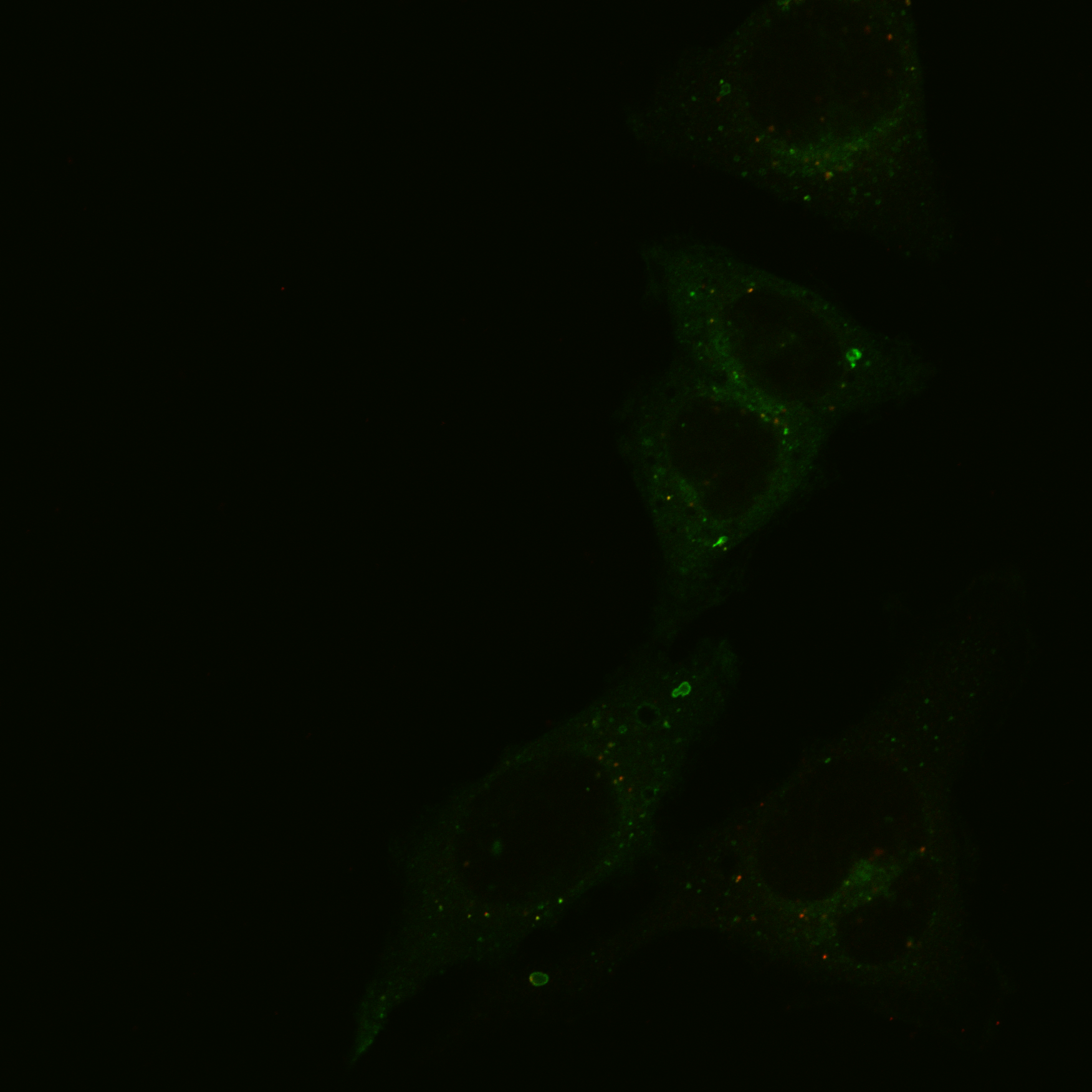

Supplement: Supplementary file 6 — Source Data for Figure 1 [file EMBJ-42-e113012-s005.zip › Figure 1/1C/Figure 1C_middle.tif]

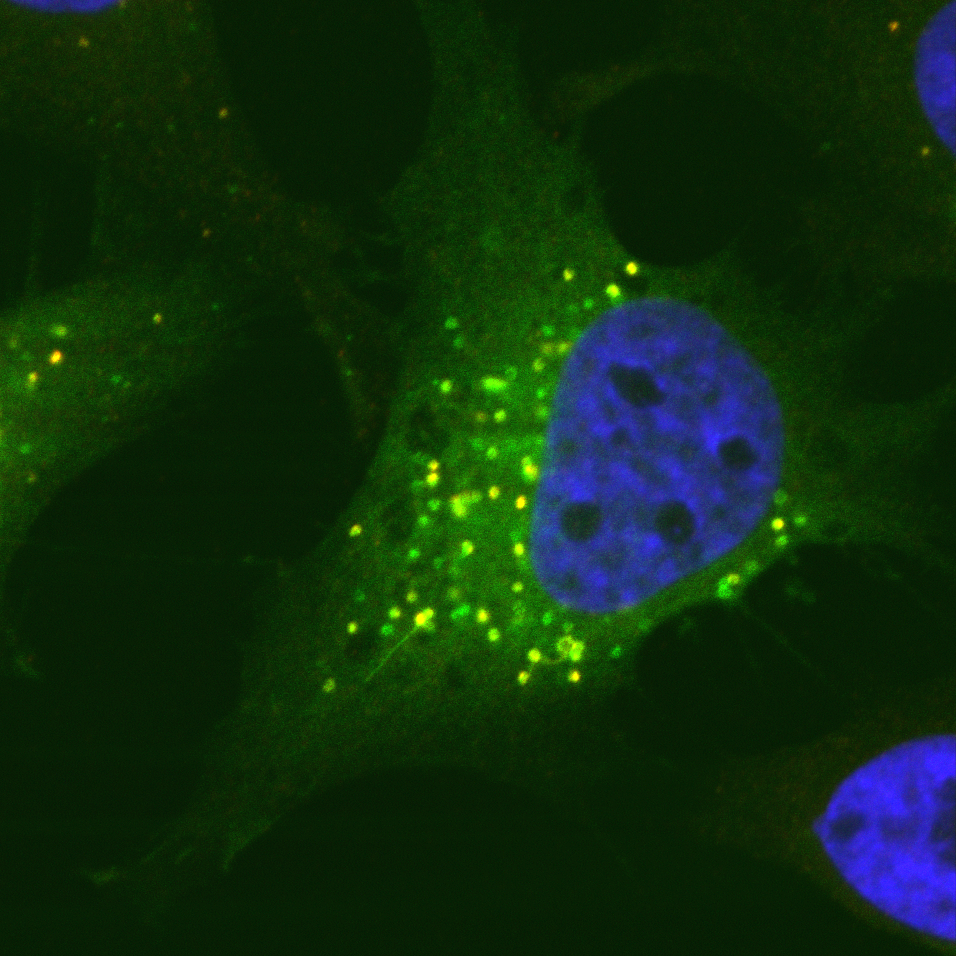

Supplement: Supplementary file 6 — Source Data for Figure 1 [file EMBJ-42-e113012-s005.zip › Figure 1/1C/Figure 1C_bottom.tif]

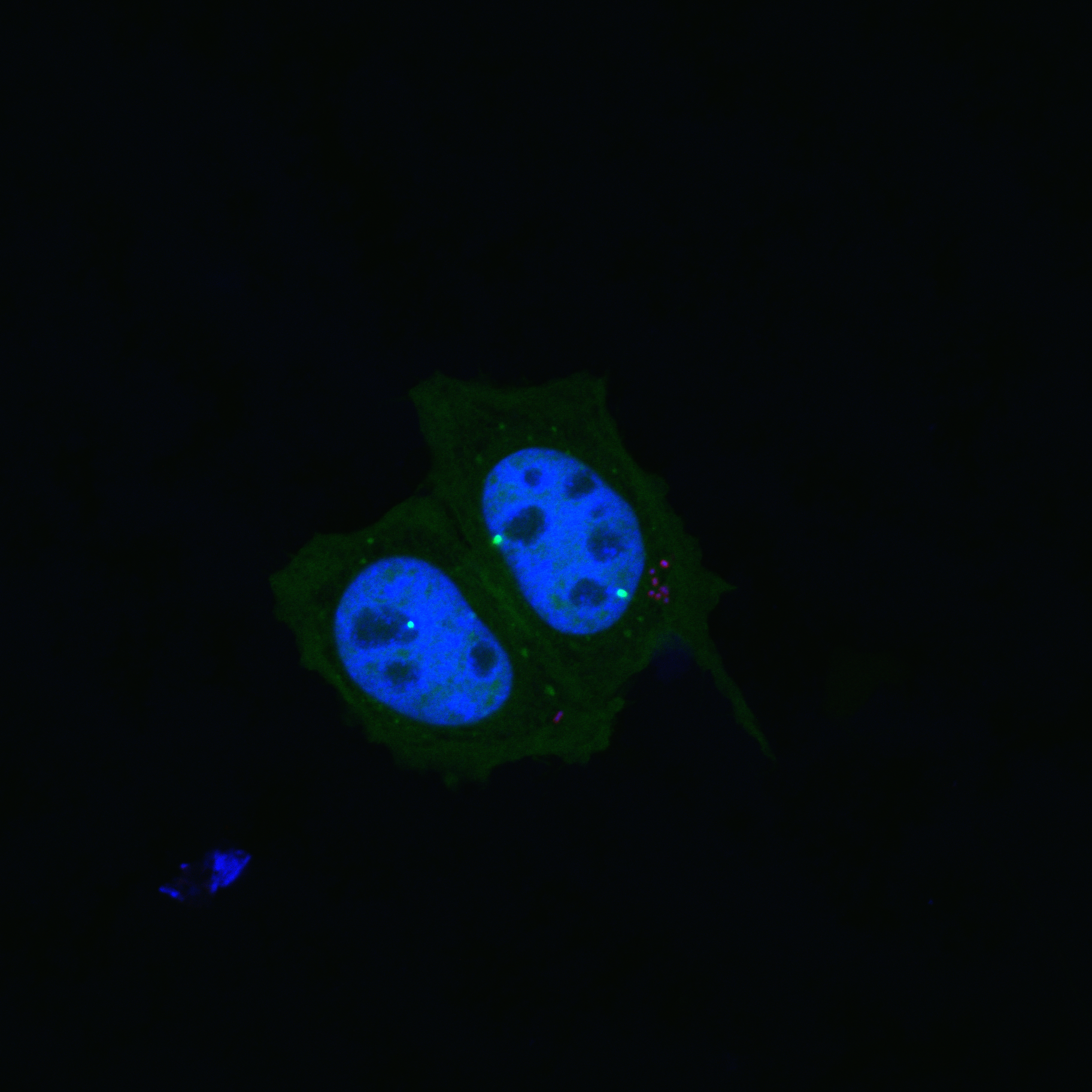

Supplement: Supplementary file 7 — Source Data for Figure 2 [file EMBJ-42-e113012-s003.zip › Figure 2/2G/Figure 2G_panel5.tif]

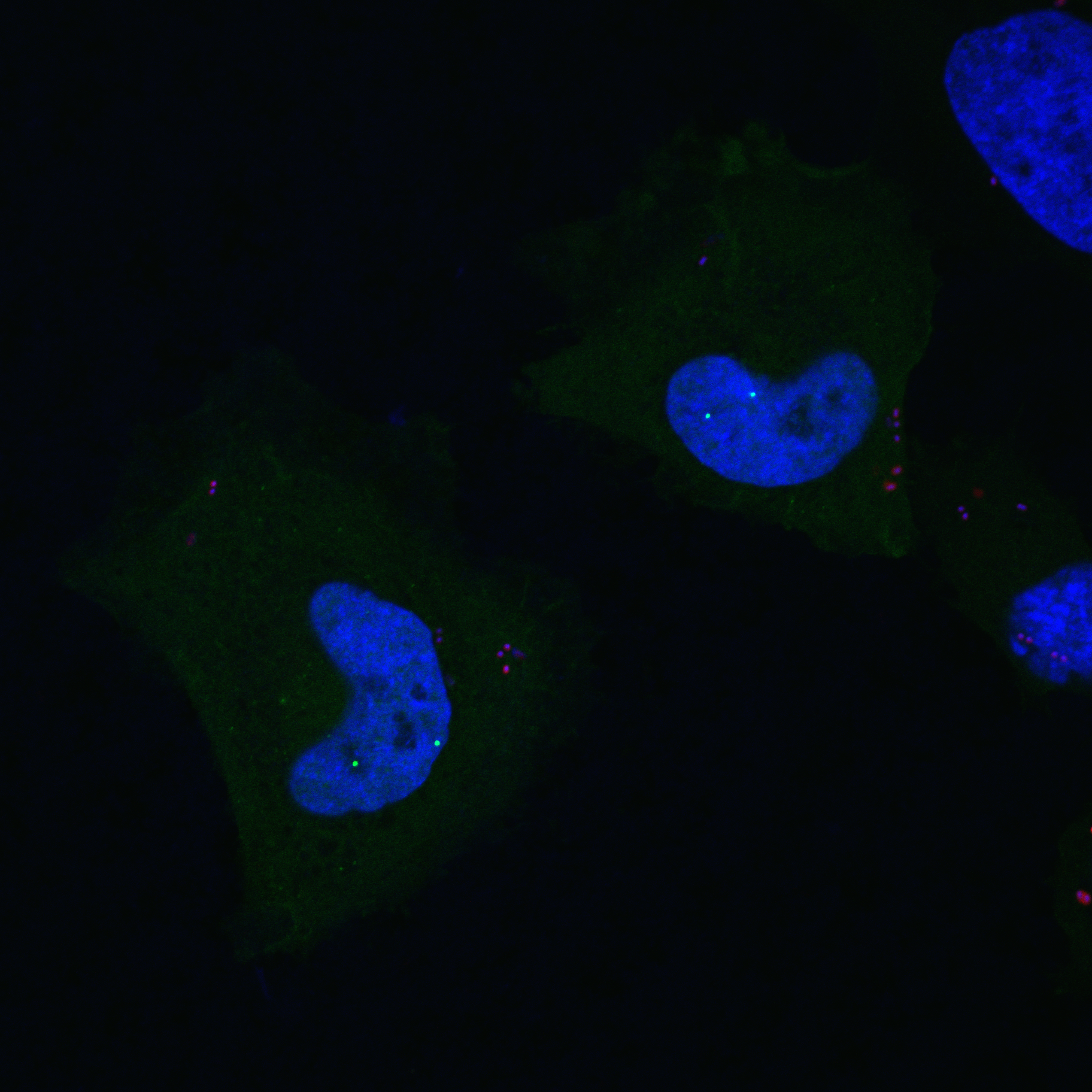

Supplement: Supplementary file 7 — Source Data for Figure 2 [file EMBJ-42-e113012-s003.zip › Figure 2/2G/Figure 2G_panel4.tif]

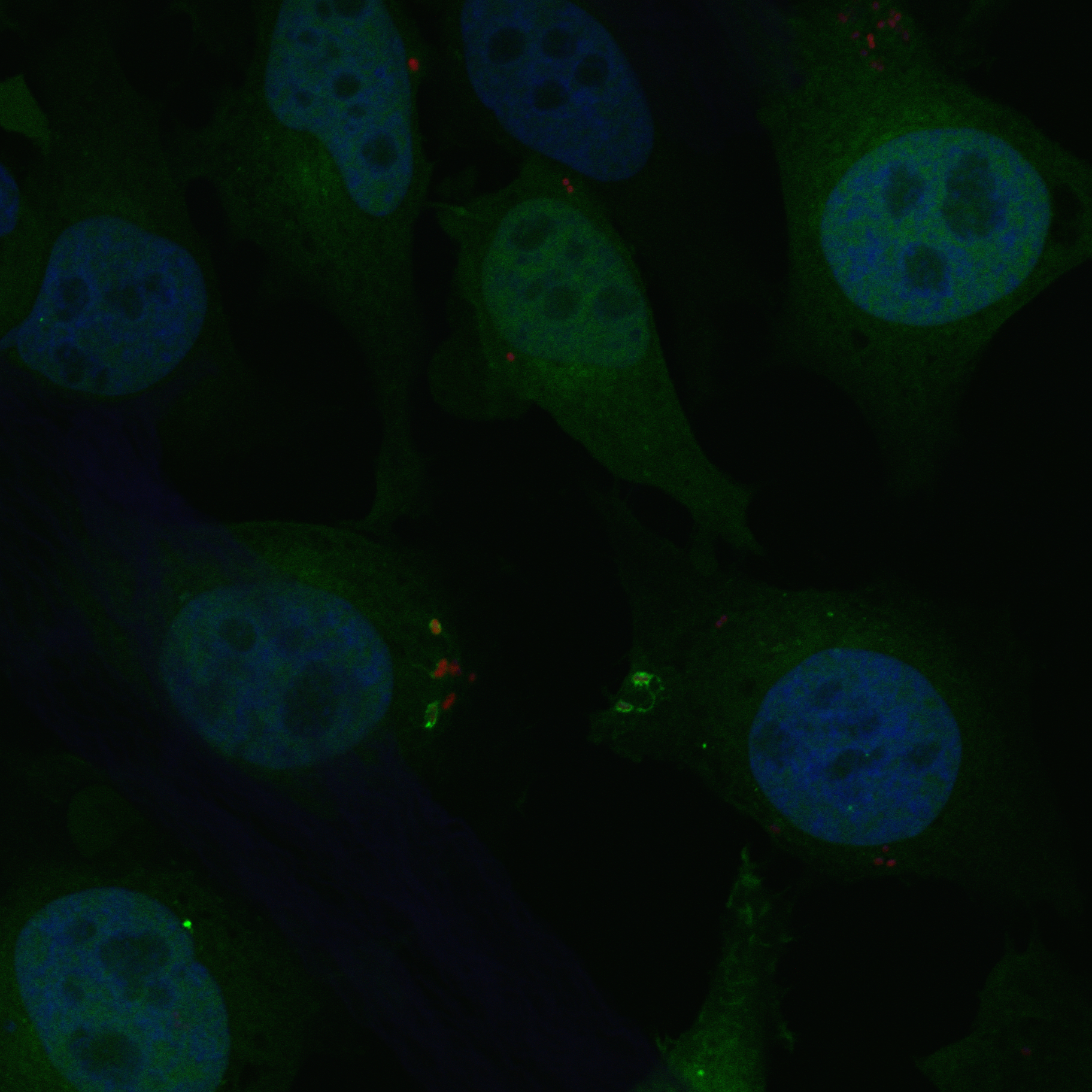

Supplement: Supplementary file 7 — Source Data for Figure 2 [file EMBJ-42-e113012-s003.zip › Figure 2/2G/Figure 2G_panel1.tif]

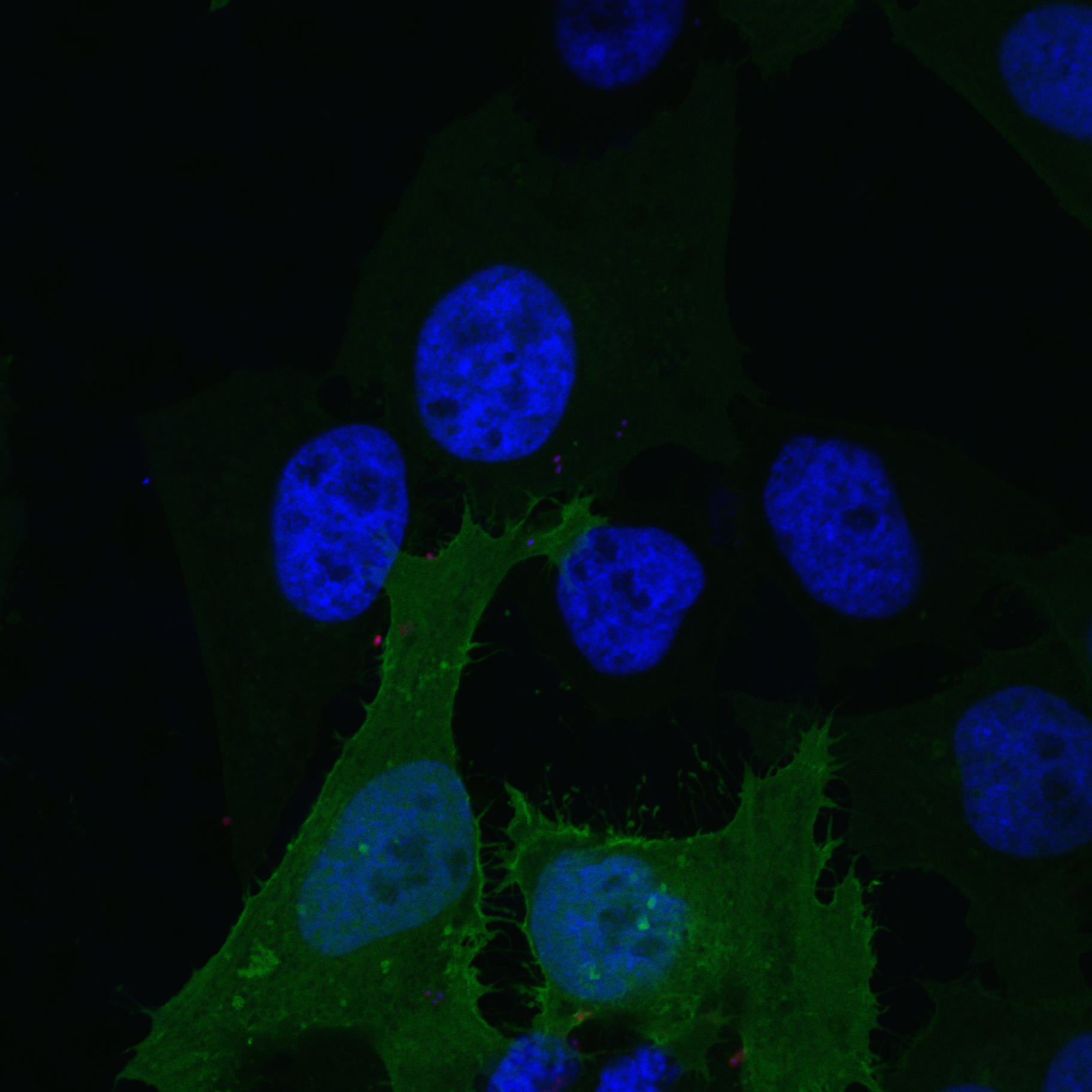

Supplement: Supplementary file 7 — Source Data for Figure 2 [file EMBJ-42-e113012-s003.zip › Figure 2/2G/Figure 2G_panel3.tif]

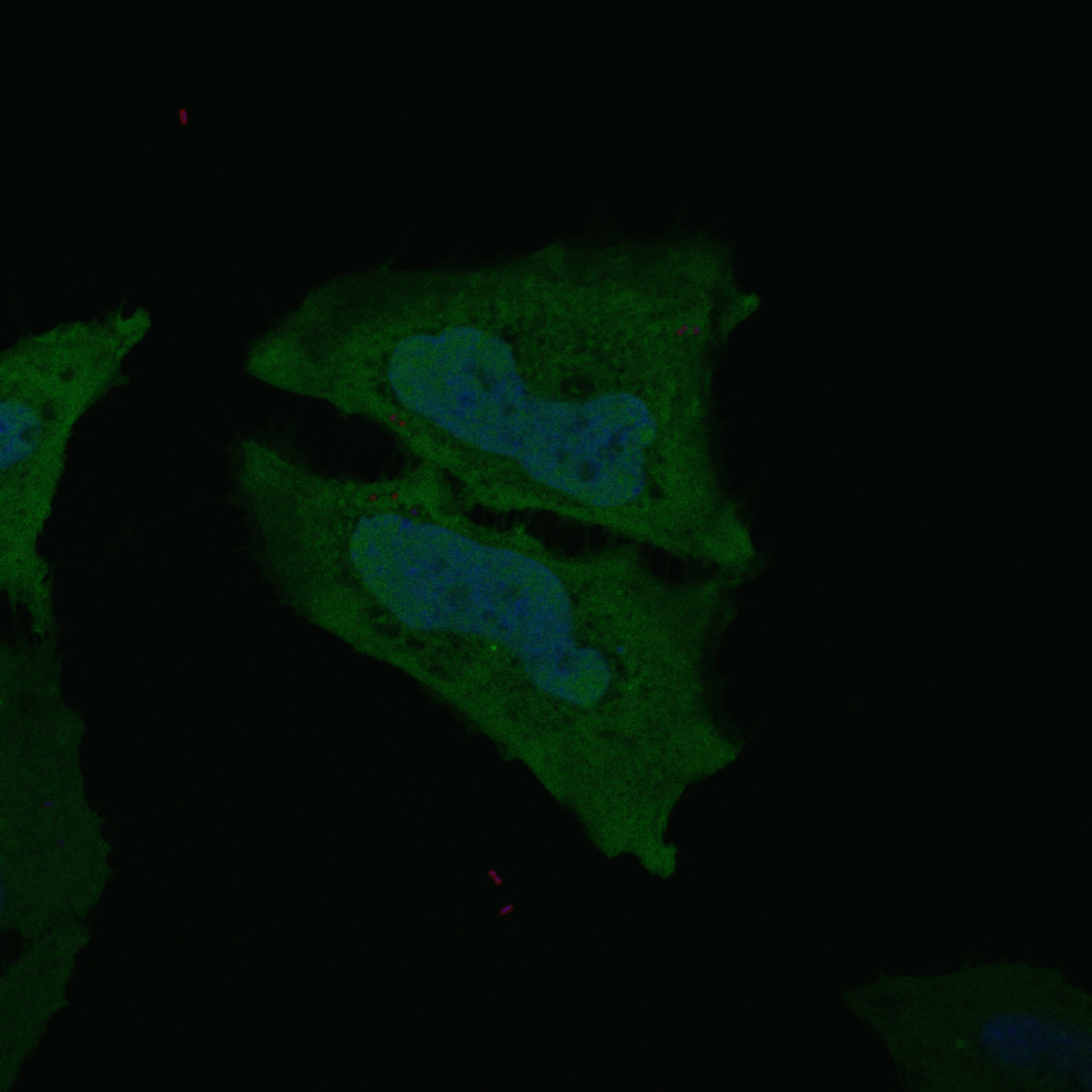

Supplement: Supplementary file 7 — Source Data for Figure 2 [file EMBJ-42-e113012-s003.zip › Figure 2/2G/Figure 2G_panel2.tif]

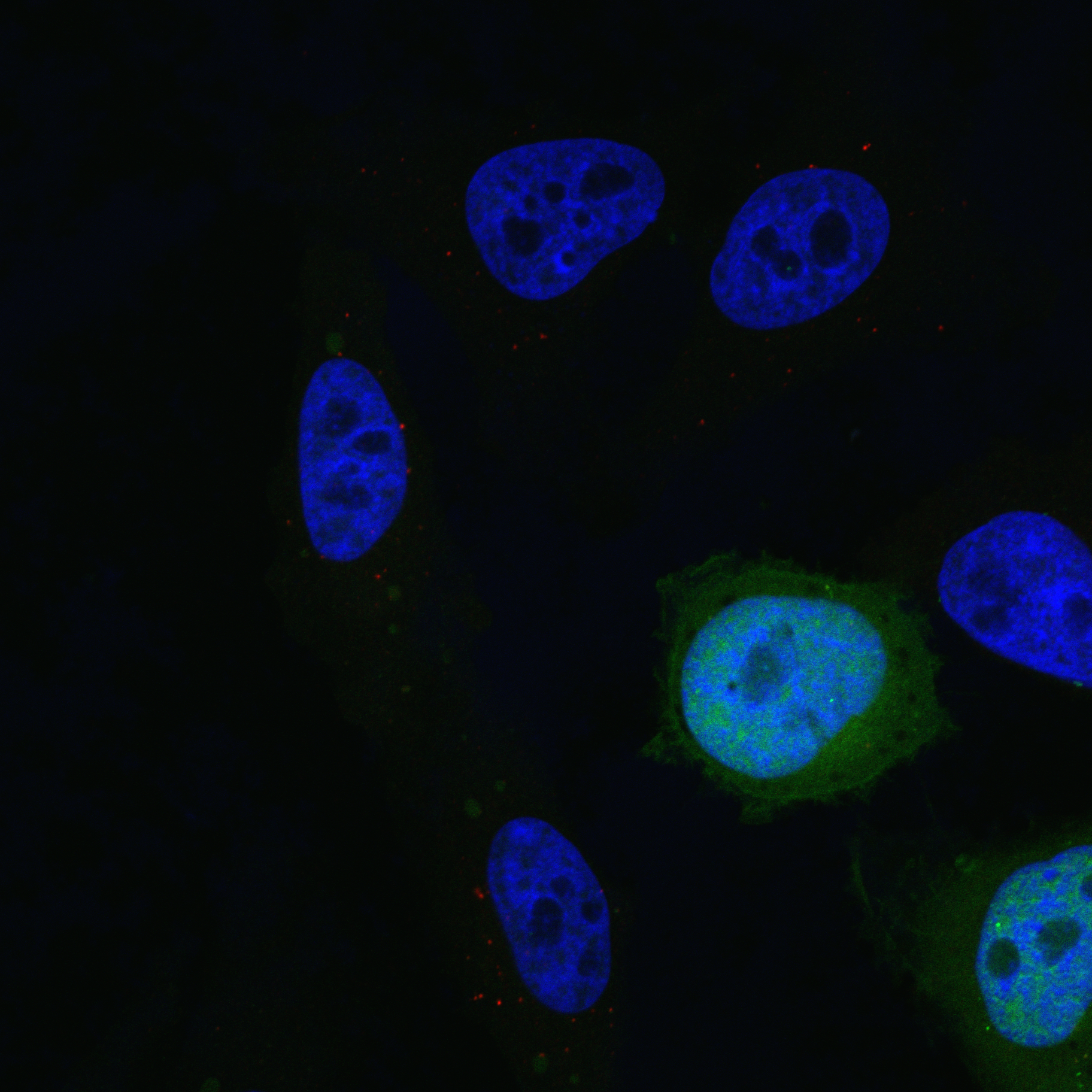

Supplement: Supplementary file 7 — Source Data for Figure 2 [file EMBJ-42-e113012-s003.zip › Figure 2/2H/Figure 2H_panel4.tif]

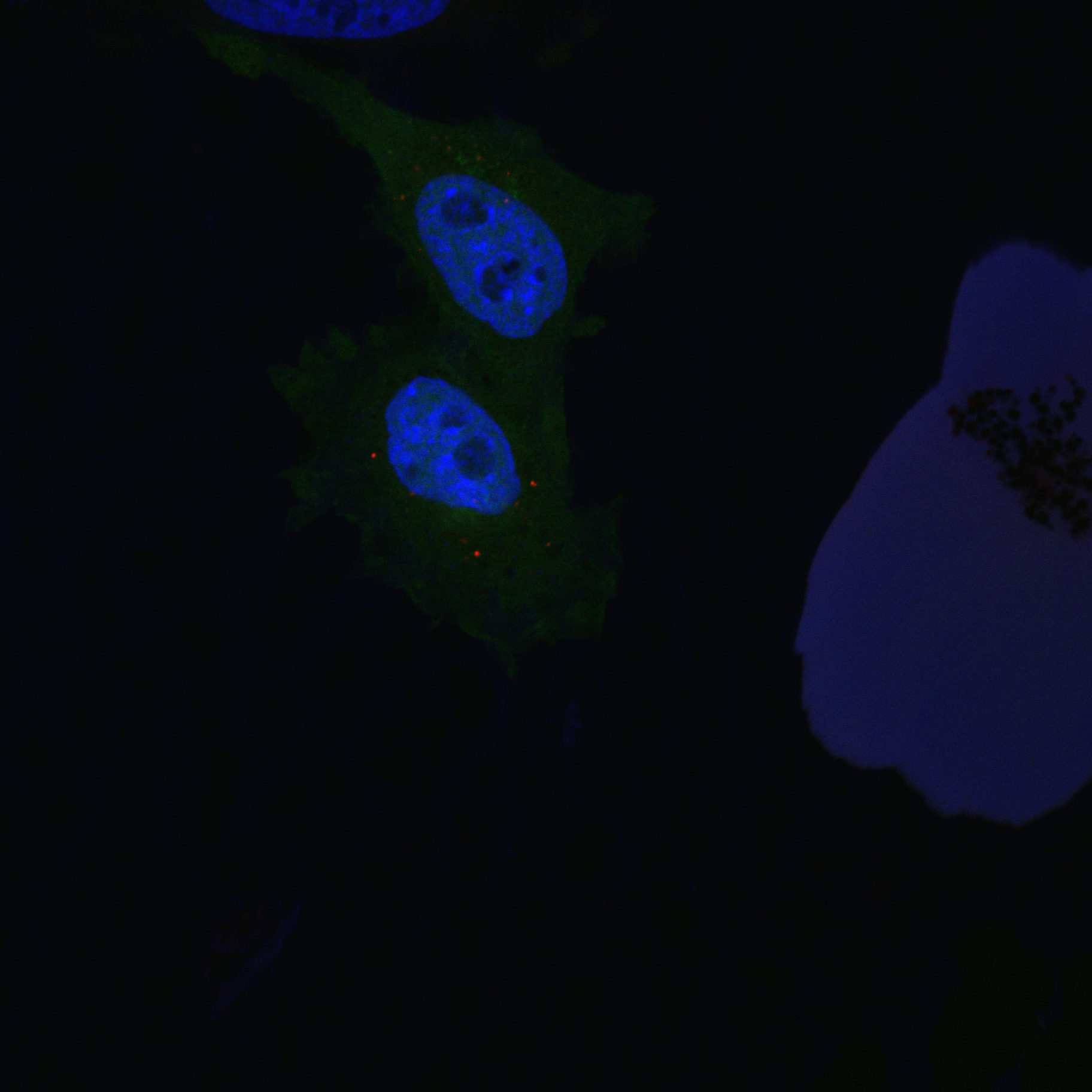

Supplement: Supplementary file 7 — Source Data for Figure 2 [file EMBJ-42-e113012-s003.zip › Figure 2/2H/Figure 2H_panel5.tif]

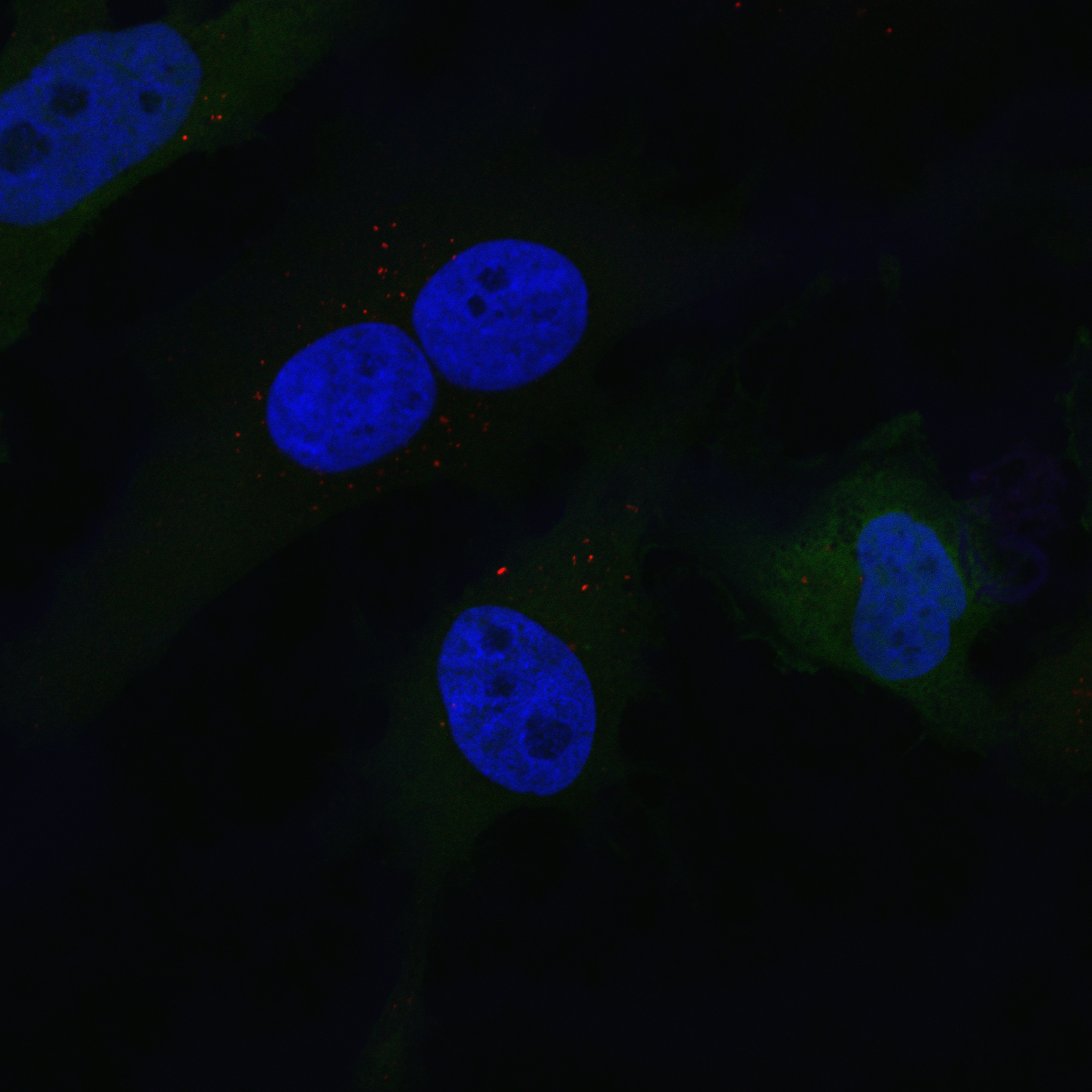

Supplement: Supplementary file 7 — Source Data for Figure 2 [file EMBJ-42-e113012-s003.zip › Figure 2/2H/Figure 2H_panel2.tif]

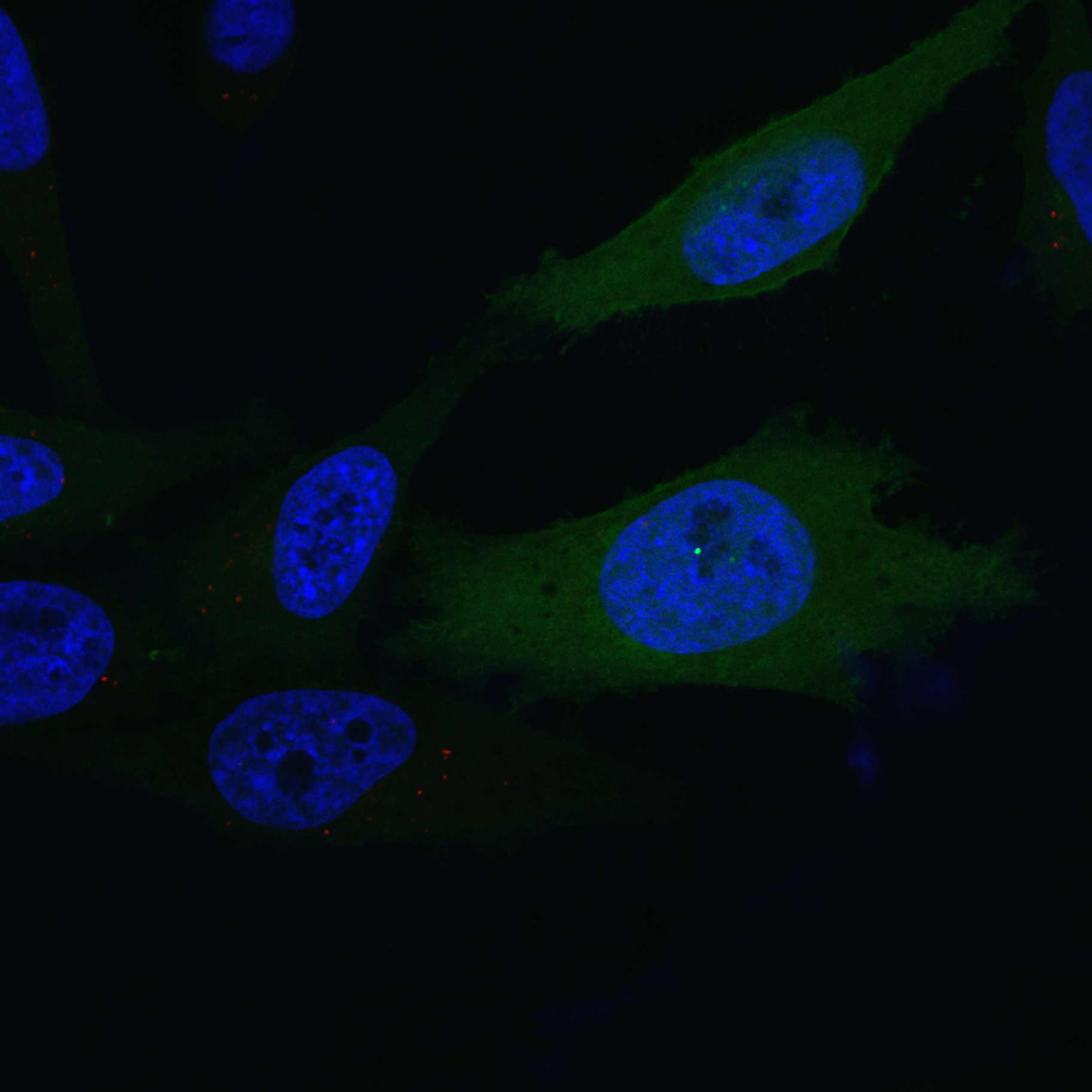

Supplement: Supplementary file 7 — Source Data for Figure 2 [file EMBJ-42-e113012-s003.zip › Figure 2/2H/Figure 2H_panel3.tif]

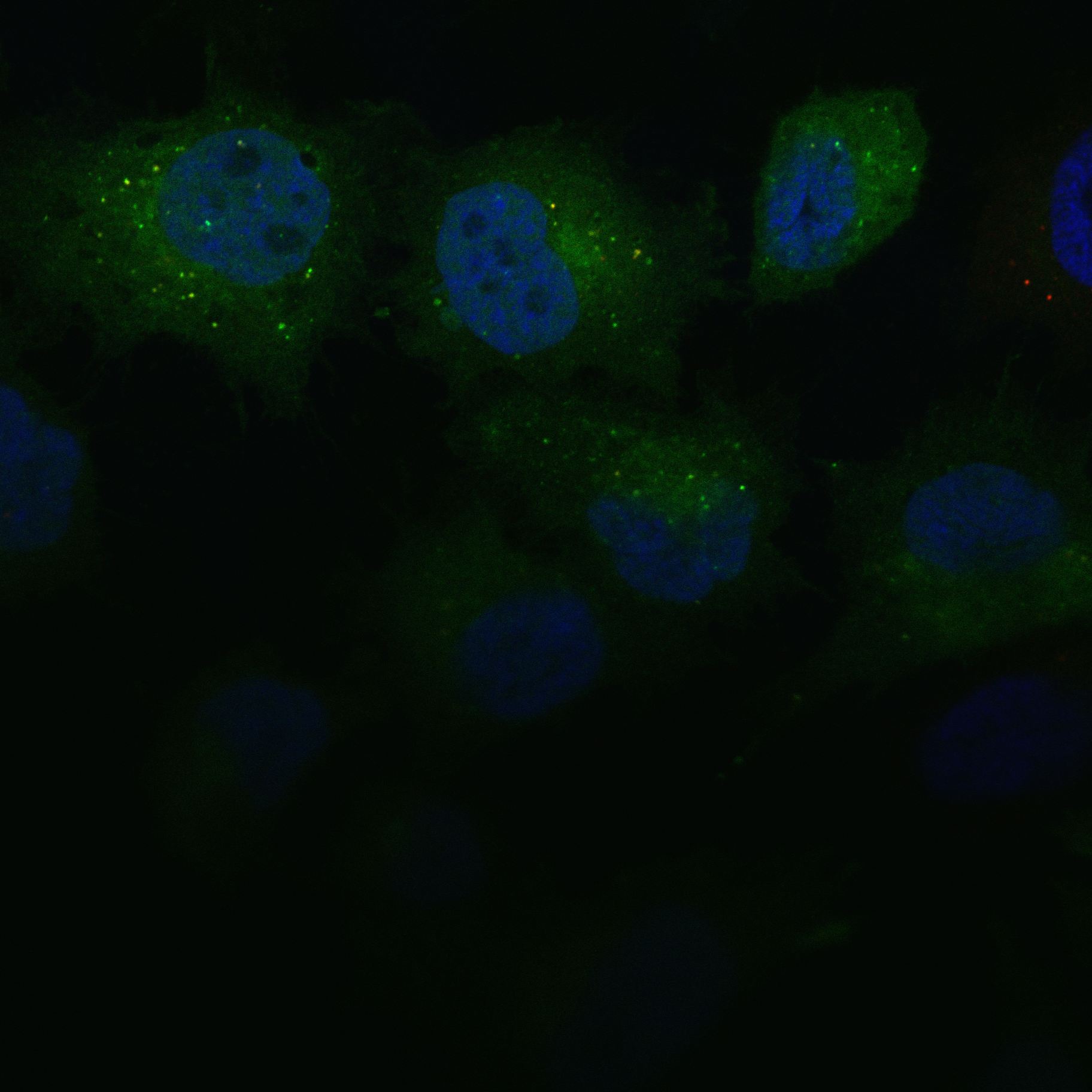

Supplement: Supplementary file 7 — Source Data for Figure 2 [file EMBJ-42-e113012-s003.zip › Figure 2/2H/Figure 2H_panel1.tif]

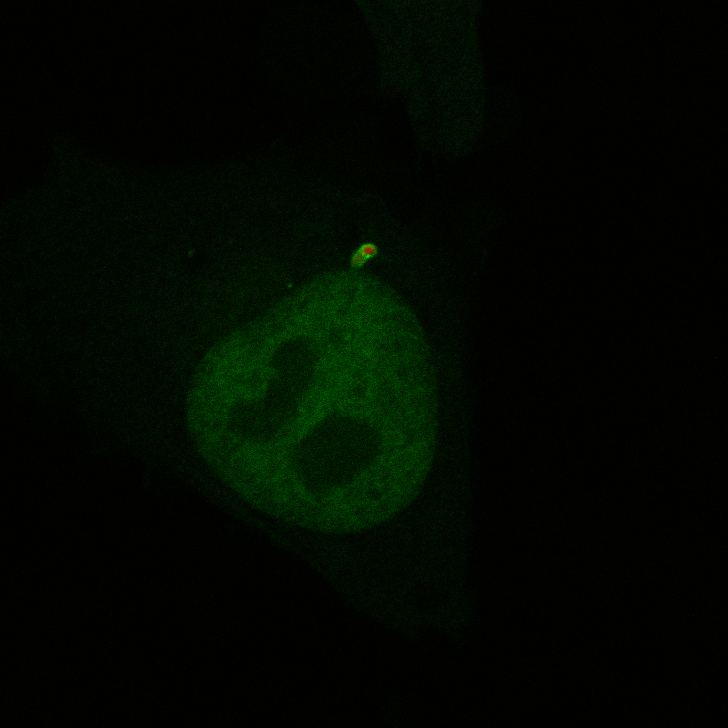

Supplement: Supplementary file 7 — Source Data for Figure 2 [file EMBJ-42-e113012-s003.zip › Figure 2/2D/Figure 2D.tif]

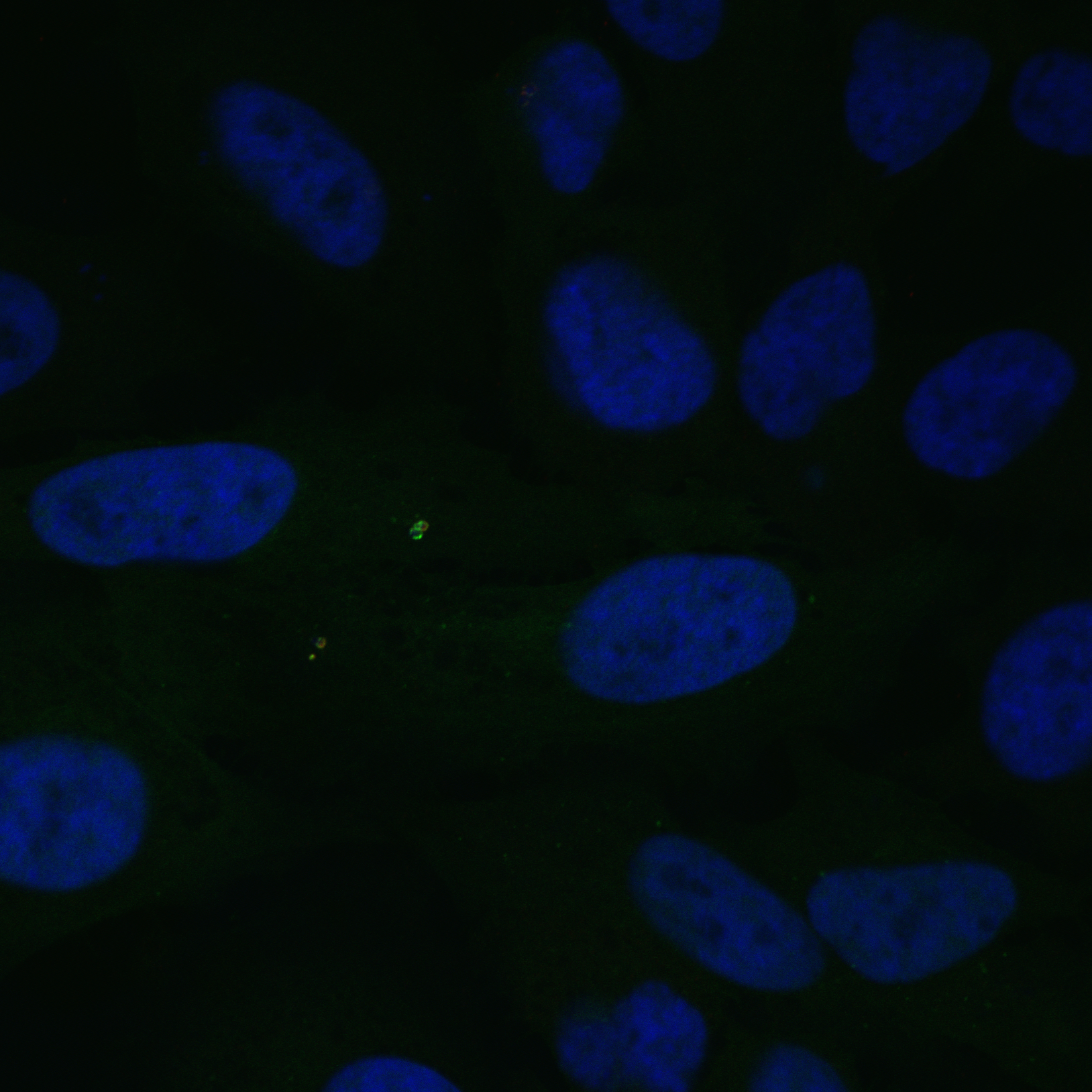

Supplement: Supplementary file 7 — Source Data for Figure 2 [file EMBJ-42-e113012-s003.zip › Figure 2/2E/Figure 2E_top.tif]

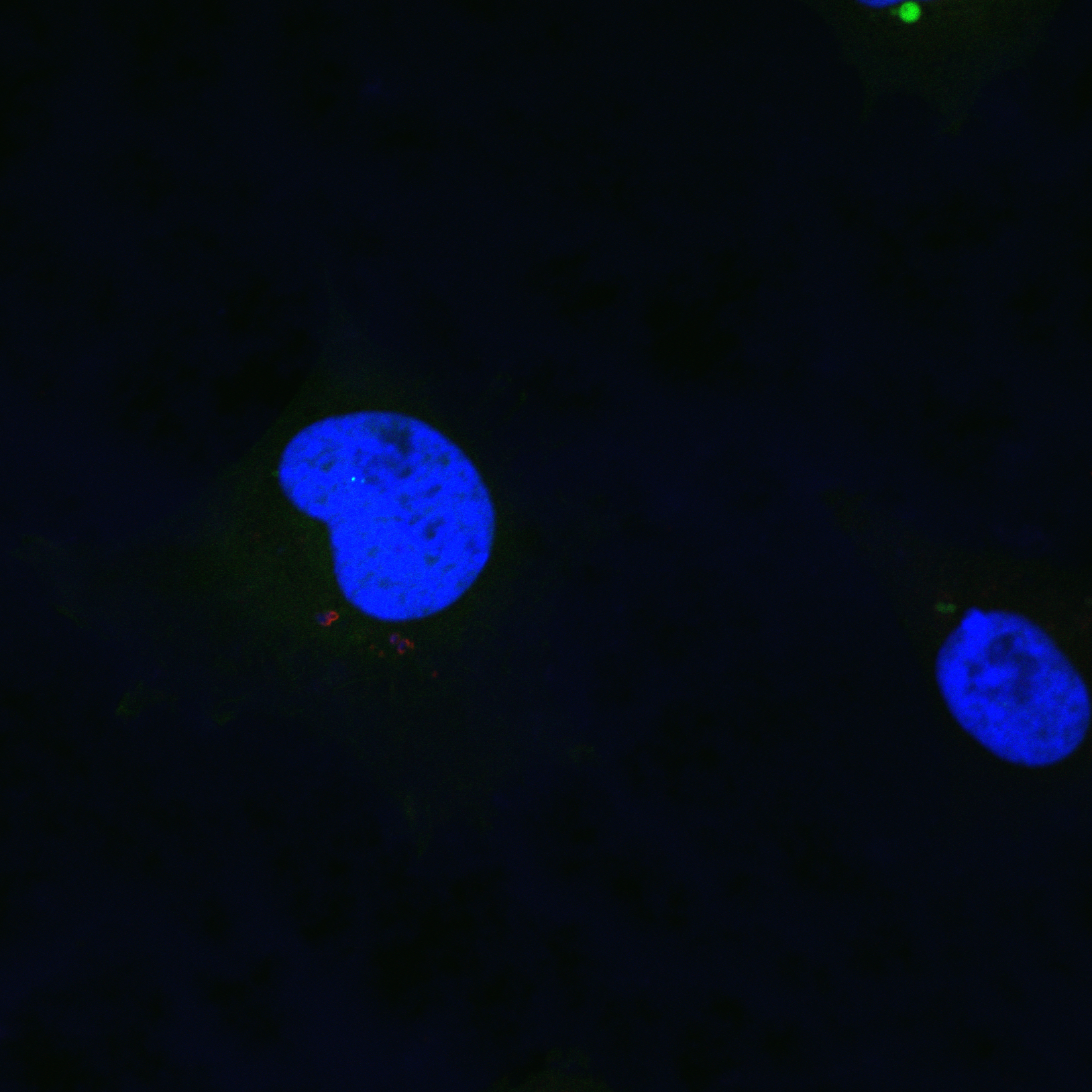

Supplement: Supplementary file 7 — Source Data for Figure 2 [file EMBJ-42-e113012-s003.zip › Figure 2/2E/Figure 2E_bottom.tif]

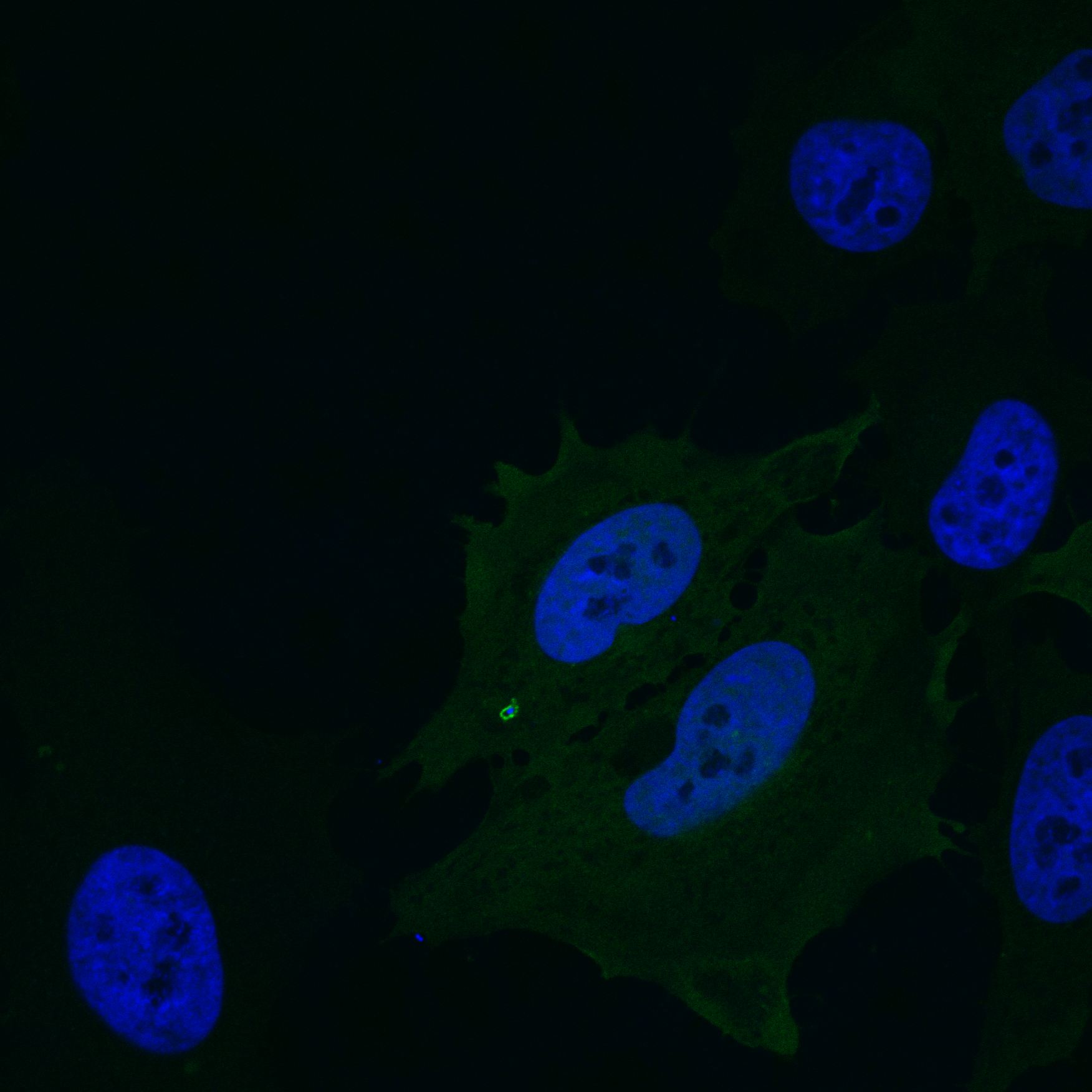

Supplement: Supplementary file 7 — Source Data for Figure 2 [file EMBJ-42-e113012-s003.zip › Figure 2/2B/Figure 2B_bottom.tif]

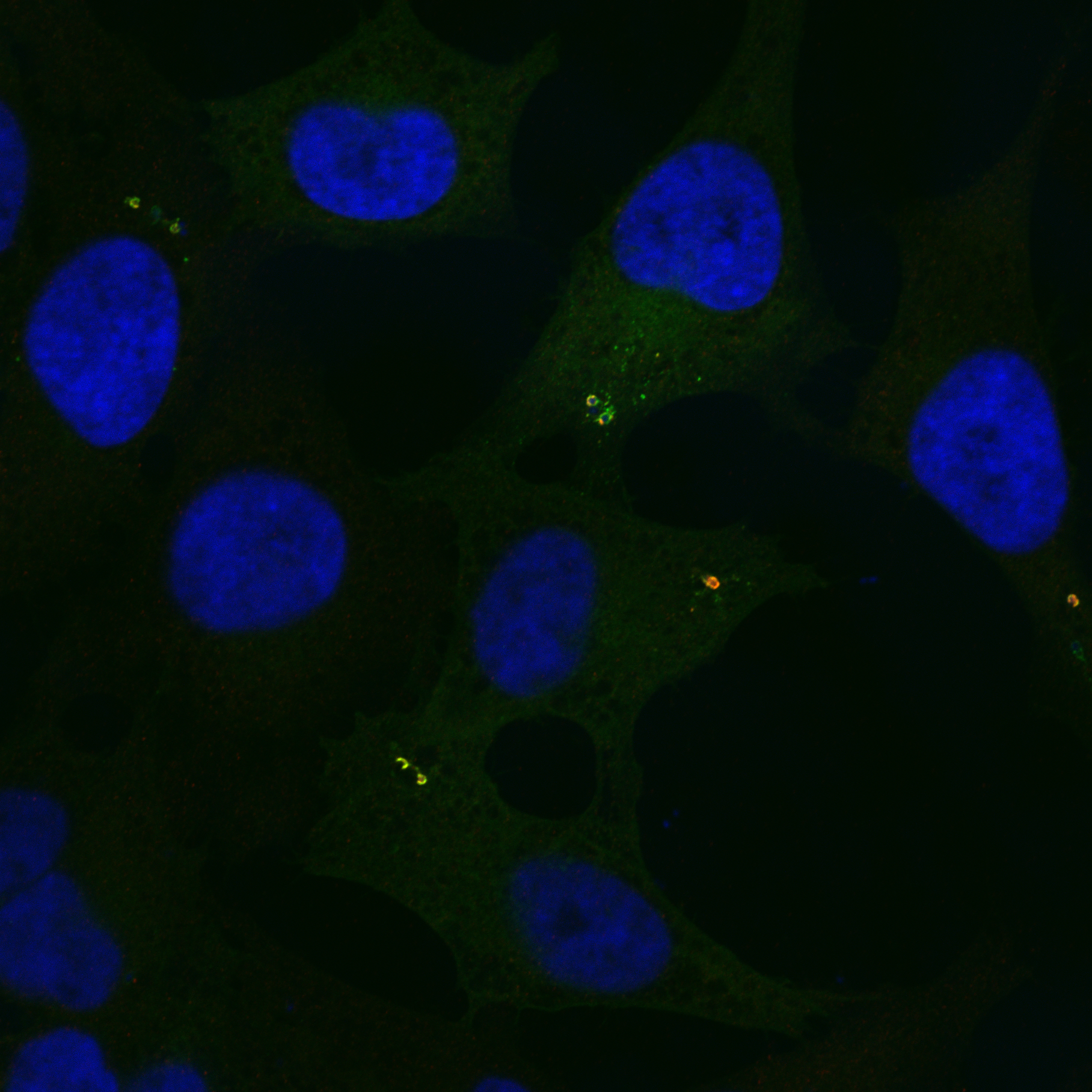

Supplement: Supplementary file 7 — Source Data for Figure 2 [file EMBJ-42-e113012-s003.zip › Figure 2/2B/Figure 2B_top.tif]

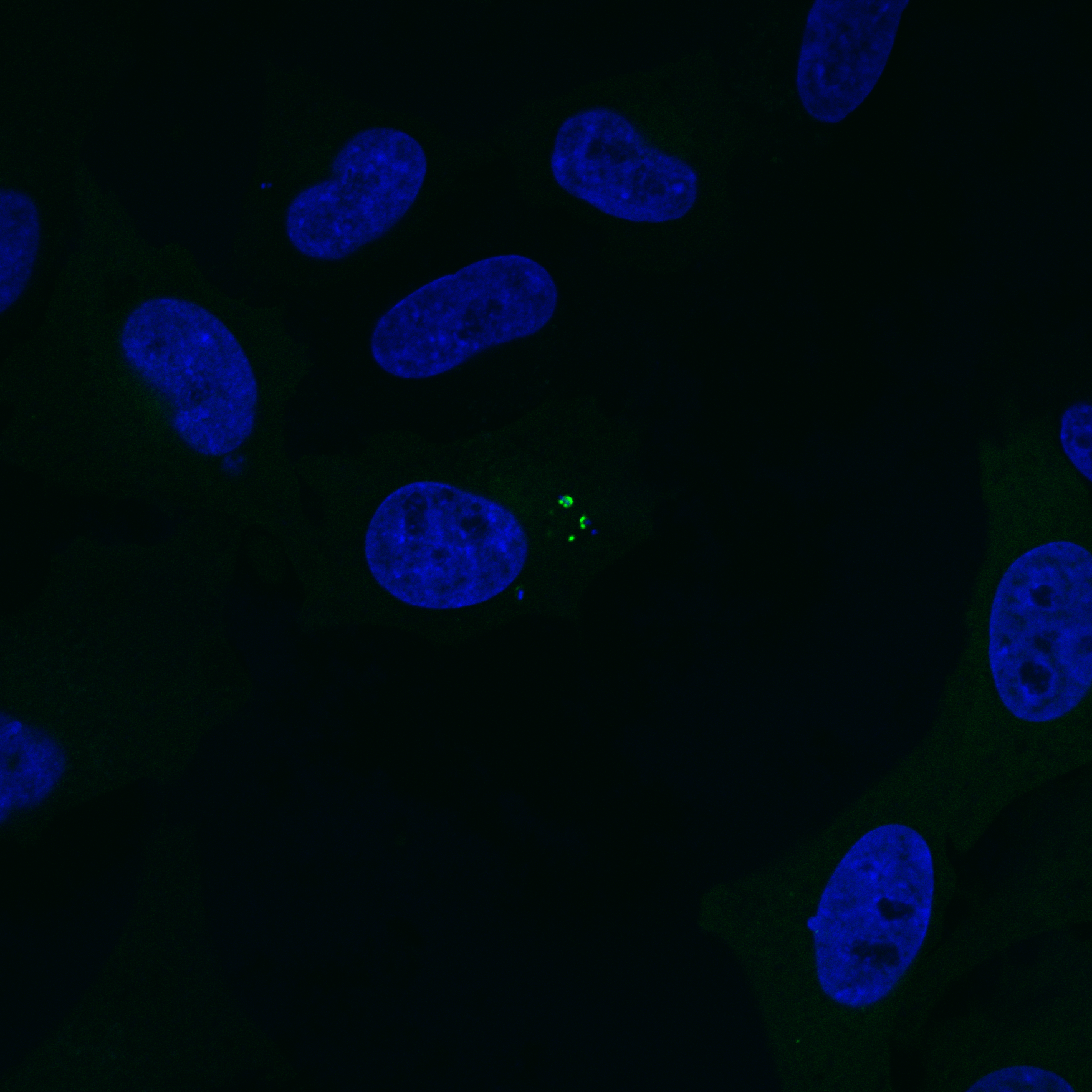

Supplement: Supplementary file 7 — Source Data for Figure 2 [file EMBJ-42-e113012-s003.zip › Figure 2/2B/Figure 2B_middle.tif]

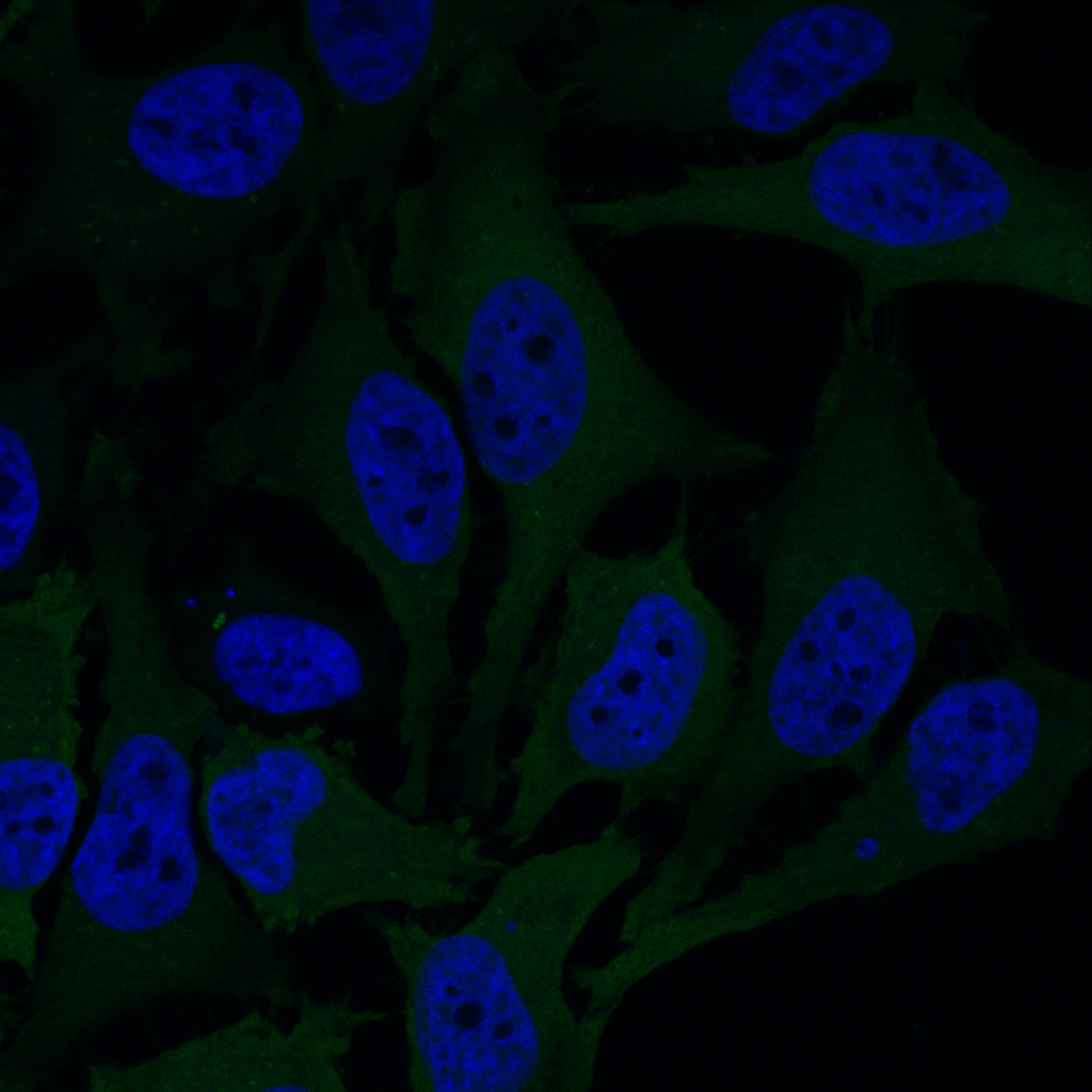

Supplement: Supplementary file 8 — Source Data for Figure 3 [file EMBJ-42-e113012-s011.zip › Figure 3/3F/Figure 3F_bottom right.tif]

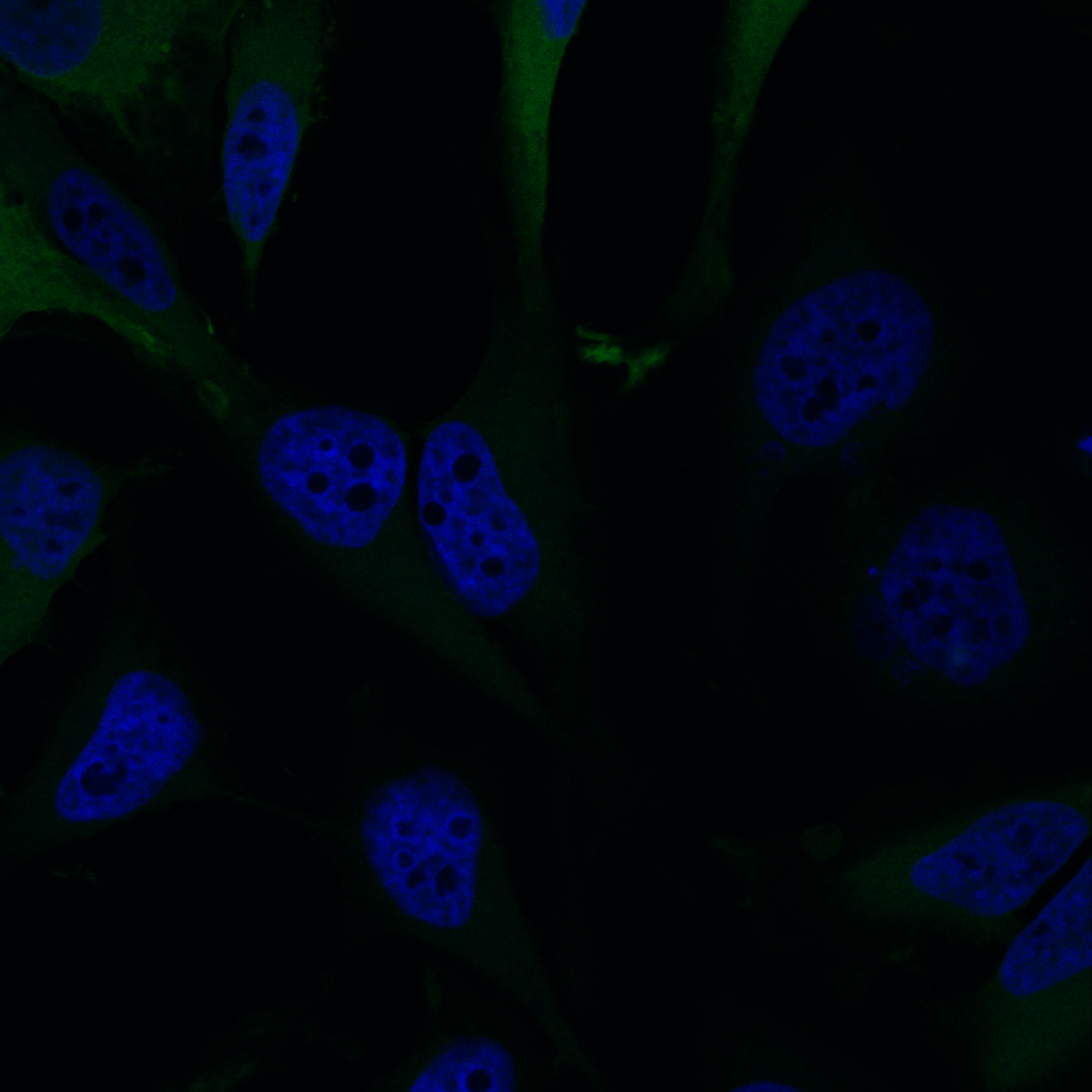

Supplement: Supplementary file 8 — Source Data for Figure 3 [file EMBJ-42-e113012-s011.zip › Figure 3/3F/Figure 3F_bottom left.tif]

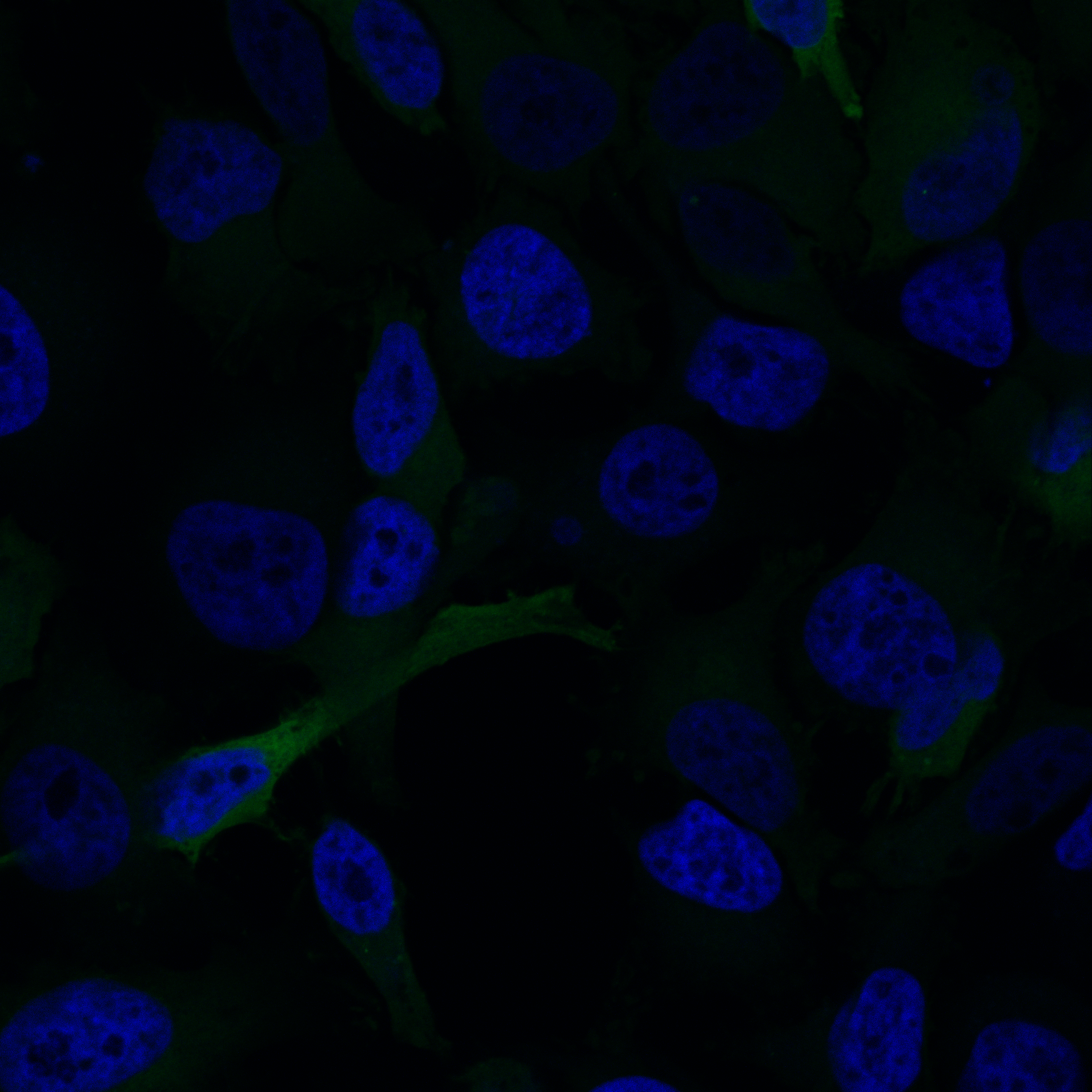

Supplement: Supplementary file 8 — Source Data for Figure 3 [file EMBJ-42-e113012-s011.zip › Figure 3/3F/Figure 3F_top left.tif]

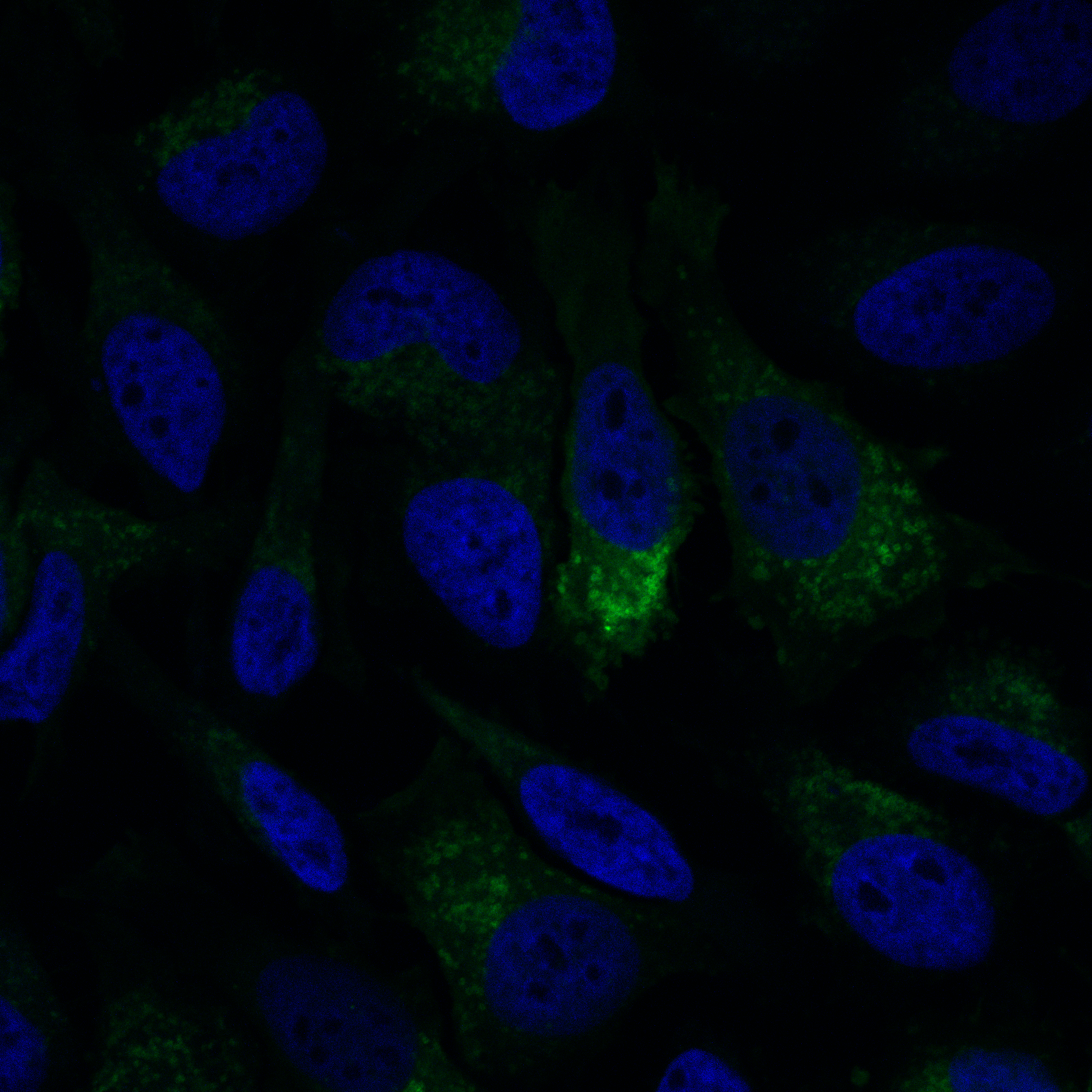

Supplement: Supplementary file 8 — Source Data for Figure 3 [file EMBJ-42-e113012-s011.zip › Figure 3/3F/Figure 3F_top right.tif]

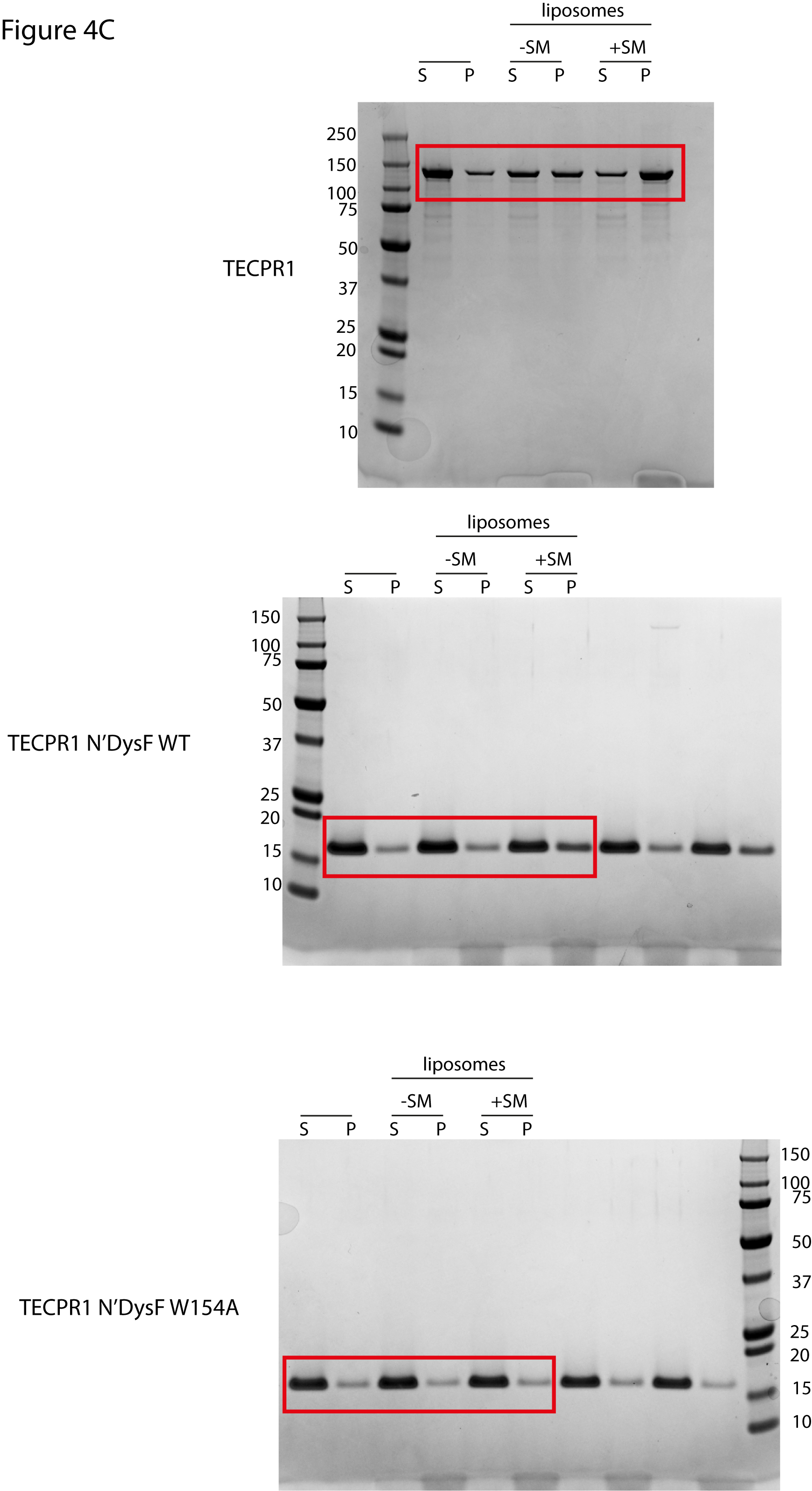

Supplement: Supplementary file 9 — Source Data for Figure 4 [file EMBJ-42-e113012-s002.zip › Figure 4/4C/Coomassie.tif]

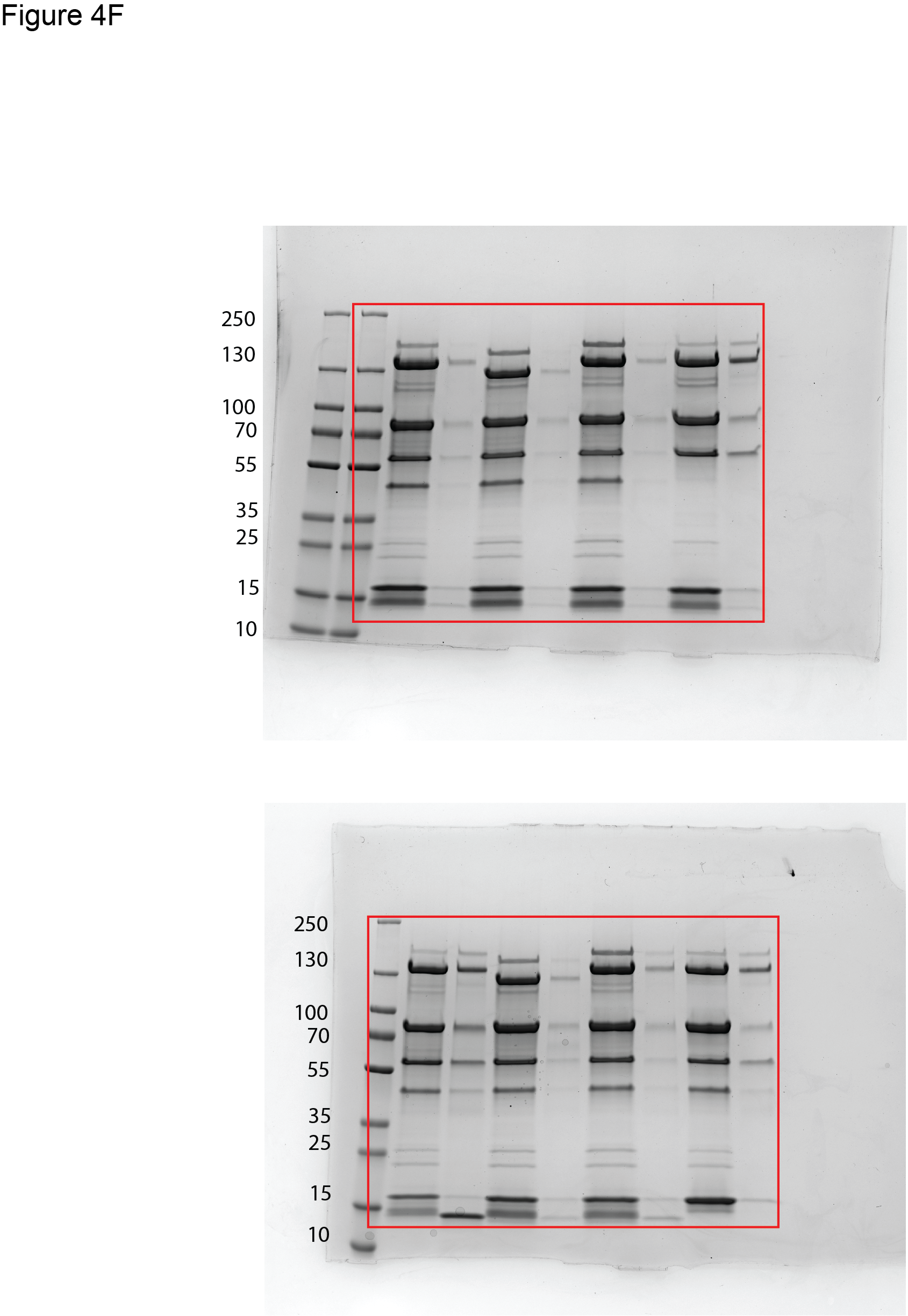

Supplement: Supplementary file 9 — Source Data for Figure 4 [file EMBJ-42-e113012-s002.zip › Figure 4/4F/Coomassie 4F.tif]

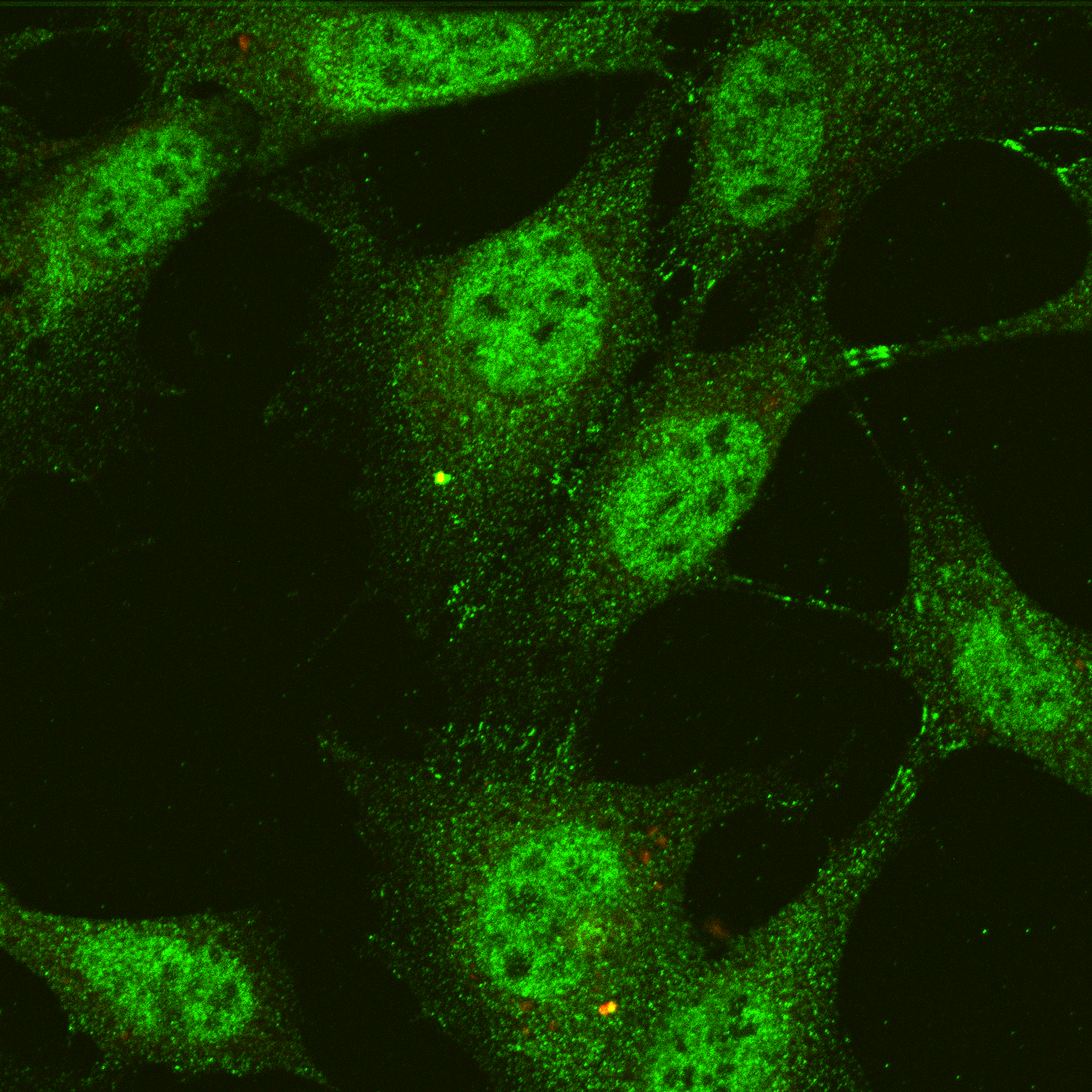

Supplement: Supplementary file 10 — Source Data for Figure 5 [file EMBJ-42-e113012-s010.zip › Figure 5/5A/Figure 5A_panel1.tif]

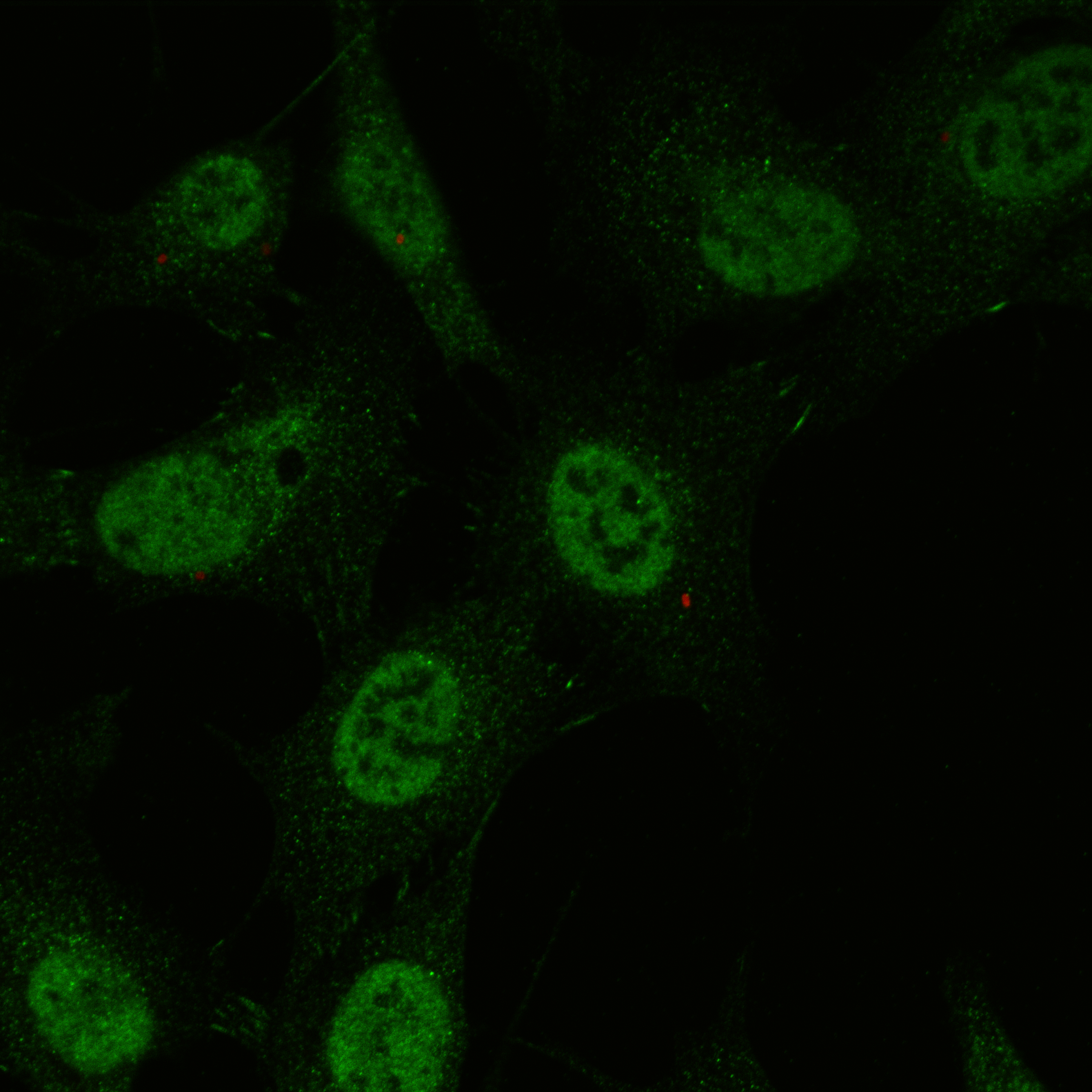

Supplement: Supplementary file 10 — Source Data for Figure 5 [file EMBJ-42-e113012-s010.zip › Figure 5/5A/Figure 5A_panel2.tif]

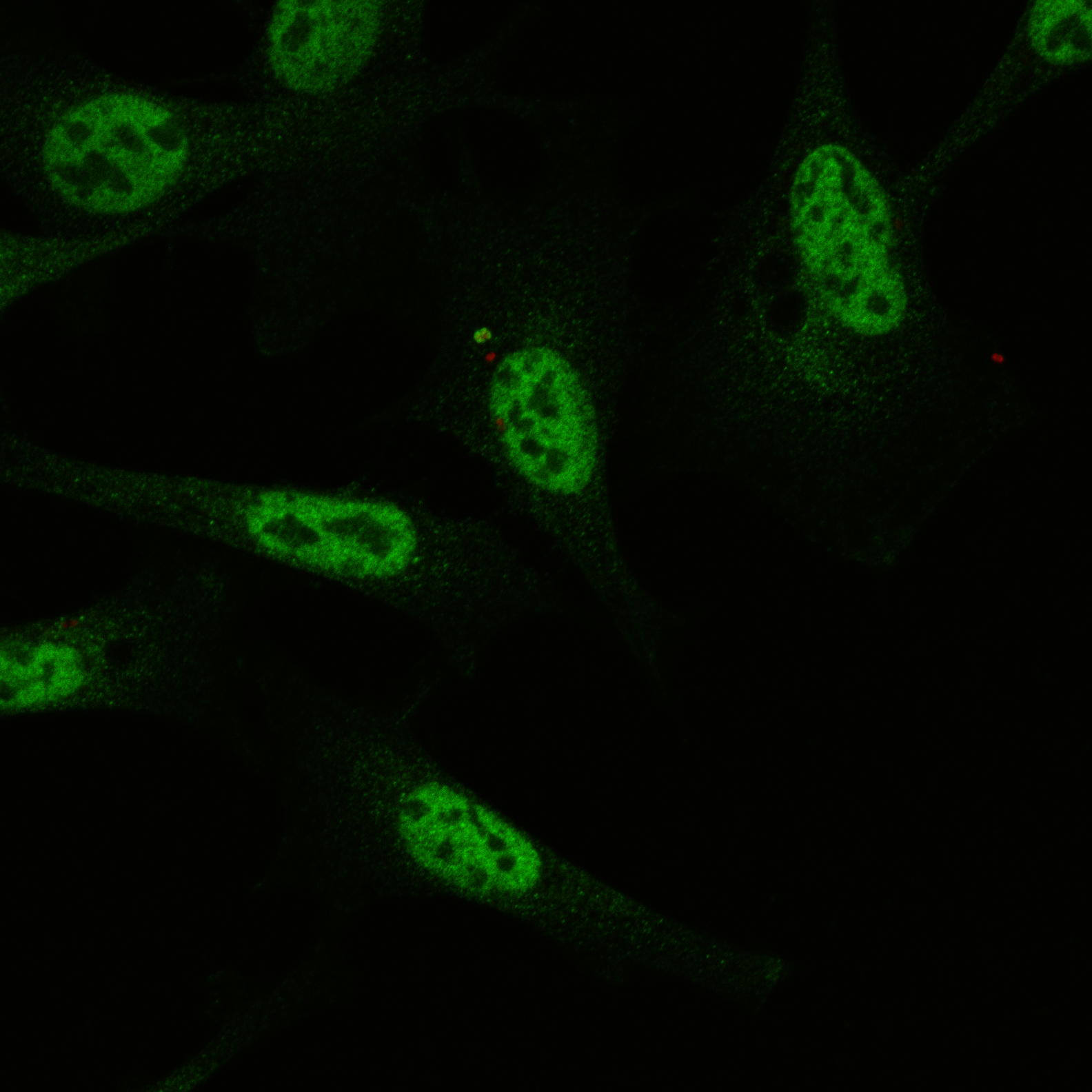

Supplement: Supplementary file 10 — Source Data for Figure 5 [file EMBJ-42-e113012-s010.zip › Figure 5/5A/Figure 5A_panel3.tif]

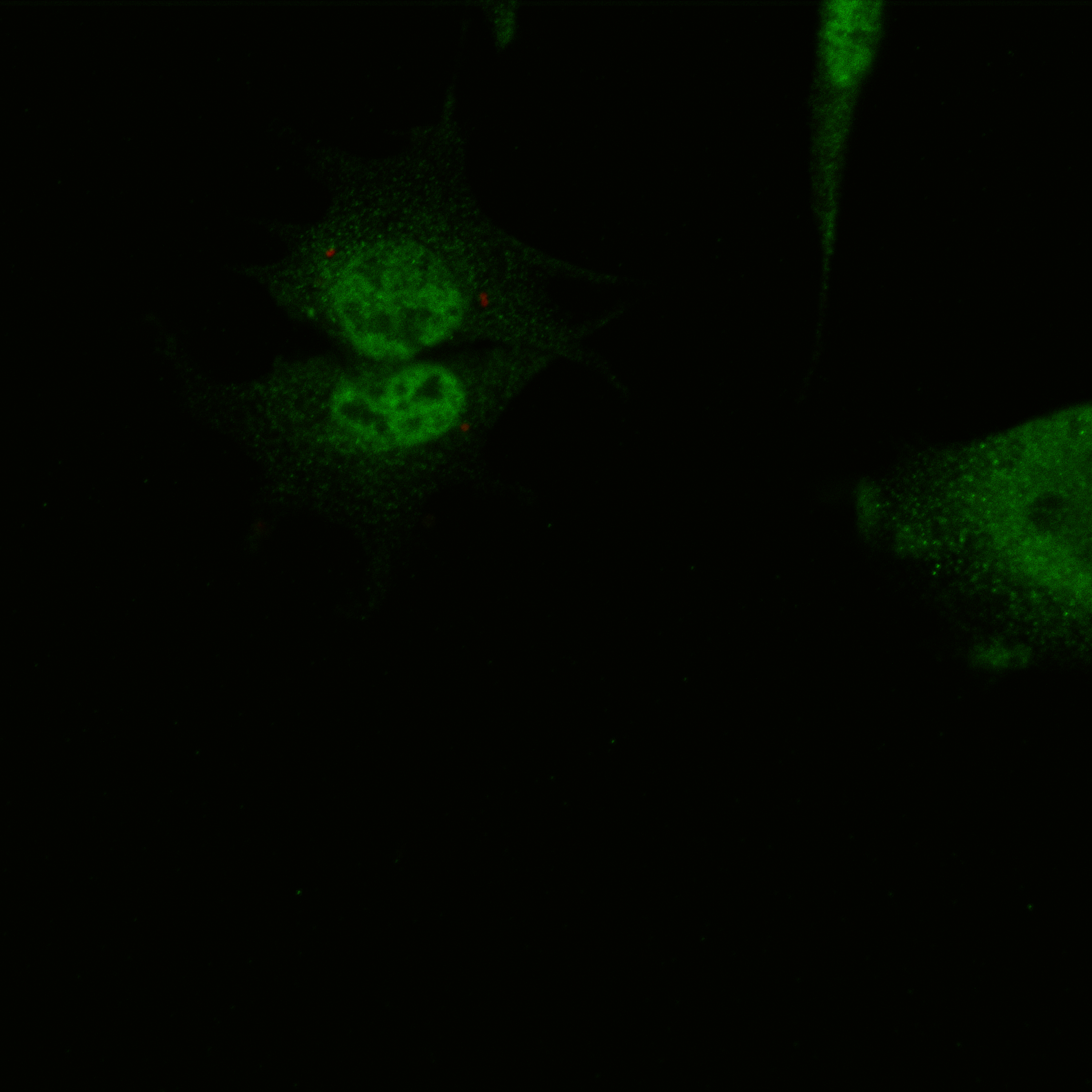

Supplement: Supplementary file 10 — Source Data for Figure 5 [file EMBJ-42-e113012-s010.zip › Figure 5/5A/Figure 5A_panel4.tif]

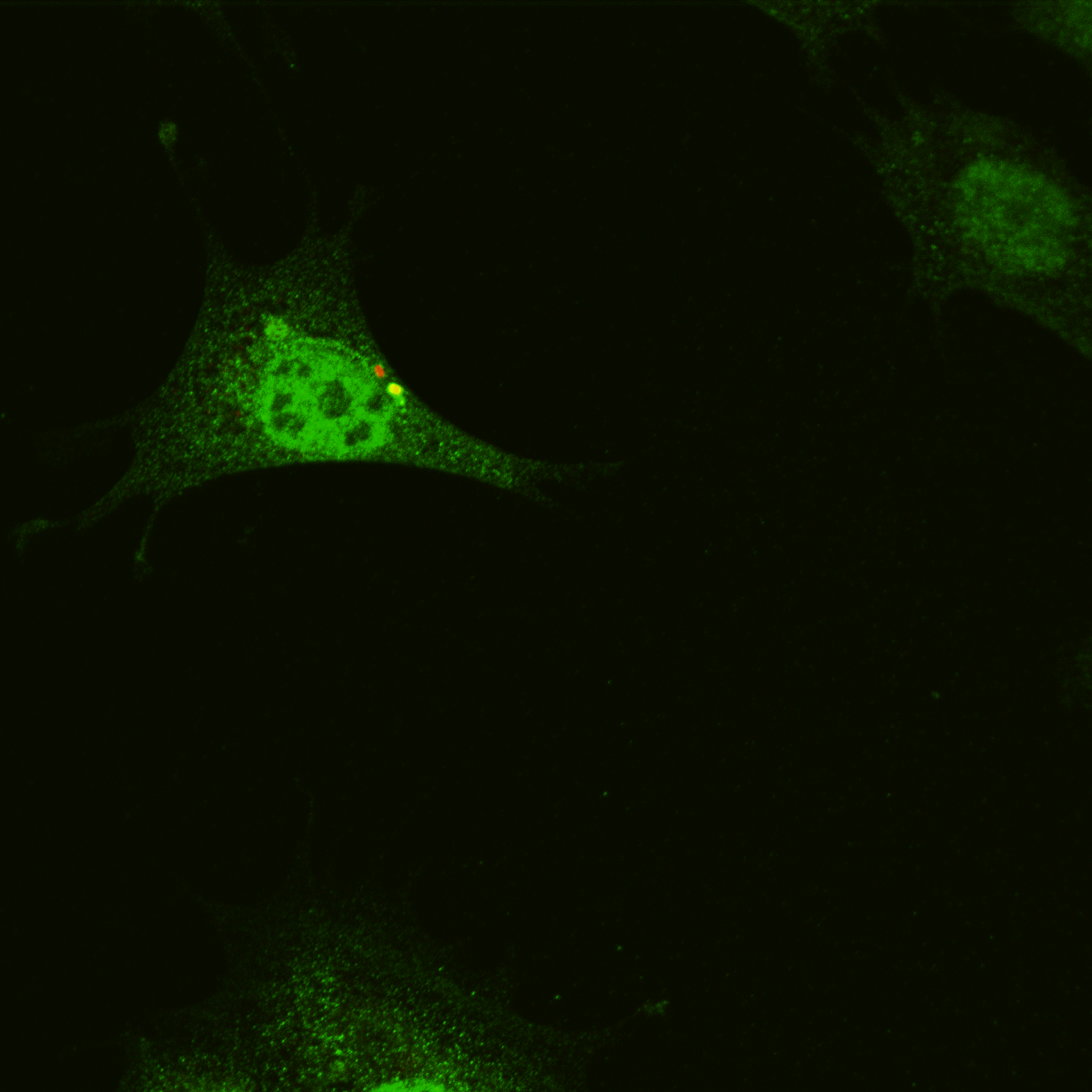

Supplement: Supplementary file 10 — Source Data for Figure 5 [file EMBJ-42-e113012-s010.zip › Figure 5/5A/Figure 5A_panel5.tif]

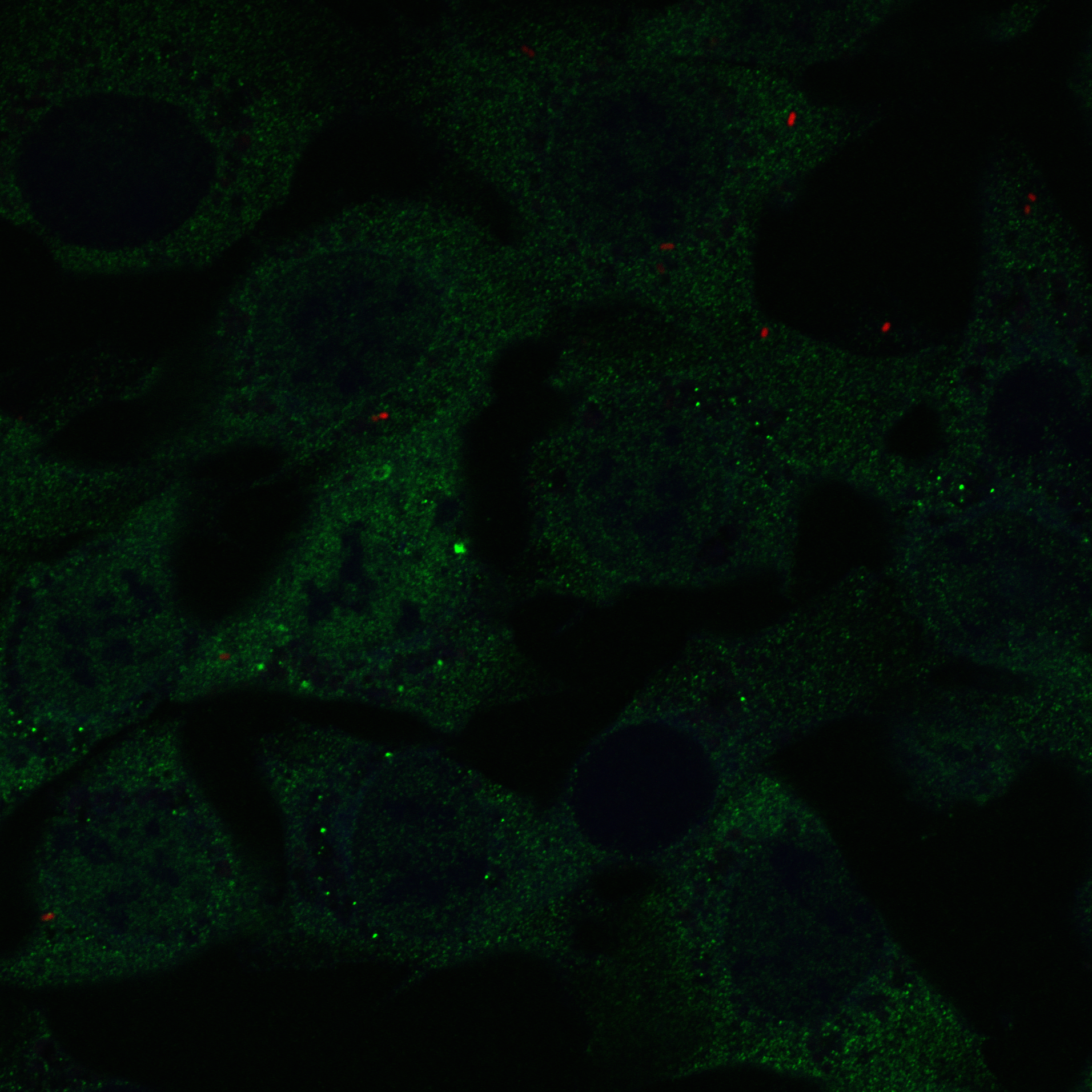

Supplement: Supplementary file 10 — Source Data for Figure 5 [file EMBJ-42-e113012-s010.zip › Figure 5/5F/Figure 5F_panel4.tif]

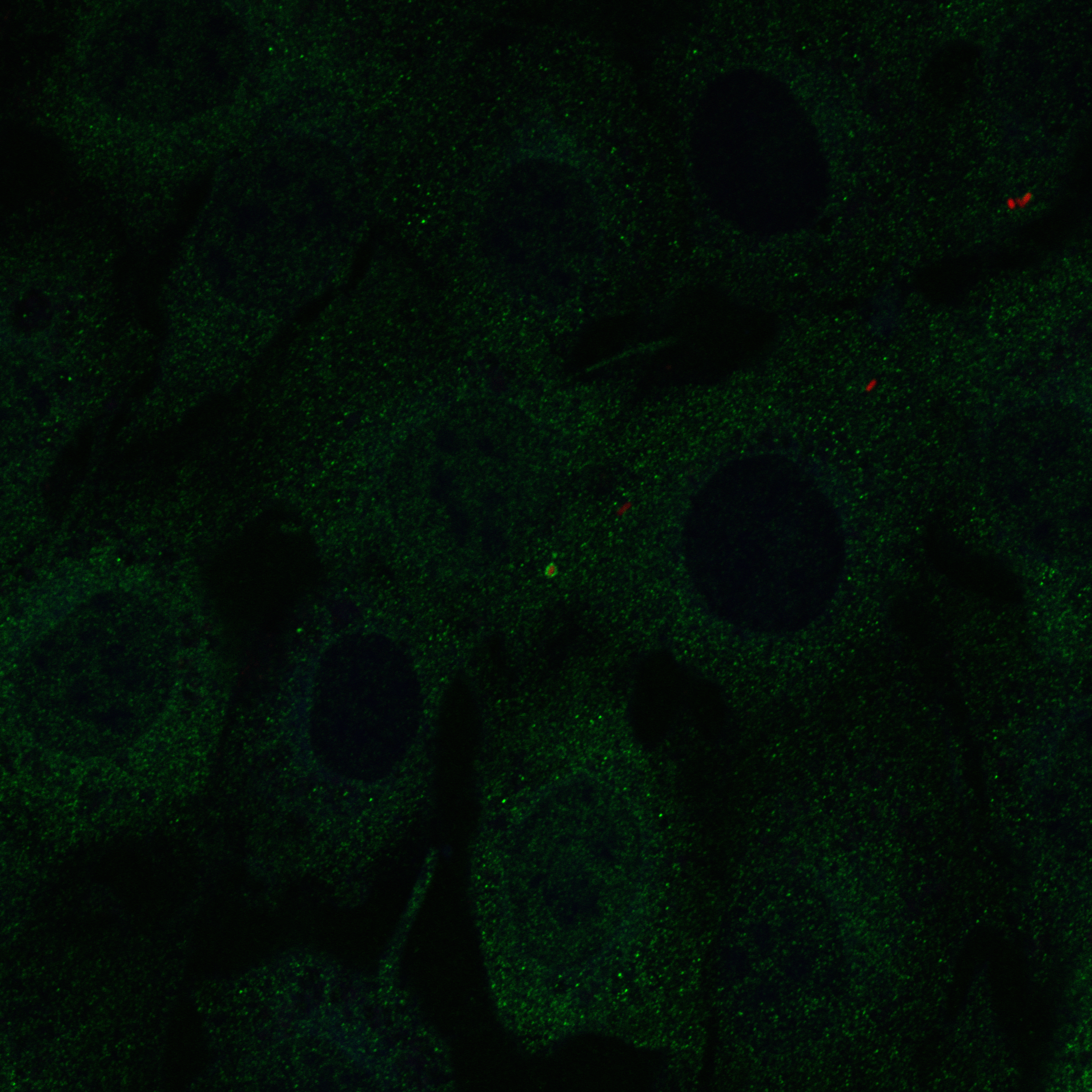

Supplement: Supplementary file 10 — Source Data for Figure 5 [file EMBJ-42-e113012-s010.zip › Figure 5/5F/Figure 5F_panel3.tif]

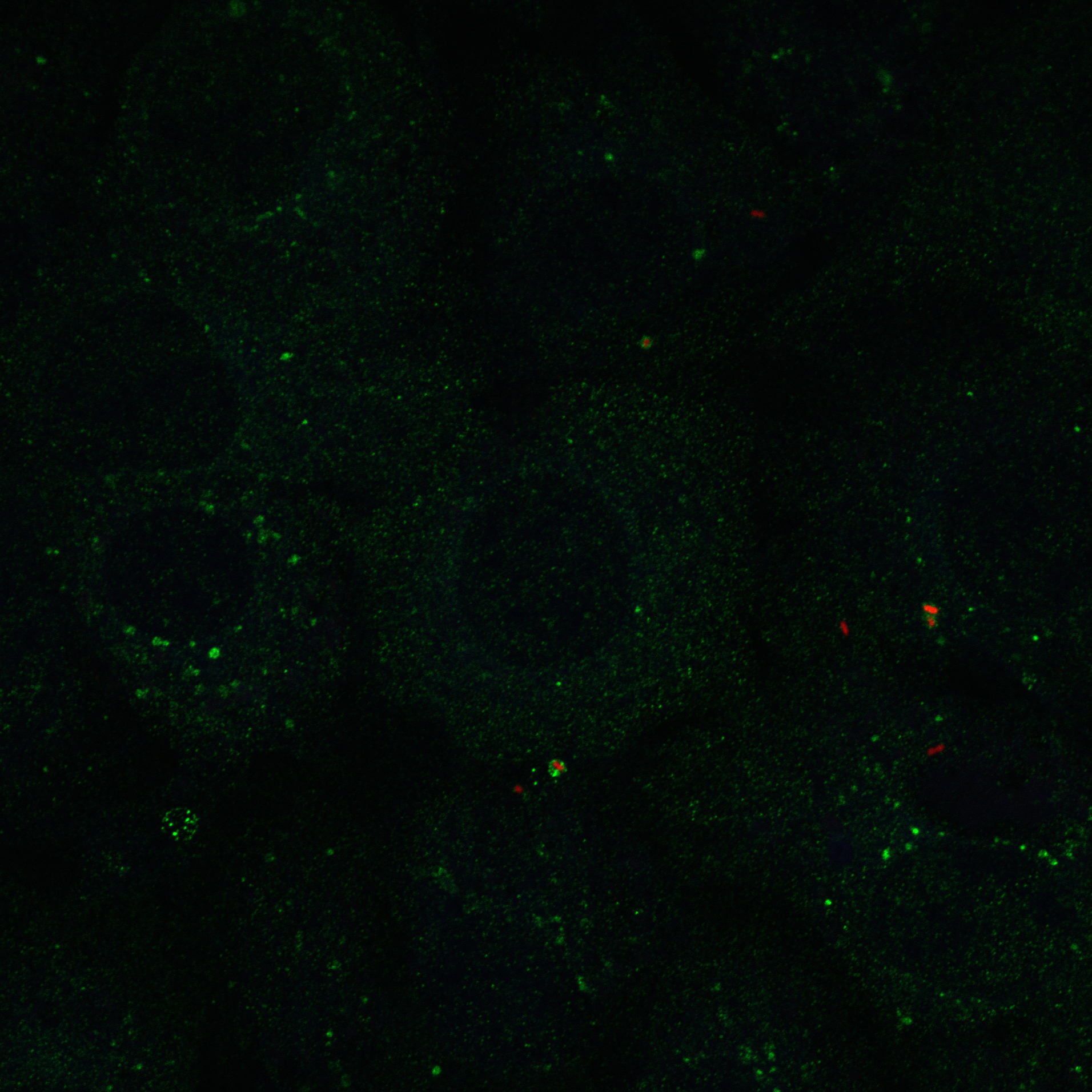

Supplement: Supplementary file 10 — Source Data for Figure 5 [file EMBJ-42-e113012-s010.zip › Figure 5/5F/Figure 5F_panel2.tif]

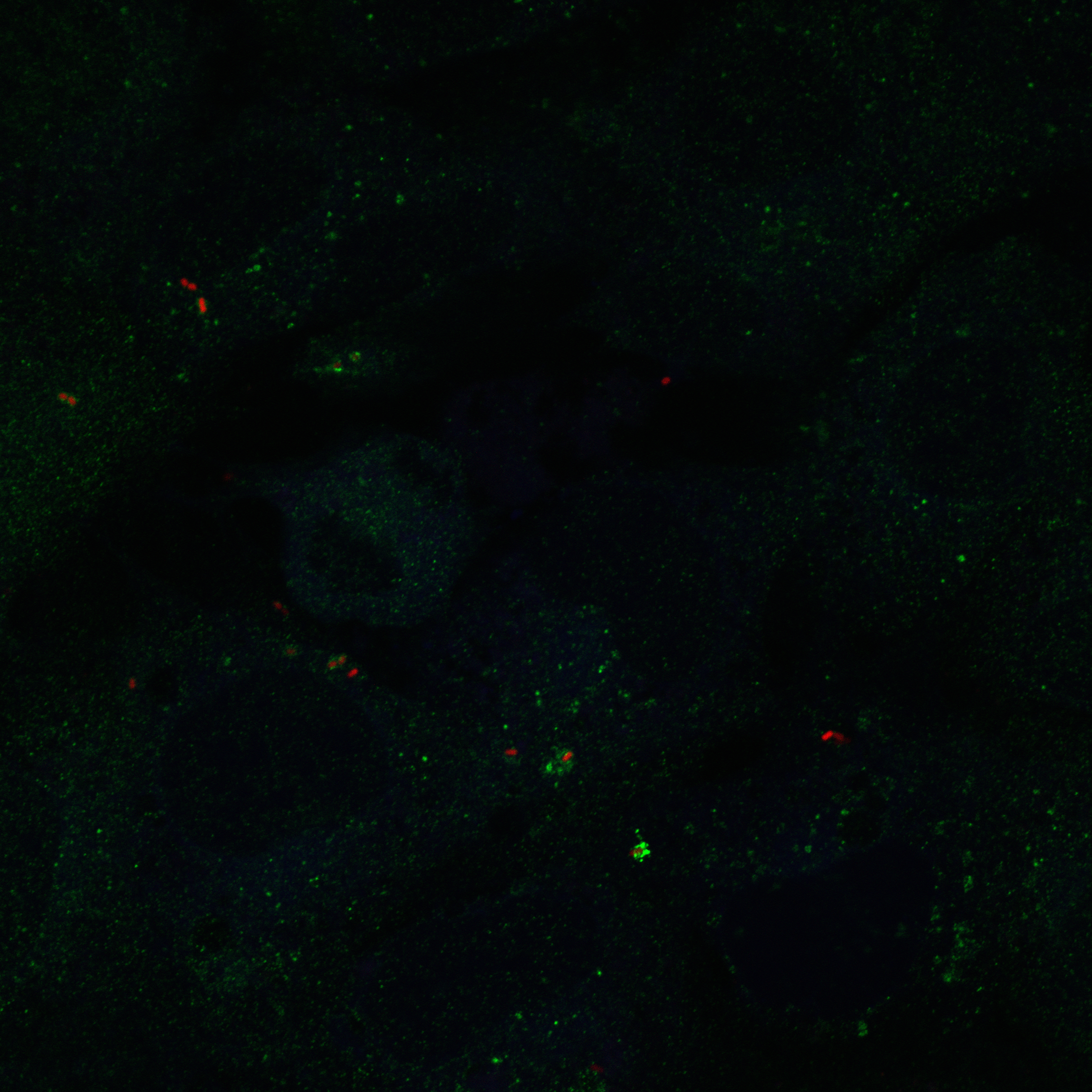

Supplement: Supplementary file 10 — Source Data for Figure 5 [file EMBJ-42-e113012-s010.zip › Figure 5/5F/Figure 5F_panel1.tif]

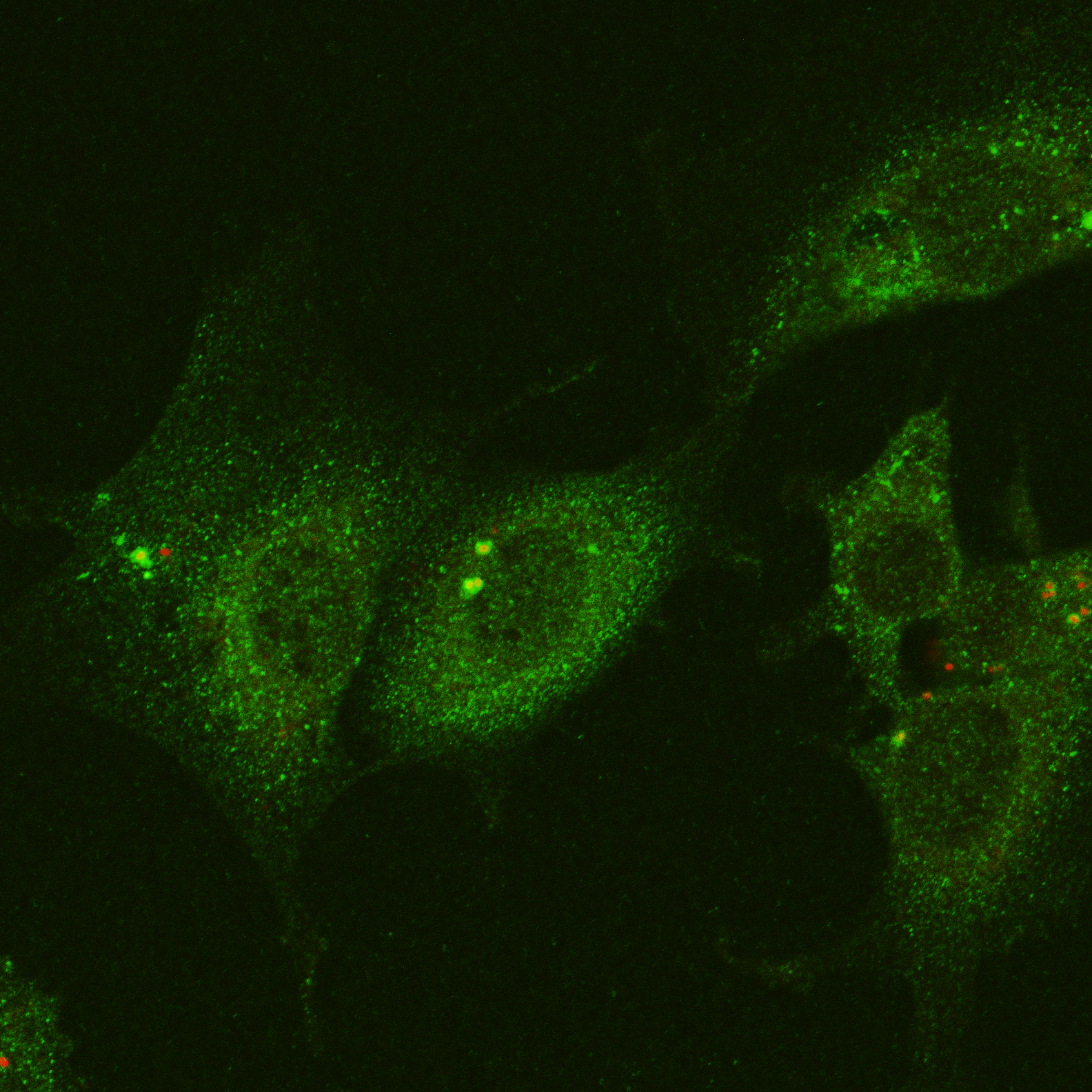

Supplement: Supplementary file 10 — Source Data for Figure 5 [file EMBJ-42-e113012-s010.zip › Figure 5/5C/Figure 5C_panel3.tif]

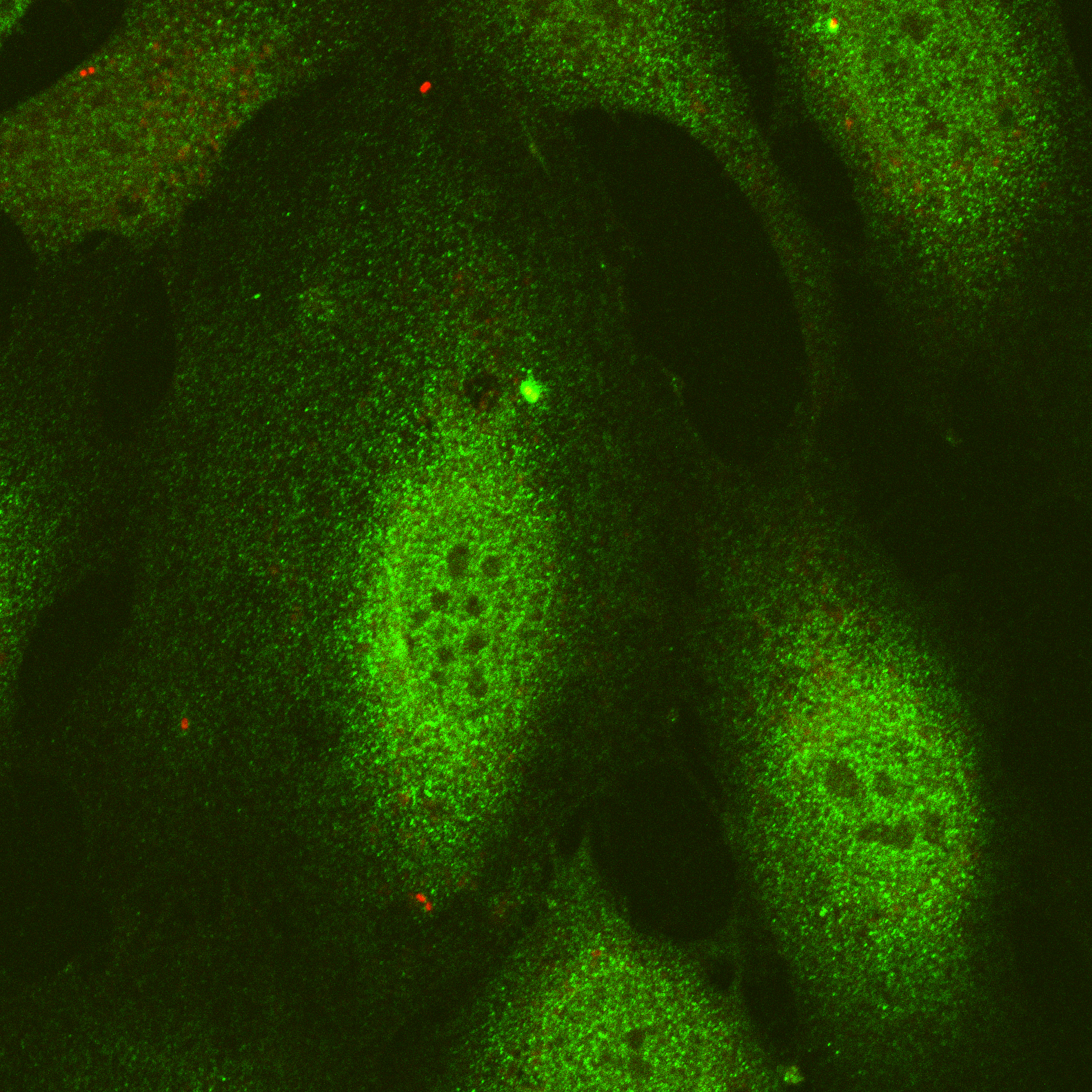

Supplement: Supplementary file 10 — Source Data for Figure 5 [file EMBJ-42-e113012-s010.zip › Figure 5/5C/Figure 5C_panel 2.tif]

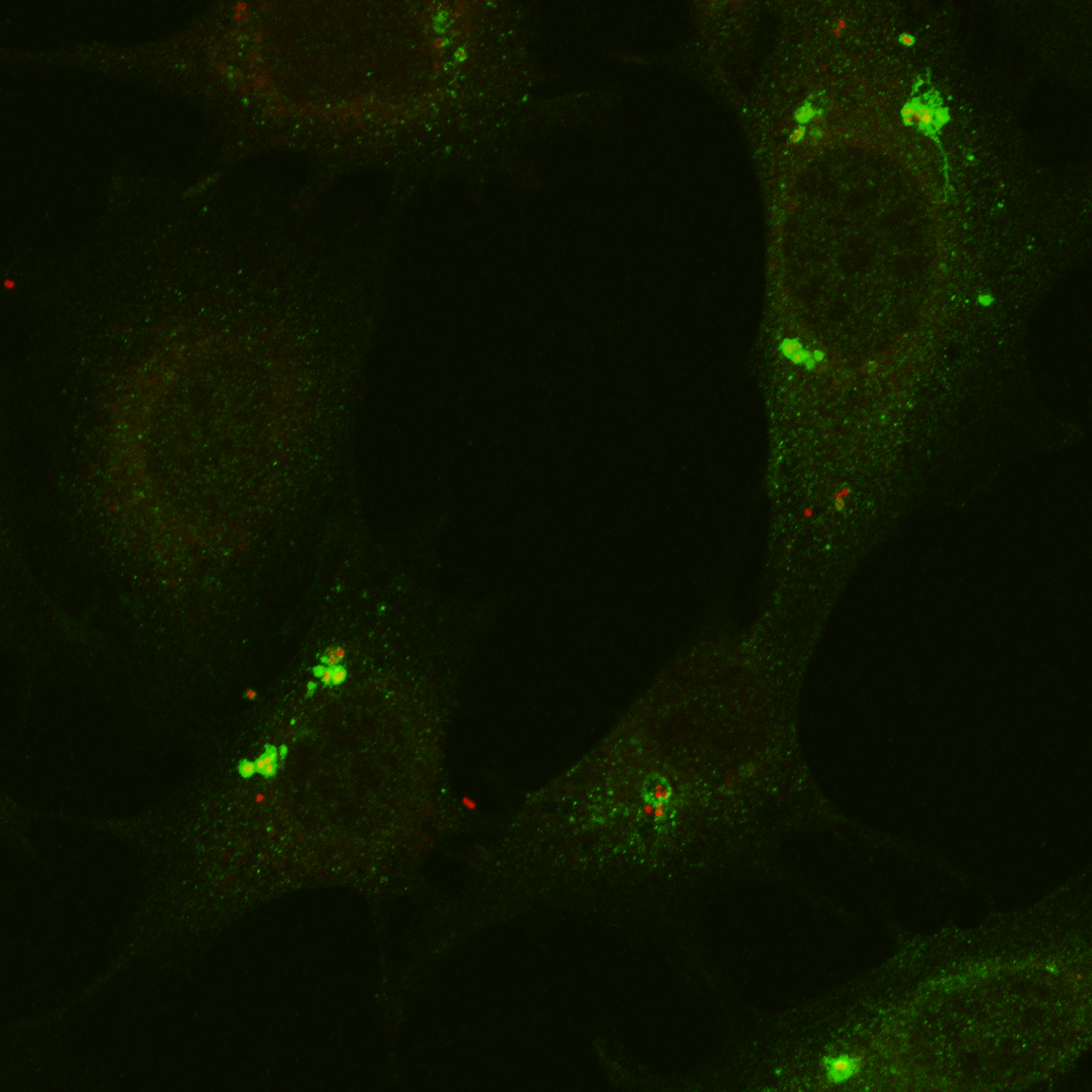

Supplement: Supplementary file 10 — Source Data for Figure 5 [file EMBJ-42-e113012-s010.zip › Figure 5/5C/Figure 5C_panel 1.tif]

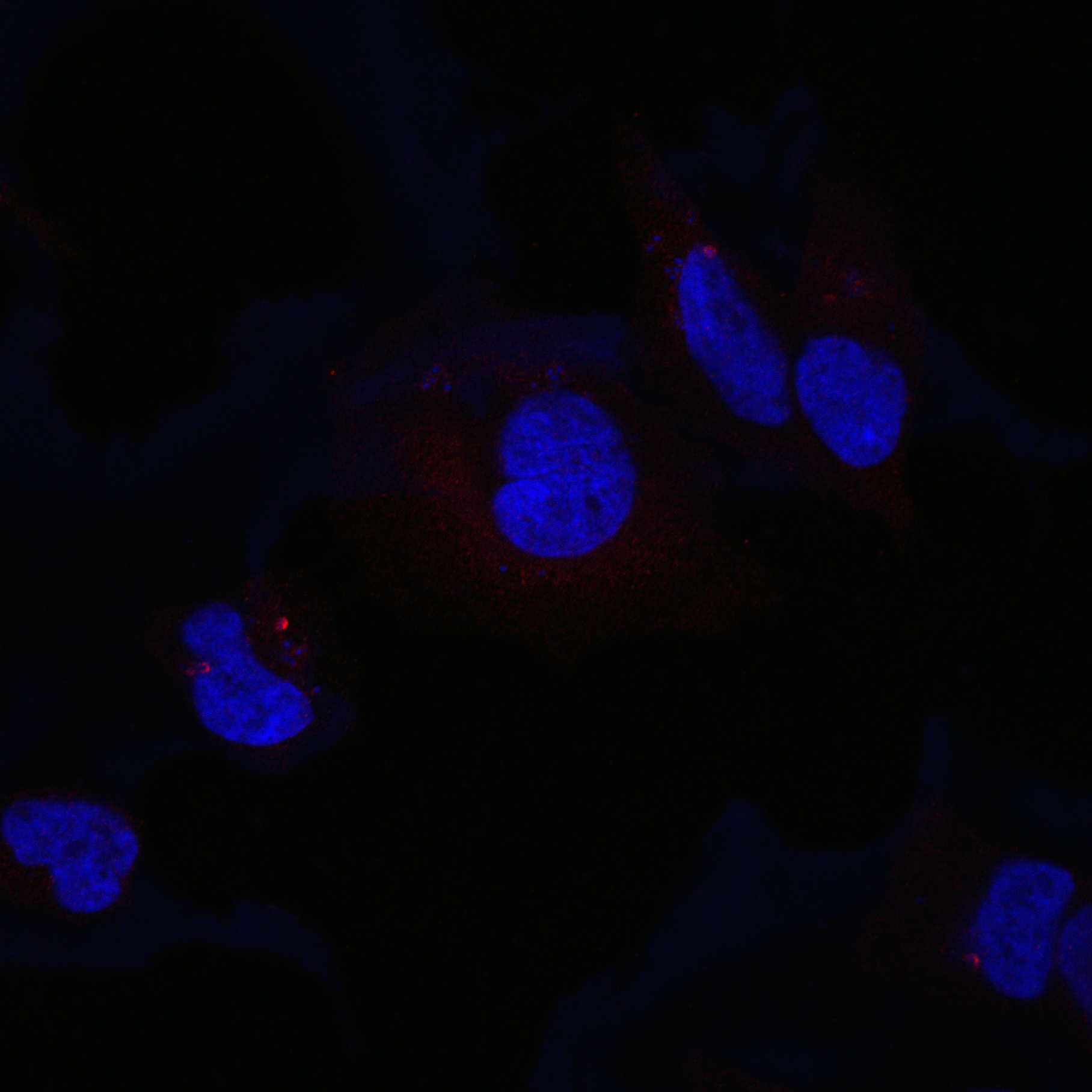

Supplement: Supplementary file 11 — Source Data for Figure 6 [file EMBJ-42-e113012-s007.zip › Figure 6/6B/Figure 6B_top.tif]

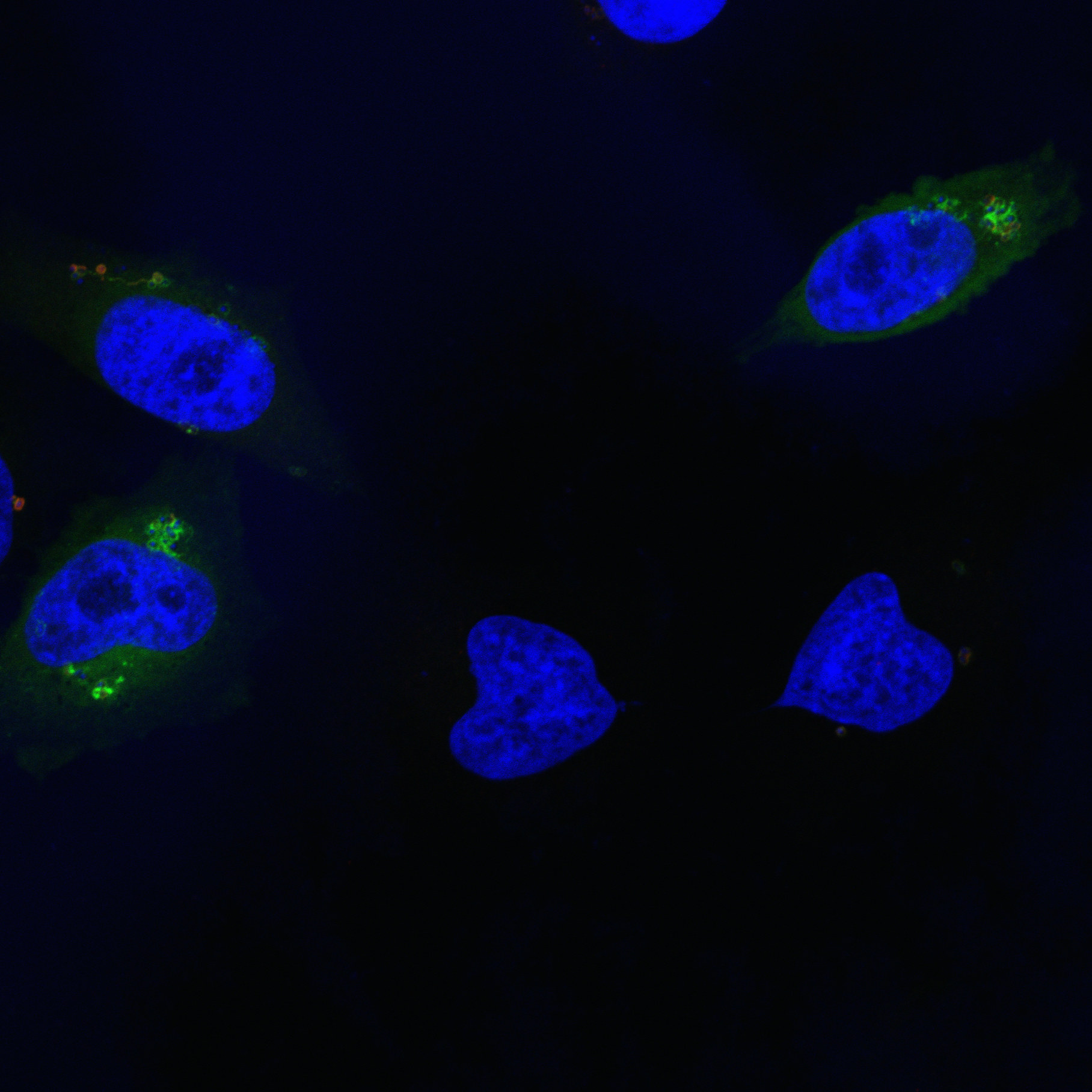

Supplement: Supplementary file 11 — Source Data for Figure 6 [file EMBJ-42-e113012-s007.zip › Figure 6/6B/Figure 6B_middle.tif]

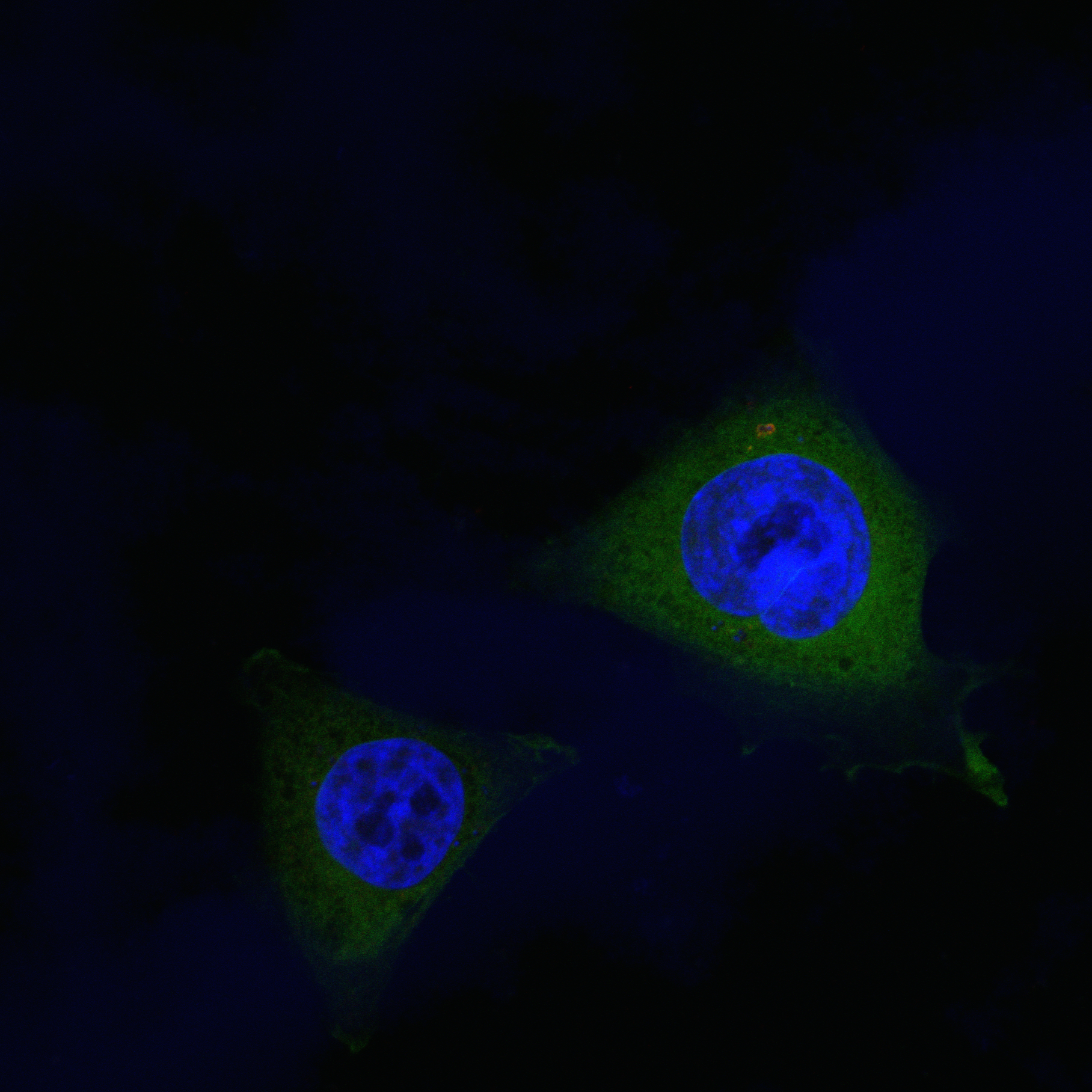

Supplement: Supplementary file 11 — Source Data for Figure 6 [file EMBJ-42-e113012-s007.zip › Figure 6/6B/Figure 6B_bottom.tif]
