# Supplementary material for: TECPR1 conjugates LC3 to damaged endomembranes upon detection of sphingomyelin exposure
Source: EMBO J. 2023 Jul 6;42(17):e113012. doi: 10.15252/embj.2022113012 (PMC10476172; doi:10.15252/embj.2022113012)
Supplement: Supplementary file 7 — Source Data for Figure 2 [file EMBJ-42-e113012-s003.zip › Figure 2/2G/2G README.rtf]

Figure 2G_panel 1 is original uncropped image of N’ DysFFigure 2G_panel 2 is original uncropped image of C’ DysF rotated by 180 degreesFigure 2G_panel 3 is original uncropped image of Myoferlin DysFFigure 2G_panel 4 is original uncropped image of Dysferlin DysF rotated by 180 degreesFigure 2G_panel 5 is original uncropped image of Fer1l5 DysF 
