# Supplementary material for: TECPR1 conjugates LC3 to damaged endomembranes upon detection of sphingomyelin exposure
Source: EMBO J. 2023 Jul 6;42(17):e113012. doi: 10.15252/embj.2022113012 (PMC10476172; doi:10.15252/embj.2022113012)
Supplement: Supplementary file 7 — Source Data for Figure 2 [file EMBJ-42-e113012-s003.zip › Figure 2/2E/2E README.rtf]

Figure 2E_top is is original uncropped image of N’ DysF control rotated by 90 degreesFigure 2E_bottom is is original uncropped image of N’ DysF + nSMase 
