# Supplementary material for: TECPR1 conjugates LC3 to damaged endomembranes upon detection of sphingomyelin exposure
Source: EMBO J. 2023 Jul 6;42(17):e113012. doi: 10.15252/embj.2022113012 (PMC10476172; doi:10.15252/embj.2022113012)
Supplement: Supplementary file 8 — Source Data for Figure 3 [file EMBJ-42-e113012-s011.zip › Figure 3/3F/3F README.rtf]

Figure 3F_top left is original uncropped image of TECPR1 WT, DMSO with DAPI channel includedFigure 3F_top right is original uncropped image of TECPR1 WT, LLOMe with DAPI channel includedFigure 3F_bottom left is original uncropped image of TECPR1 W154A, DMSO with DAPI channel includedFigure 3F_bottom right is original uncropped image of TECPR1 W154A, LLOMe with DAPI channel included
