# Supplementary material for: TECPR1 conjugates LC3 to damaged endomembranes upon detection of sphingomyelin exposure
Source: EMBO J. 2023 Jul 6;42(17):e113012. doi: 10.15252/embj.2022113012 (PMC10476172; doi:10.15252/embj.2022113012)
Supplement: Supplementary file 10 — Source Data for Figure 5 [file EMBJ-42-e113012-s010.zip › Figure 5/5A/5A README.rtf]

Figure 5A_panel1 is original uncropped image of controlFigure 5A_panel2 is original uncropped image of ATG5KOFigure 5A_panel3 is original uncropped image of ATG16L1 KO rotated by 180 degreesFigure 5A_panel4 is original uncropped image of TECPR1 KOFigure 5A_panel5 is original uncropped image of TECPR1 KO +TECPR1
