# Supplementary material for: TECPR1 conjugates LC3 to damaged endomembranes upon detection of sphingomyelin exposure
Source: EMBO J. 2023 Jul 6;42(17):e113012. doi: 10.15252/embj.2022113012 (PMC10476172; doi:10.15252/embj.2022113012)
Supplement: Supplementary file 10 — Source Data for Figure 5 [file EMBJ-42-e113012-s010.zip › Figure 5/5F/5F README.rtf]

Figure 5F_panel 1 is original uncropped image of control cells, siControlFigure 5F_panel 2 is original uncropped image of control cells, siTECPR1Figure 5F_panel 3 is original uncropped image of ATG16L1 KO cells, siControl rotated by 90 degreesFigure 5F_panel 4 is original uncropped image of ATG16L1 KO cells, siTECPR1 rotated by 90 degrees
