# Supplementary material for: TECPR1 conjugates LC3 to damaged endomembranes upon detection of sphingomyelin exposure
Source: EMBO J. 2023 Jul 6;42(17):e113012. doi: 10.15252/embj.2022113012 (PMC10476172; doi:10.15252/embj.2022113012)
Supplement: Supplementary file 10 — Source Data for Figure 5 [file EMBJ-42-e113012-s010.zip › Figure 5/5C/5C README.rtf]

Figure 5C_panel 1 is original uncropped image of controlFigure 5C_panel 2 is original uncropped image of ATG16L1 KO rotated by 180 degreesFigure 5C_panel 3 is original uncropped image of TECPR1 KO
