# Supplementary material for: TECPR1 conjugates LC3 to damaged endomembranes upon detection of sphingomyelin exposure
Source: EMBO J. 2023 Jul 6;42(17):e113012. doi: 10.15252/embj.2022113012 (PMC10476172; doi:10.15252/embj.2022113012)
Supplement: Supplementary file 11 — Source Data for Figure 6 [file EMBJ-42-e113012-s007.zip › Figure 6/6B/6B README.rtf]

Figure 6B_top is original uncropped image of controlFigure 6B_middle of original uncropped image of TECPR1 WTFigure 6B_bottom is original uncropped image of TECPR1 W154A
