# Supplementary material for: TECPR1 conjugates LC3 to damaged endomembranes upon detection of sphingomyelin exposure
Source: EMBO J. 2023 Jul 6;42(17):e113012. doi: 10.15252/embj.2022113012 (PMC10476172; doi:10.15252/embj.2022113012)
Supplement: Supplementary file 12 [file EMBJ-42-e113012-s009.pdf]

THE EMBO JOURNAL

TECPR1 conjugates LC3 to damaged endomembranes upon detection of sphingomyelin exposure

Keith Boyle, Cara Ellison, Paul Elliott, Martina Schuschnig, Krista Grimes, Marc Dionne, Chihiro Sasakawa, Sean Munro, Sascha Martens and Felix Randow
DOI: 10.15252/embj.2022113012

Corresponding author(s): Felix Randow (randow@mrc-lmb.cam.ac.uk)

Review Timeline:

Submission Date:	9th Nov 22
Editorial Decision:	10th Jan 23
Revision Received:	26th Apr 23
Editorial Decision:	18th May 23
Revision Received:	7th Jun 23
Accepted:	14th Jun 23

Editor: Karin Dumstrei

Transaction Report:

(Note: With the exception of the correction of typographical or spelling errors that could be a source of ambiguity, letters and reports are not edited. Depending on transfer agreements, referee reports obtained elsewhere may or may not be included in this compilation. Referee reports are anonymous unless the Referee chooses to sign their reports.)

1st Editorial Decision 10th Jan 2023

Dear Felix,

Thank you for submitting your manuscript to The EMBO Journal. I am sorry for the slight delay in getting back to you, but I have now received the three reports on the manuscript.

As you can see the referees appreciate the findings reported and find the analysis insightful. They raise several issues that I would like to ask you to address in a revised version.

I think it would be helpful to discuss the raised points further and I am available to do so via email or video.

When preparing your letter of response to the referees' comments, please bear in mind that this will form part of the Review Process File, and will therefore be available online to the community. For more details on our Transparent Editorial Process, please visit our website: https://www.embopress.org/page/journal/14602075/authorguide#transparentprocess

Thank you for the opportunity to consider your work for publication. I look forward to your revision.

with best wishes

Karin

Karin Dumstrei, PhD
Senior Editor
The EMBO Journal

Instructions for preparing your revised manuscript:

Please make sure you upload a letter of response to the referees' comments together with the revised manuscript.

Please also check that the title and abstract of the manuscript are brief, yet explicit, even to non-specialists.

When assembling figures, please refer to our figure preparation guideline in order to ensure proper formatting and readability in print as well as on screen:
https://bit.ly/EMBOPressFigurePreparationGuideline
See also guidelines for figure legends: https://www.embopress.org/page/journal/14602075/authorguide#figureformat

At EMBO Press we ask authors to provide source data for the main manuscript figures. Our source data coordinator will contact you to discuss which figure panels we would need source data for and will also provide you with helpful tips on how to upload and organize the files.

IMPORTANT: When you send the revision we will require
- a point-by-point response to the referees' comments, with a detailed description of the changes made (as a word file).
- a word file of the manuscript text.
- individual production quality figure files (one file per figure)
- a complete author checklist, which you can download from our author guidelines (https://www.embopress.org/page/journal/14602075/authorguide).
- Expanded View files (replacing Supplementary Information)
Please see out instructions to authors
https://www.embopress.org/page/journal/14602075/authorguide#expandedview

Please remember: Digital image enhancement is acceptable practice, as long as it accurately represents the original data and conforms to community standards. If a figure has been subjected to significant electronic manipulation, this must be noted in the figure legend or in the 'Materials and Methods' section. The editors reserve the right to request original versions of figures and the original images that were used to assemble the figure.

Further information is available in our Guide For Authors: https://www.embopress.org/page/journal/14602075/authorguide

We realize that it is difficult to revise to a specific deadline. In the interest of protecting the conceptual advance provided by the work, we recommend a revision within 3 months (10th Apr 2023).

As a matter of policy, competing manuscripts published during this period will not negatively impact on our assessment of the conceptual advance presented by your study. However, we request that you contact the editor as soon as possible upon publication of any related work, to discuss how to proceed. Should you foresee a problem in meeting this three-month deadline, please let us know in advance as we can grant an extension.

Referee #1:

The authors of this manuscript studied a host defense system that detects damaged bacteria containing vacuoles (BCV) using mainly Salmonella and for some experiments Shigella and Listeria. Based on previous observations that sphingomyelin (SM) is asymmetrically distributed by strictly localizing to the extracellular leaflet of the plasma membrane or the luminal leaflet of lysosomes, the authors applied a mass-spec approach to identify potential SM sensing proteins. They found that TECPR1, know to be targeted to lysosomal membranes (PMIDs 22342342 and 32532970), binds SM through its N-terminal DysF domain. They also found that TECPR1 recruits ATG5 and LC3 to Salmonella, suggesting that TECPR1 promotes LC3 lipidation. An ATG5 dependent function of TECPR1 in clearance of intracellular bacteria by selective autophagy has been described before (PMID 21575909). However, TECPR1 was found to be targeted to cytoplasmic Shigella and the mechanism by which autophagy was induced remained largely uncharacterized. Here, the authors describe a new mechanism by which host cells sense damaged BCVs and induce conjugation of LC3 to activate a noncanonical autophagy pathway. Although this is a very interesting study, data that are supporting the central claim of the authors that TECPR1 recognizes SM through its DysF domain, raptures SCVs and promotes LC3 recruitment in an ATG5 dependent manner are not entirely convincing. Several major concerns would need to be addressed before a recommendation concerning publication of the manuscript can be made.

Major:

1) The authors used for their experiments mostly the intracellular pathogens Salmonella and Shigella. The majority of Salmonella remains in SCVs, while Shigella gains rapidly access to the cytoplasm by lysing the BCV. Targeting of TECPR1 to BCVs thus need to be rapid and induction of autophagy must occur very fast, before bacteria can escape. Based on the data presented in this manuscript, it remains unclear whether bacteria already escaped BCVs, are associated with BCV remnants, or are trapped in partially lysed or intact BCVs. This concern also applies to experiments with Salmonella. The authors need to establish the integrity of the BCV membrane more carefully by using e.g. electron microscopy. This is critical because the authors claim that the TECPR1 mediated response precedes Gal8 mediated induction of autophagy. The latte mechanism is well-established and known to detect severe damage of BCVs. Along these lines, it is unclear why >30% of Shigella remain associated with BCVs 30 min post infection (Fig 1F), where most of bacteria should have escaped BCVs. If the recruitment of TECPR1 occurs before rupture of the BCVs, why do the author observe colocalization of TECPR1 late (peak at 1 h p.i.)?
2) The authors found that expression of SMPD3 impaired TECPR1 recruitment to BCVs (Fig 1H). However, the representative images are not very convincing since bacteria were detected by DAPI staining and in most cases, bacteria are in immediate vicinity to the nucleus. In all other experiments, fluorescent labeled bacteria were used. The authors need to repeat these experiments using fluorescent bacteria and combine them with CLEM to establish the morphology of the BCV.
3) Along the same lines, the authors reported that the DysF domain of TECPR1 recognizes SM based on it colocalization with BCVs in wt and nSMase2 expressing cells. These data suffer from the same limitation, given that bacteria are again detected by DAPI staining. In corresponding experiments without expression of nSMase2, fluorescent bacteria have been used (Fig. 2G) but it is not clear whether bacteria are trapped in SCVs since the image show single Salmonella, most of which are not associated with DysF (Fig 2G).
4) The authors validated binding of DysF to SM using liposome floatation assays. The accessibility of exposed surfaces of this domain in context of the full length protein remains, however, uncertain and TECRP1 full length was not included as control. The authors should repeat these experiments using full length TECPR1 and its corresponding mutant. Moreover, there is a conceptional problem with these experiments: LUVs are intact, sphingosine will thus be buried in the bilayer core and not be exposed. The interaction of DysF with these LUVs might be nonspecific, given that SM are positively charged. It is thus likely that the interaction of DysF and SM containing liposomes is not driven by hydrophobic interactions of residues of DysF with SM, but by charged interaction. The authors need to increase the salt concentration of the buffer to exclude that charged interactions are responsible. The authors should also perform experiments with liposomes containing NBD-PE, which is a probe that changes fluorescence based on the hydrophobicity of its environment to reveal whether lipids are exposed upon incubation with DysF, TECPR1 or corresponding mutants.
5) Given the above-mentioned limitations of the characterization of DysF, the authors should show representative images for their quantification of the recruitment of DysF mutants to Salmonella (Fig 3B and C). Moreover, it remains unclear whether exposed hydrophobic residues in the DysF domain, including W154 and W77, are also exposed in full length TECPR1. This can be evaluated by e.g. HD exchange experiments.
6) The result concerning LC3 lipidation/recruitment to Salmonella are puzzling. The authors found that in TECPR1 KO cells,

ATG5 is not recruited to bacteria (Fig 4B). However, LC3C is still recruited to Salmonella (Fig. 4D) and the authors concluded that this recruitment occurs in an ATG16L1 dependent manner (Fig 4G). However, if ATG16L1 is not in complex with ATG12-ATG5, it cannot lipidate LC3. It is thus unclear how ATG16L1 promotes LC3 lipidation if there is no recruitment of ATG5 to Salmonella upon deletion of TECPR1. The authors should compare LC3 recruitment to Salmonella and to autophagosomes/phagophores in canonical autophagy (as internal control) to resolve this contradiction. Moreover, previous studies demonstrated that KO of TECPR1 did not affect LC3 lipidation in autophagy, while lipidation was abrogated in ATG16L1 KO cells, arguing that ATG16L1 mediated lipidation dominates.
7) The authors showed that in cells overexpressing TECPR1, more Salmonella were Gal8 positive and concluded that TECPR1 induced membrane damage. However, the enhanced recruitment of Gal8 upon TECPR1 overexpression is not a proof of membrane damage by TECPR1. Also, the observation that W154A and delta PH lead to a reduced colocalization of Salmonella and Gal8 is not a proof of membrane damage. Moreover, it is puzzling why TECPR1 recruitment to Salmonella is supposed to induce antibacterial, selective autophagy, given that expression of TECPR1 leads to an enhanced growth of bacteria (Fig 5D). One would expect the opposite effect.

Minor:

1) Previous studies demonstrated that TECPR1 is localized to lysosomal compartments based on its PH domain. Here, the authors found that TECPR1 is mostly cytoplasmic and puncta colocalized to endosomal compartments. How do the authors explain these differences?
2) The authors stated on page 8 line 195 that recruitment of full-length TECPR1 to SCVs peaked at 1 h p.i. (Fig. 2B), However, no time dependent recruitment of TECPR1 is shown in this figure (only shown in the plot in Fig. 2C). Representative images of full-length TECPR1 should be added.

Referee #2:

Boyle et al., identified that TECPR1 as a cytosolic sensor for sphingomyelin. They showed that TECPR1 bound to sphingomyelin through N-terminal DysF domain. By crystal structural analysis, they also identified key residue for the interaction. Interestingly, TECPR1 function as atypical E3 ligase together with ATG5 and mediated non-canonical lipidation of LC3 independently of ATG16L1. Although the identification of TECRP1 as cytosolic receptor of sphingomyelin and alternative E3 for LC3 lipidation is interesting, the fundamental question why this system is required need to be experimentally solved.

Major

1. Most importantly, authors need to show the biological significance of TECPR1 mediated LC3 lipidation. If loss of TECPR1 does not affect proliferation of S. Typh. compared to ATG16L1, why does this system exist?

2. It seems not enough to propose that TECPR1 mediates LC3 lipidation to damaged endosomes because the results could not dissect canonical and non-canonical lipidation. The authors need to analyze the effect of TECPR1 depletion on LC3 recruitment in canonical autophagy-deficient background such as FIP200 KO.

3. Is there any functional difference between ATG16L1 and TECPR1 dependent LC3 lipidation? Does TECPR1 preferentially mediate lipidation of specific ATG8 subfamilies? It is also worth to examine the effect on the bacteria growth when TECPR1 is knocked down or overexpressed in ATG16L1 KO.

4. Does any other stress such as LLOMe, Monensin, GPN induce TECPR1 recruitment on endo-lysosomal membrane? If so, is DysF domain required?

5. Non-canonical lipidation of LC3 on endo-lysosomal membrane is involved in several biological processes including LAP (Martinez et al., 2015), LANDO (Heckmann et al., 2019), selective lysosome membrane turnover (Lee et al., 2020) and TFEB activation (Goodwin et al., 2021; Nakamura et al., 2020). Is TECPR required for any of these processes?

6. When TECPR1(ΔPH) is overexpressed, does TECPR1 accumulate in the damaged membrane? If so, does LC3 also accumulate?

7. Why is LC3 accumulation on SCVs not suppressed in TECPR1 KO cells even though recruitment of ATG5 is completely suppressed? In page 16, line 382 to 384, the authors suggested "ATG16L1-dependent lipidation of LC3 occurring at locations distant from the damaged vacuole. In contrast, it suggests that TECPR1 facilitates LC3 lipidation in the immediate vicinity of the sphingomyelin-positive structure". Which evidence supports this suggestion?

8. Is Fig.4E, are comparison between DMSO and Wortmannin significantly different?

9. What do the authors intend to suggest from the results in Figure 5?

Minor
1. Line 144, the reference is missing.

2. Does the deletion N terminal DysF reduce the recruitment of TECPR1 to S. Typh.?

3. In Figure 5D, is LC3 recruitment in WT cells comparable to TECPR1 KO cells? Please show the result of WT cell like Figure 5A and 5B.

4. Even though it is shown that TECPR1 recruitment on BEVs is suppressed by ectopic expression of nSMase2, the authors need to show the correlation of sphingomyelin exposure to cytosol and TECPR1 recruitment on BCVs.

5. Please include what the authors found in the last part of introduction.

Referee #3:

Boyle et al provide a very interesting study which proposes a novel mechanism that cells employ to sense endomembrane stress upon damage, especially during invasive bacterial infection. Authors report that upon membrane damage, sphingomyelin is exposed on the cytosolic leave of the vacuole which is detected by the protein TECPR1, which binds SM and is recruited to the membrane, with authors solving the crystal structure of the domin in TECPR1 requires for SM interaction. TECPR1 then interacts with the autophagy proteins ATG5-12 complex and directs the conjugation of ATG8/LC3 to the membrane. This ATG8 response is part of the non-canonical autophagy/CASM pathway. Importantly, and surprisingly, authors show that part of the ATG8 lipidation is independent of ATG16L1, which has previously been shown to direct the membrane site of lipidation in non-canonical autophagy in conjunction with the v-ATPase. Instead, the TECPR1-ATG5 axis can substitute, which provides a back up mechanism when pathogens evolve strategies to inhibit the ATG16L1-V-ATPase axis, such as SopF.
Authors also provide evidence that TECPR1 recruitment has the potential to exert further damage to membranes, potentially providing a mechanism to turn small membrane damage that is beneficial to the pathogen, to larger damages which can be targeted by the host.

This is a well executed study with many novel findings, including the expansion of the molecular machinery behind non-canonical autophagy. This work highlights previously unappreciated host cell responses. There are some comments that should be addressed.

Major points

1. Fig 4 has some interesting data, in which ATG5 recruitment to SVCs is completely blocked by TECPR1 KO. However, LC3 lipidation to SVCs is not affected, which the authors say is due to the redundancy between the TECPR1 and ATG16L1 pathways. On the face of it this is a confusing result, as both the pathways would require ATG5 for lipidation. Authors offer one explanation, that in the absence of TECPR1, ATG16L1 drives LC3 lipidation at a distant site which then traffics to the SVC. However, there is no evidence supplied to support this. If this was the case, there may be differences in the kinetics of LC3 recruitment to SVCs in WT and TECPR1 KO cells. Authors could examine this by timelapse microscopy. Further, trafficking or vesicle fusion could be impeded to see if this then blocks the LC3 lipidation in TECPR1 KO cells. Does ATG16L1 KO have any effect on ATG5 recruitment to the SVC?
As it stands, the explanation of this result is not strong; there is no suggestion as to what would regulate/control the ATG16L1 dependent lipidation at distant sites. This at least needs to be discussed more or experimentally investigated.
2. Fig5 shows data that TECPR1 can lead to increased membrane damage and Gal8 positivity and this is dependent on SM binding. This work was done with over-expresison of TECPR1. Do authors have any evidence that this occurs at physiological levels of expression (or have conditions where baseline levels are increased, does TECPR1 increase during infection)? Can authors see a reduction of Gal8 puncta in TECPR1 KO cells?
3. The strain of Salmonella used in the study would be expected to express SopF, and thus dampen the ATG16L1-V-ATPase axis of non-canonical autophagy, making it more likely that TECPR1 plays a functional role. Can authors comment on or use deltaSopF strains to determine whether when both pathways are active, which plays a more prominent role? Would TECPR1 KO have any effect on ATG5 recruitment under these conditions?

Minor point.

1. Many of the graphs are lacking statistical analysis

Referee #1:

The authors of this manuscript studied a host defense system that detects damaged bacteria containing vacuoles (BCV) using mainly Salmonella and for some experiments Shigella and Listeria. Based on previous observations that sphingomyelin (SM) is asymmetrically distributed by strictly localizing to the extracellular leaflet of the plasma membrane or the luminal leaflet of lysosomes, the authors applied a mass-spec approach to identify potential SM sensing proteins. They found that TECPR1, know to be targeted to lysosomal membranes (PMIDs 22342342 and 32532970), binds SM through its N-terminal DysF domain. They also found that TECPR1 recruits ATG5 and LC3 to Salmonella, suggesting that TECPR1 promotes LC3 lipidation. An ATG5 dependent function of TECPR1 in clearance of intracellular bacteria by selective autophagy has been described before (PMID 21575909). However, TECPR1 was found to be targeted to cytoplasmic Shigella and the mechanism by which autophagy was induced remained largely uncharacterized. Here, the authors describe a new mechanism by which host cells sense damaged BCVs and induce conjugation of LC3 to activate a noncanonical autophagy pathway. Although this is a very interesting study, data that are supporting the central claim of the authors that TECPR1 recognizes SM through its DysF domain, raptures SCVs and promotes LC3 recruitment in an ATG5 dependent manner are not entirely convincing. Several major concerns would need to be addressed before a recommendation concerning publication of the manuscript can be made.

We thank the reviewer for the concise summary of our manuscript: "…a new mechanism by which host cells sense damaged BCVs and induce conjugation of LC3 to activate noncanonical autophagy."

Major:

1) The authors used for their experiments mostly the intracellular pathogens Salmonella and Shigella. The majority of Salmonella remains in SCVs, while Shigella gains rapidly access to the cytoplasm by lysing the BCV. Targeting of TECPR1 to BCVs thus need to be rapid and induction of autophagy must occur very fast, before bacteria can escape. Based on the data presented in this manuscript, it remains unclear whether bacteria already escaped BCVs, are associated with BCV remnants, or are trapped in partially lysed or intact BCVs. This concern also applies to experiments with Salmonella.

In this study we intend to identify bacteria in contact with damaged membranes while bacteria inside intact vacuoles and cytosolic bacteria no longer in contact with damaged membranes are of peripheral interest only. Exposure of sphingomyelin and glycans are excellent markers to identify damaged membranes. The combined detection of sphingomyelin and glycan exposure (as in Ellison, Curr Biol 2020) enables us to determine the degree of membrane damage caused by individual bacteria:
sphingomyelin pos, glycan neg = bacterium in vacuole with minor membrane damage
sphingomyelin pos, glycan pos = bacterium in catastrophically broken vacuole
sphingomyelin neg, glycan neg = bacterium either inside intact vacuole or cytosolic and no longer in contact with damaged membrane

The authors need to establish the integrity of the BCV membrane more carefully by using e.g. electron microscopy.

We have performed correlative light electron tomography on the integrity of the BCV membrane in previous work (Ellison, Curr Biol 2020), which established the combined imaging of sphingomyelin and glycan exposure as an excellent method to identify / distinguish minor and catastrophic membrane damage.

This is critical because the authors claim that the TECPR1 mediated response precedes Gal8 mediated induction of autophagy. The latte mechanism is well-established and known to detect severe damage of BCVs. Along these lines, it is unclear why >30% of Shigella remain associated with BCVs 30 min post infection (Fig 1F), where most of bacteria should have escaped BCVs.

We do not understand why the reviewer thinks that '…bacteria should have (completely) escaped BCVs at 30min post infection'. Entry into the cytosol is neither a perfectly synchronized event nor is it immediate; well-recognized stages of BCV breakage involve membrane stress and catastrophic breakage, indicated by sphingomyelin and glycan exposure, respectively, followed by 'separation' of bacteria from the broken membrane. In our experience cytosolic entry occurs between 15 min and 60 min post-infection and bacteria can remain associated with damaged membranes for

considerable periods afterwards (Fig. EV2B). We therefore think that 30min p.i. is a good time point for the experiment shown in Fig.1F.

If the recruitment of TECPR1 occurs before rupture of the BCVs, why do the author observe colocalization of TECPR1 late (peak at 1 h p.i.)?

Spingomyelin appears on the cytosolic face of stressed membranes and remains present on broken membranes (Ellison, Curr Biol 2020). Therefore, as long as bacteria remain in contact with damaged membranes, they appear TECPR1 positive.

2) The authors found that expression of SMPD3 impaired TECPR1 recruitment to BCVs (Fig 1H). However, the representative images are not very convincing since bacteria were detected by DAPI staining and in most cases, bacteria are in immediate vicinity to the nucleus. In all other experiments, fluorescent labeled bacteria were used. The authors need to repeat these experiments using fluorescent bacteria and combine them with CLEM to establish the morphology of the BCV.

The experiment in Fig.1H requires three channels, in contrast to experiments elsewhere in our manuscript, where we used fluorescently labelled bacteria in simpler two-colour experiments. We prefer to use the green and red channels for TECPR1 and Galectin-8, respectively, thus leaving the blue channel for bacteria. DAPI staining is a well-accepted method to identify bacteria of many different species in the blue channel. We therefore disagree with the reviewer that the experiment needs to be repeated with a different combination of colours.

As requested, we replaced Fig1H with an example where bacteria are not in the vicinity of the nucleus.

3) Along the same lines, the authors reported that the DysF domain of TECPR1 recognizes SM based on it colocalization with BCVs in wt and nSMase2 expressing cells. These data suffer from the same limitation, given that bacteria are again detected by DAPI staining.

As before, Fig.2E is a three-colour experiment, in which detection of bacteria by DAPI is necessary and appropriate.

In corresponding experiments without expression of nSMase2, fluorescent bacteria have been used (Fig. 2G)…

Fig2E and Fig2G are not '…corresponding experiments' as they require three- and two-channel imaging, respectively.

…but it is not clear whether bacteria are trapped in SCVs since the image show single Salmonella, most of which are not associated with DysF (Fig 2G).

We believe the last comment refers to Fig2E rather than Fig2G, as the comment is concerned with the isolated N-terminal DysF domain.

Regarding the reviewer's question whether bacteria shown in Fig2E are '…trapped in SCVs', said bacteria are in close proximity to damaged Gal8+ SCV membranes, a good proxy for sphingomyelin exposure (Fig EV2B), and therefore represent instances on which nSMase is expected to act.

Regarding the criticism of showing only a single bacterium in Fig2E, the image is merely representative for the effect of nSMase expression on TECPR1 recruitment. The corresponding quantification of the nSMase effect in Fig1F is based on several hundred bacteria.

4) The authors validated binding of DysF to SM using liposome floatation assays. The accessibility of exposed surfaces of this domain in context of the full-length protein remains, however, uncertain and TECRP1 full length was not included as control. The authors should repeat these experiments using full length TECPR1 and its corresponding mutant.

We followed the reviewer's advice to further characterize the interactions of the full-length protein with sphingomyelin. New data in Fig.4C-E reveal binding of full length TECPR1 as well as its isolated N-terminal DysF domain to liposomes in a sphingomyelin-dependent manner. To address the effect of point mutations in the full-length protein, we included a conceptually novel experiment designed to test whether TECPR1 can conjugate LC3 to phosphatidylethanoamine in a sphingomyelin-dependent manner. New data in Fig. 4F,G reveal conjugation of LC3 by the TECPR1-ATG5-ATG12

complex specifically to sphingomyelin-containing and not to control liposomes. TECPR1 W154A was inactive. We conclude that TECPR1, in complex with ATG5-ATG12, is a novel *bona fide* E3 ligase catalyzing the conjugation of LC3 to membranes in a sphingomyelin-dependent manner.

Moreover, there is a conceptional problem with these experiments: LUVs are intact, sphingosine will thus be buried in the bilayer core and not be exposed.

LUVs are an excellent and well-established system to investigate the interaction of proteins with biological membranes.

We are unsure why the reviewer refers to 'sphingosine in the bilayer core', as we investigate the interaction of TECPR1 with sphingomyelin, a considerably larger lipid, whose charged headgroup is exposed on the membrane surface and not '…buried in the bilayer core'.

Regarding the reviewer's comment on LUV 'intactness', we would like to emphasize that the internal and external leaflets of LUVs are identical in composition, in contrast to cellular membranes, and that therefore all lipids, including sphingomyelin, are accessible from the outside, even on 'intact' SUVs.

The interaction of DysF with these LUVs might be nonspecific, given that SM are positively charged.
It is thus likely that the interaction of DysF and SM containing liposomes is not driven by hydrophobic interactions of residues of DysF with SM, but by charged interaction. The authors need to increase the salt concentration of the buffer to exclude that charged interactions are responsible.

At physiologically relevant pH sphingomyelin carries one positive and one negative charge. Consistent with the zwitter-ionic nature of sphingomyelin, the binding of the DysF domain to sphingomyelin-containing liposomes does rely on positively charged, negatively charged and hydrophobic residues (for example K152, D143 and W154) (Fig.3B,C), a finding explicitly described in the manuscript (line 253). Evidence for the contribution of charged residues to binding therefore already exists in the manuscript.

The authors should also perform experiments with liposomes containing NBD-PE, which is a probe that changes fluorescence based on the hydrophobicity of its environment to reveal whether lipids are exposed upon incubation with DysF, TECPR1 or corresponding mutants.

The reviewer points out how NBD-PE could provide evidence for the exposure (or not) of lipids from membranes upon TECPR1 binding. We agree that such experiments may yield interesting results. However, we do not propose that binding of TECPR1 or the isolated DysF domain exposes lipids. While interesting in principle, we do not think the proposed experiment is essential for the current manuscript and may rather form part of future work.

5) Given the above-mentioned limitations of the characterization of DysF, the authors should show representative images for their quantification of the recruitment of DysF mutants to Salmonella (Fig 3B and C).

Fig.3B and C report the fraction of bacteria associated with specific DysF domains. The fraction was calculated upon inspection of several hundred bacteria per DysF variant, where each individual bacterium was categorized as 'positive' or 'negative'. To illustrate the methodology, we now include an image with representative examples of DysF positive and DysF negative bacteria inside a single infected cell (Fig EV3D).

Moreover, it remains unclear whether exposed hydrophobic residues in the DysF domain, including W154 and W77, are also exposed in full length TECPR1. This can be evaluated by e.g. HD exchange experiments.

Our revised manuscript provides substantial evidence for the functional importance of W154 in full-length TECPR1. The residue is required for the recruitment of TECPR1 to Salmonella-containing vacuoles (Fig.3E), to LLOMe-damaged lysosomes (new data: Fig.3F) and for the conjugation of LC3 to sphingomyelin-containing liposomes (new data: Fig.4F,G).

Following the reviewer's request, we attempted to analyze full-length TECPR1 by mass spectrometry as a prelude to an HD exchange experiment. However, despite 96% amino acid

coverage, we were unable to detect a peptide covering W154 (Letter Fig. A, W154 highlighted in red box). Note that the negative results were obtained despite using chymotrypsin, an 'optimal' enzyme that was predicted to yield a W154-containing peptide most like to 'fly' in mass spectrometry.

Figures for referees not shown.

6) The result concerning LC3 lipidation/recruitment to Salmonella are puzzling. The authors found that in TECPR1 KO cells, ATG5 is not recruited to bacteria (Fig 4B). However, LC3C is still recruited to Salmonella (Fig. 4D) and the authors concluded that this recruitment occurs in an ATG16L1 dependent manner (Fig 4G).

We agree with the reviewer that LC3 accumulation on BCVs despite lack of ATG5 recruitment in TECPR1 deficient cells is an unexpected finding. We have therefore repeated key experiments in a panel of newly generated isogenic cell lines deficient in TECPR1, ATG16L or ATG5 (Fig 5C,D). Our new results confirm our previous findings: in TECPR1 deficient cells ATG5 is no longer recruited to BCV while LC3 continues to accumulate.

However, if ATG16L1 is not in complex with ATG12-ATG5, it cannot lipidate LC3.

We apologise if we were unclear in our explanation: we do not claim (nor do we expect) that in TECPR1-deficient cells ATG16L1 is no longer bound to ATG5. Rather, we expect that two independent ATG5/ATG12 complexes exist – one containing TECPR1, the other ATG16L1. In our manuscript we merely report that in TECPR1-deficient cells, ATG5 is not detected at damaged vacuoles suggesting that recruitment of ATG5 to damaged BCVs relies on TECPR1, not ATG16L1.

It is thus unclear how ATG16L1 promotes LC3 lipidation if there is no recruitment of ATG5 to Salmonella upon deletion of TECPR1.

We agree with the reviewer that further experimentation is needed to fully understand the accumulation of LC3 on ATG5-negative SCVs in TECPR1 deficient cells. We speculate that LC3 is lipidated by ATG16L1/ATG5/ATG12 at a site away from the damaged SCV and may traffic to the SCV. Further investigation, that we think is beyond the scope of the current manuscript, will be required to fully understand the discrepancy between ATG5 recruitment to and LC3 accumulation on BCVs. Here we merely intend to report the identification of TECPR1 as the first receptor for cytosolically exposed SM and its role as a bona fide E3 ligase for the conjugation of LC3.

To address the reviewer's comment both the Results and Discussion section were amended.

The authors should compare LC3 recruitment to Salmonella and to autophagosomes/phagophores in canonical autophagy (as internal control) to resolve this contradiction.

We agree with the reviewer that further experimentation is needed to fully understand the ATG5/LC3 discrepancy. However, we think that comparing sphingomyelin/TECPR1-dependent targeting of SCVs to canonical autophagy (which presumably is sphingomyelin-independent) is unlikely to resolve the contradiction.

Moreover, previous studies demonstrated that KO of TECPR1 did not affect LC3 lipidation in autophagy, while lipidation was abrogated in ATG16L1 KO cells, arguing that ATG16L1 mediated lipidation dominates.

We completely agree with the reviewer – in the context of recruitment of LC3 to SCVs, ATG16L1-mediated lipidation dominates TECPR1 activity (line 300): "While the majority of LC3 positive SCVs were formed in an ATG16L1-dependent process, about one quarter of LC3 positive SCVs were created in a TECPR1-dependent (and ATG16L1-independent) manner (Figs 4F,G)."

7) The authors showed that in cells overexpressing TECPR1, more Salmonella were Gal8 positive and concluded that TECPR1 induced membrane damage. However, the enhanced recruitment of Gal8 upon TECPR1 overexpression is not a proof of membrane damage by TECPR1. Also, the observation that W154A and delta PH lead to a reduced colocalization of Salmonella and Gal8 is not a proof of membrane damage. Moreover, it is puzzling why TECPR1 recruitment to Salmonella is supposed to induce antibacterial, selective autophagy, given that expression of TECPR1 leads to an enhanced growth of bacteria (Fig 5D). One would expect the opposite effect.

We thank the reviewer for this comment. We have now rewritten this section of the Results. We argue that Galectin-8 is a bona fide marker of damage to SCVs and that its enhanced recruitment reflects more severe membrane damage. Consistent with enhanced damage, we observe increased proliferation of Salmonella.

The reviewer is perceptive in suggesting that an increase in recruitment of TECPR1 would be expected to result in an increase in selective autophagy. Enhanced autophagy may in fact occur but may not suffice to outweigh enhanced cytosolic entry from TECPR1-mediated membrane damage. It is important to notice that CFU assays only report on bacteria that have successfully replicated and therefore do not inform us about the fraction of bacteria that may have been captured, by either TECPR1-dependent or -independent pathways. To experimentally address the reviewers concern it would be necessary to accurately measure the fate of individual Salmonella after recruitment of overexpressed TECPR1 and compare to cells with either endogenous levels or deficient in TECPR1. Such experiments would be extremely time-consuming and have therefore not been attempted.

Minor:

1) Previous studies demonstrated that TECPR1 is localized to lysosomal compartments based on its PH domain. Here, the authors found that TECPR1 is mostly cytoplasmic and puncta colocalized to endosomal compartments. How do the authors explain these differences?

The difference may have to do with fixation as we occasionally observed cells with a more punctate (i.e. potentially partly lysosomal) distribution of TECPR1 in live imaging experiments (Movie S1). However, the majority of TECPR1 always appeared cytosolic and we never obtained any evidence that the recruitment to damaged membranes originates from said puncta. It has been suggested that localisation to lysosomes requires the PH domain, but we demonstrate that recruitment of TECPR1 to damaged SCVs was predominantly via the N terminal DysF domain with only a minor contribution by the PH domain.

2) The authors stated on page 8 line 195 that recruitment of full-length TECPR1 to SCVs peaked at 1 h p.i. (Fig. 2B), However, no time dependent recruitment of TECPR1 is shown in this figure (only shown in the plot in Fig. 2C).

Data in Fig2B and 2C are related. We will call out both figures at once.

Representative images of full-length TECPR1 should be added.

As requested additional images were added to Fig2B.

Referee #2:

Boyle et al., identified that TECPR1 as a cytosolic sensor for sphingomyelin. They showed that TECPR1 bound to sphingomyelin through N-terminal DysF domain. By crystal structural analysis, they also identified key residue for the interaction. Interestingly, TECPR1 function as atypical E3 ligase together with ATG5 and mediated non-canonical lipidation of LC3 independently of ATG16L1. Although the identification of TECRP1 as cytosolic receptor of sphingomyelin and alternative E3 for LC3 lipidation is interesting, the fundamental question why this system is required need to be experimentally solved.

We thank the reviewer for summarizing our findings.

Major

1. Most importantly, authors need to show the biological significance of TECPR1 mediated LC3 lipidation. If loss of TECPR1 does not affect proliferation of S. Typh. compared to ATG16L1, why does this system exist?

Here we report TECPR1 as the first known receptor for cytosolically exposed sphingomyelin and, based on new data added during revision (Fig 4F,G), novel E3 ligase for the conjugation of LC3 to damaged, sphingomyelin-positive membranes.

We identified the N-terminal DysF of TECPR1 as the sphingomyelin-binding domain, solved its crystal structure and verified the importance of TECPR1 in sensing sphingomyelin exposure on damaged membranes. We furthermore demonstrated that upon damage to endomembranes TECPR1 acts as a bona fide E3 ligase mediating a novel and ATG16L1-independent pathway of LC3 conjugation.

Despite out best efforts, we have not been able to demonstrate a biological function for TECPR1 in protecting cells against bacterial infection. However, we expect that our report of a novel E3 ligase mediating sphingomyelin-dependent ligation of LC3 to membranes will spark significant interest in the autophagy community and that future experiments will provide the functional information the reviewer asks for.

2. It seems not enough to propose that TECPR1 mediates LC3 lipidation to damaged endosomes because the results could not dissect canonical and non-canonical lipidation. The authors need to analyze the effect of TECPR1 depletion on LC3 recruitment in canonical autophagy-deficient background such as FIP200 KO.

The reviewer is correct in asserting that we have not formally determined whether TECPR1 acts via a canonical or non-canonical autophagy pathway. To distinguish the contribution of canonical and non-canonical autophagy to the recruitment of LC3 to SCVs we deployed wortmannin, an inhibitor of Class III PI3K, which carries out essential enzymatic function in canonical autophagy only (Figure 5E). We found that wortmannin did not affect recruitment of LC3 in wild type cells, in agreement with previously published findings (Kageyama, Mol Biol Cell 2011). We did, however, observe a defect in LC3 recruitment when wortmannin was added to cells deficient in TECPR1, arguing that Class III PI3K and TECPR1 do not perform epistatic functions but rather contribute to distinct pathways, consistent with a role for TECPR1 in non-canonical autophagy.

In a further attempt to address the reviewer's concern we analysed the effect of ULK inhibition on the recruitment of LC3. We used cells deficient in ATG16L1, where the recruitment of LC3 to SCVs is entirely TECPR1-dependent (Fig 5F,G). We found that inhibition of ULK with MRT68921 did not prevent recruitment of TECPR1-dependent LC3 (Letter Figure B), consistent with a role for TECPR1 in non-canonical autophagy. In fact, MRT68921 even increased LC3 recruitment, suggesting that canonical autophagy may function to inhibit/restrain the TECPR1 pathway. However, exploring the potential inhibition of TECPR1-dependent non-canonical autophagy by ULK requires further work. To avoid distraction from our central findings, we have not included data on ULK inhibition in the manuscript.

Effect of ULK1 inhibition on recruitment of LC3 to Salmonella

Figures for referees not shown.

3. Is there any functional difference between ATG16L1 and TECPR1 dependent LC3 lipidation? Does TECPR1 preferentially mediate lipidation of specific ATG8 subfamilies?

We followed the reviewer's advice to test if lack of TECPR1 selectively affects the conjugation of specific ATG8 family members. No such effect was observed (Letter Figure C).

Figures for referees not shown.

It is also worth to examine the effect on the bacteria growth when TECPR1 is knocked down or overexpressed in ATG16L1 KO.

We followed the reviewer's suggestion. Knockdown of TECPR1 in ATG16L1-deficient cells did not enhance the already strong ATG16L1 phenotype (Letter Figure D).

Figures for referees not shown.

4. Does any other stress such as LLOMe, Monensin, GPN induce TECPR1 recruitment on endo-lysosomal membrane? If so, is DysF domain required?

Following the reviewer's suggestion, we now tested LLOMe, which we found also causes recruitment of TECPR1 but not TECPR1 W154A (Fig 3F).

5. Non-canonical lipidation of LC3 on endo-lysosomal membrane is involved in several biological processes including LAP (Martinez et al., 2015), LANDO (Heckmann et al., 2019), selective lysosome membrane turnover (Lee et al., 2020) and TFEB activation (Goodwin et al., 2021; Nakamura et al., 2020). Is TECPR required for any of these processes?

The reviewer suggests several scenarios in which TECPR1 could play a role. Investigating a possible involvement of TECPR1 comprises a very significant undertaking, which we feel is outside the scope of this revision.

6. When TECPR1(ΔPH) is overexpressed, does TECPR1 accumulate in the damaged membrane? If so, does LC3 also accumulate?

TECPR1 ΔPH is recruited to SCVs similar to the wild type protein (Fig.3F). The majority of TECPR1 recruitment relies on the N-terminal DysF domain as indicated by the severely impaired recruitment of TECPR1 W154A. Simultaneous inactivation of the N-terminal DysF domain and deletion of the PH domain (TECPR1 W154A + ΔPH) completely prevents TECPR1 recruitment. We therefore conclude that the PH domain plays only a minor role in membrane recruitment.

7. Why is LC3 accumulation on SCVs not suppressed in TECPR1 KO cells even though recruitment of ATG5 is completely suppressed? In page 16, line 382 to 384, the authors suggested "ATG16L1-dependent lipidation of LC3 occurring at locations distant from the damaged vacuole. In contrast, it suggests that TECPR1 facilitates LC3 lipidation in the immediate vicinity of the sphingomyelin-positive structure". Which evidence supports this suggestion?

We agree with the reviewer that LC3 accumulation on BCVs despite lack of ATG5 recruitment in TECPR1 deficient cells is an unexpected finding. We have therefore repeated key experiments in an independently generated TECPR1 deficient cell line (Fig 5A-D). Our new results confirm our previous findings: in TECPR1 deficient cells ATG5 is no longer recruited to BCV while LC3 continues to accumulate.

Further experiments are needed to fully understand the accumulation of LC3 on ATG5-negative BCVs in TECPR1 deficient cells. Based on the available data, here we merely hypothesize that ATG5 activity at a membrane distant from the SCV, followed by transport of lipidated LC3 to damaged vacuoles, may explain the phenotype. Alternatively, transient recruitment of ATG5 to the damaged SCV that was undetected in our experiments may cause LC3 conjugation at the vacuole.

8. Is Fig.4E, are comparison between DMSO and Wortmannin significantly different?

   We would like to apologize for the oversight. The difference between DMSO and Wortmannin in Fig4E is statistically significant; the figure has been updated, as have the statistical analysis for other panels throughout.

9. What do the authors intend to suggest from the results in Figure 5?

   Figure 6 (previously Figure 5) reveals that overexpression of TECPR1 causes additional damage to stressed, i.e. sphingomyelin-positive membranes, suggesting that under certain circumstances cells could deploy TECPR1 to perforate endomembranes ad libitum. Importantly, this effect is independent of the AIR domain, thus showing that the effect does not require binding to ATG5-ATG12 and may be possessed by TECPR1 itself. Such need may arise during indirect antigen-presentation, when dendritic cells release extracellular antigen into their cytosol from sphingomyelin-positive vesicles as discussed in lines 490-496.

Minor

1. Line 144, the reference is missing.

   Ellison et al. Current Biology 2020 has been added.

2. Does the deletion N terminal DysF reduce the recruitment of TECPR1 to S. Typh.?

   The N-terminal DysF domain is essential since a point mutation in the domain (TECPR1 W154) severely impairs recruitment (Fig 3F). In addition, we have carried out a comparison of TECPR1 ΔDysF and W154A in their recruitment to SCVs and find that they have a comparable approximately 70% reduction in the recruitment of TECPR (Letter Figure E).

Figures for referees not shown.

3. In Figure 5D, is LC3 recruitment in WT cells comparable to TECPR1 KO cells? Please show the result of WT cell like Figure 5A and 5B.

We have now repeated the experiments in Figure 5A-D in independently derived isogenic cells lines deficient for TECPR1, ATG16L1 or ATG5 and show new panels for each. Data from the isogenic cell lines confirm that TECPR1 is required for the recruitment of ATG5 but not essential for recruitment of LC3. In answer to the reviewer's comment, recruitment of LC3 is comparable between control and TECPR1 KO cells.

4. Even though it is shown that TECPR1 recruitment on BEVs is suppressed by ectopic expression of nSMase2, the authors need to show the correlation of sphingomyelin exposure to cytosol and TECPR1 recruitment on BCVs.

Expression of nSMase2, a known cytosolic sphingomyelin hydrolase, prevents recruitment of the sphingomyelin-specific reporter Lysenin (Ellison, Curr Biol 2020) and, as shown in our current manuscript, markedly reduces recruitment of TECPR1 (with a small sphingomyelin-independent contribution from the PH domain). We thus provide a very strong correlation between sphingomyelin exposure and TECPR1 recruitment.

We provide further evidence for sphingomyelin-dependent binding of TECPR1, based on competition between the N-terminal DysF domain of TECPR1 and Lysenin for binding to the surface of cells, an event known to be dependent on sphingomyelin (Ellison et al., Current Biol 2020) (new data, Fig 4A,B).

5. Please include what the authors found in the last part of introduction.

A summarizing paragraph has been added to the introduction.

Referee #3:

Boyle et al provide a very interesting study which proposes a novel mechanism that cells employ to sense endomembrane stress upon damage, especially during invasive bacterial infection. Authors report that upon membrane damage, sphingomyelin is exposed on the cytosolic leave of the vacuole which is detected by the protein TECPR1, which binds SM and is recruited to the membrane, with authors solving the crystal structure of the domin in TECPR1 requires for SM interaction. TECPR1 then interacts with the autophagy proteins ATG5-12 complex and directs the conjugation of ATG8/LC3 to the membrane. This ATG8 response is part of the non-canonical autophagy/CASM pathway. Importantly, and surprisingly, authors show that part of the ATG8 lipidation is independent of ATG16L1, which has previously been shown to direct the membrane site of lipidation in non-canonical autophagy in conjunction with the v-ATPase. Instead, the TECPR1-ATG5 axis can substitute, which provides a back up mechanism when pathogens evolve strategies to inhibit the ATG16L1-V-ATPase axis, such as SopF.
Authors also provide evidence that TECPR1 recruitment has the potential to exert further damage to membranes, potentially providing a mechanism to turn small membrane damage that is beneficial to the pathogen, to larger damages which can be targeted by the host.
This is a well executed study with many novel findings, including the expansion of the molecular machinery behind non-canonical autophagy. This work highlights previously unappreciated host cell responses. There are some comments that should be addressed.

We are delighted the reviewer considers our manuscript a '… well executed study with many novel findings.'

Major points

1. Fig 4 has some interesting data, in which ATG5 recruitment to SVCs is completely blocked by TECPR1 KO. However, LC3 lipidation to SVCs is not affected, which the authors say is due to the redundancy between the TECPR1 and ATG16L1 pathways. On the face of it this is a confusing result, as both the pathways would require ATG5 for lipidation. Authors offer one explanation, that in the absence of TECPR1, ATG16L1 drives LC3 lipidation at a distant site which then traffics to the SVC. However, there is no evidence supplied to support this. If this was the case, there may be differences in the kinetics of LC3 recruitment to SVCs in WT and TECPR1 KO cells. Authors could examine this by timelapse microscopy.

We agree with the reviewer that LC3 accumulation on BCVs lacking ATG5 recruitment in TECPR1 deficient cells is an unexpected finding. We have therefore repeated key experiments in a panel of independently generated isogenic cell lines deficient in TECPR1, ATG16L1 and ATG5 (Fig 5A-D). Our new results confirmed our previous findings: in TECPR1 deficient cells ATG5 is no longer recruited to SCV while LC3 continues to accumulate.

The reviewer's suggestion of kinetic differences in LC3 recruitment between WT and TECPR1 KO cells is interesting. However, bacterial entry into the cytosol is an asynchronous process and it is unclear how to measure LC3 kinetics without a reliable time point zero.

Further, trafficking or vesicle fusion could be impeded to see if this then blocks the LC3 lipidation in TECPR1 KO cells. Does ATG16L1 KO have any effect on ATG5 recruitment to the SVC?

We followed the reviewer's advice to assess ATG5 recruitment in ATG16L1 KO cells. New data in Fig. 5A,B reveal that deletion of ATG16L1 does not affect recruitment of ATG5.

As it stands, the explanation of this result is not strong; there is no suggestion as to what would regulate/control the ATG16L1 dependent lipidation at distant sites. This at least needs to be discussed more or experimentally investigated.

As suggested by the reviewer, we have expanded the discussion.

2. Fig5 shows data that TECPR1 can lead to increased membrane damage and Gal8 positivity and this is dependent on SM binding. This work was done with over-expresison of TECPR1. Do authors have any evidence that this occurs at physiological levels of expression (or have conditions where baseline levels are increased, does TECPR1 increase during infection)?

We do not have experimental evidence for the upregulation of TECPR1. However, one might imagine a scenario where high local levels of sphingomyelin on damaged membranes would concentrate TECPR1 to exert such an effect.

Can authors see a reduction of Gal8 puncta in TECPR1 KO cells?

Recruitment of Galectin-8 to SCVs is unaffected in TECPR1 KO (Fig EV4B).

3. The strain of Salmonella used in the study would be expected to express SopF, and thus dampen the ATG16L1-V-ATPase axis of non-canonical autophagy, making it more likely that TECPR1 plays a functional role. Can authors comment on or use deltaSopF strains to determine whether when both pathways are active, which plays a more prominent role? Would TECPR1 KO have any effect on ATG5 recruitment under these conditions?

Using WT bacteria, the TECPR1 and the ATG16L1 pathway generate roughly 25% and 75% of LC3 positive SCVs, respectively (Fig.4G). As pointed out by the reviewer, in ∆SopF strains the ATG16L1-V-ATPase pathway is more active). We therefore think that the TECPR1 pathway would affect ∆SopF strains less than wild type bacteria.

Minor point.

1. Many of the graphs are lacking statistical analysis

We apologize for the oversight; statistical analysis has been performed on all graphs.

1st Revision - Editorial Decision 18th May 2023

Dear Felix,

Thank you for submitting your revised manuscript to The EMBO Journal. The three original referees have now looked at it and appreciate the introduced changes. I am therefore very pleased to let you know that we will accept the manuscript for publication in The EMBO Journal. Before sending you the official accept letter there are a few editorial points we need to sort out. When you submit the revised version please respond to following issues:

- we need 3-5 keywords.

- for the Data Availability section if no data is generated that needs to be deposited in a database, then please state: Data Availability: This study includes no data deposited in external repositories.

- COI should be re-labelled as Disclosure and competing interests statement.

- Please remove the Authors Contributions from the manuscript. The 'Author Contributions' section is replaced by the CRediT contributor roles taxonomy to specify the contributions of each author in the journal submission system. Please use the free text box in the 'author information' section of the manuscript submisssion system to provide more detailed descriptions (e.g., 'X provided intracellular Ca++ measurements in fig Y').

- We don't allow data not shown (page 13) please re-phrase.

- Figure callout is missing for Fig. EV3E.

- Please upload individual figure files.

- The movie should be renamed to Movie EV1 with the corresponding callout, and the legend should be zipped with the movie file.

- Table 1 should be embedded in MS file, and Table 2 should be renamed to Table EV1 with the corresponding callout in the text.

- Please correct section order in MS.

- Our publisher has also done their pre-publication check on your manuscript. When you log into the manuscript submission system you will see the file "Data Edited Manuscript file". Please look at the word file and the comments regarding the figure legends and respond to the issues.

- Please upload a synopsis text. We need a summary statement plus 3-5 bullet points describing the key findings of the MS.

- We also need a synopsis image => 550 wide by [200-400]

- We don't encourage showing statistical information when n=2 (Figure 1, Figure 2F, Figure 3B, Figure EV1B, Figure EV2B). Please show both data points

- Our publisher has also done their pre-publication check on your manuscript. When you log into the manuscript submission system you will see the file "Data Edited Manuscript file". Take a look at the word file and the comments regarding the figure legends and respond to the issues.

- Please also upload source data - see attached source data checklist.

- Are panels in Figure 1D reused in Figure 2B if so please indicate in the figure legend.

- Are some of the panels in Figure 2G reused in Figure EV3D?

That should be all. When you submit the revised version please also include a point-by-point response.

Best Karin

Karin Dumstrei, PhD
Senior Editor
The EMBO Journal

Instructions for preparing your revised manuscript:

Guide For Authors: https://www.embopress.org/page/journal/14602075/authorguide

----

Referee #1:

The authors addressed all my comments and I recommend publication of the mansucript in the EMBO Journal.

Referee #2:

The authors responded to all of my concerns I raised for the original manuscript in a satisfactory manner.

Referee #3:

The authors have addressed my major concerns, and while there are still some interesting and puzzling questions raised by the study, these can be addressed in the future.

Point-by-point response

- we need 3-5 keywords.

Done.

- for the Data Availability section if no data is generated that needs to be deposited in a database, then please state: Data Availability: This study includes no data deposited in external repositories.

PDB file for dysferlin domain deposited (PBD identifier 8P5P). Section edited.

- COI should be re-labelled as Disclosure and competing interests statement.

Done.

- Please remove the Authors Contributions from the manuscript. The 'Author Contributions' section is replaced by the CRediT contributor roles taxonomy to specify the contributions of each author in the journal submission system. Please use the free text box in the 'author information' section of the manuscript submisssion system to provide more detailed descriptions (e.g., 'X provided intracellular Ca++ measurements in fig Y').

Done.

- We don't allow data not shown (page 13) please re-phrase.

Done.

- Figure callout is missing for Fig. EV3E.

Added.

- Please upload individual figure files.

Done.

- The movie should be renamed to Movie EV1 with the corresponding callout, and the legend should be zipped with the movie file.

Done.

- Table 1 should be embedded in MS file, and Table 2 should be renamed to Table EV1 with the corresponding callout in the text.

Done.

- Please correct section order in MS.

Done.

- Our publisher has also done their pre-publication check on your manuscript. When you log into the manuscript submission system you will see the file "Data Edited Manuscript file". Please look at the word file and the comments regarding the figure legends and respond to the issues.

Done.

- Please upload a synopsis text. We need a summary statement plus 3-5 bullet points describing the key findings of the MS.

    Done.

- We also need a synopsis image => 550 wide by [200-400]

    Done.

- We don't encourage showing statistical information when n=2 (Figure 1, Figure 2F, Figure 3B, Figure EV1B, Figure EV2B). Please show both data points

    Edited as advised.

- Our publisher has also done their pre-publication check on your manuscript. When you log into the manuscript submission system you will see the file "Data Edited Manuscript file". Take a look at the word file and the comments regarding the figure legends and respond to the issues.

    Done.

- Please also upload source data - see attached source data checklist.

    Done.

- Are panels in Figure 1D reused in Figure 2B if so please indicate in the figure legend.

    We would like to apologize for the mistake. Figure 1D and Figure 2B should not have been identical. Figure 2B was replaced with a new image.

- Are some of the panels in Figure 2G reused in Figure EV3D?

    Yes, Figure EV3D is a replication of Figure 2G. The legend for Figure EV3D has been changed to indicate this fact.

2nd Revision - Editorial Decision **14th Jun 2023**

Dear Felix,

Thank you for submitting your revised manuscript to The EMBO Journal. I have now looked at everything and all looks fine. I am therefore very pleased to accept the manuscript for publication here.

with best wishes

Karin

Karin Dumstrei, PhD
Senior Editor
The EMBO Journal

Please note that it is EMBO Journal policy for the transcript of the editorial process (containing referee reports and your response letter) to be published as an online supplement to each paper. If you do NOT want this, you will need to inform the Editorial Office via email immediately. More information is available here:
https://www.embopress.org/page/journal/14602075/authorguide#transparentprocess

Your manuscript will be processed for publication in the journal by EMBO Press. Manuscripts in the PDF and electronic editions of The EMBO Journal will be copy edited, and you will be provided with page proofs prior to publication. Please note that supplementary information is not included in the proofs.

You will be contacted by Wiley Author Services to complete licensing and payment information. The required 'Page Charges Authorization Form' is available here: https://www.embopress.org/pb-assets/embo-site/tej_apc.pdf - please download and complete the form and return to embopressproduction@wiley.com

EMBO Press participates in many Publish and Read agreements that allow authors to publish Open Access with reduced/no publication charges. Check your eligibility: https://authorservices.wiley.com/author-resources/Journal-Authors/open-access/affiliation-policies-payments/index.html

Should you be planning a Press Release on your article, please get in contact with embojournal@wiley.com as early as possible, in order to coordinate publication and release dates.

If you have any questions, please do not hesitate to call or email the Editorial Office. Thank you for your contribution to The EMBO Journal.

EMBO Press Author Checklist

Corresponding Author Name: Keith Boyle, Felix Randow	
Journal Submitted to: EMBO Journal	
Manuscript Number: #EMBOJ-2022-113012	

USEFUL LINKS FOR COMPLETING THIS FORM
The EMBO Journal - Author Guidelines
EMBO Reports - Author Guidelines
Molecular Systems Biology - Author Guidelines
EMBO Molecular Medicine - Author Guidelines

Reporting Checklist for Life Science Articles (updated January
This checklist is adapted from Materials Design Analysis Reporting (MDAR) Checklist for Authors. MDAR establishes a minimum set of requirements in transparent reporting in the life sciences (see Statement of Task: 10.31222/osf.io/9sm4x). Please follow the journal's guidelines in preparing your
Please note that a copy of this checklist will be published alongside your article.

Abridged guidelines for figures
1. Data
The data shown in figures should satisfy the following conditions:
- the data were obtained and processed according to the field's best practice and are presented to reflect the results of the experiments in an accurate and unbiased manner.
- ideally, figure panels should include only measurements that are directly comparable to each other and obtained with the same assay.
- plots include clearly labeled error bars for independent experiments and sample sizes. Unless justified, error bars should not be shown for technical
- if n<5, the individual data points from each experiment should be plotted. Any statistical test employed should be justified.
- Source Data should be included to report the data underlying figures according to the guidelines set out in the authorship guidelines on Data

2. Captions
Each figure caption should contain the following information, for each panel where they are relevant:
- a specification of the experimental system investigated (eg cell line, species name).
- the assay(s) and method(s) used to carry out the reported observations and measurements.
- an explicit mention of the biological and chemical entity(ies) that are being measured.
- an explicit mention of the biological and chemical entity(ies) that are altered/varied/perturbed in a controlled manner.
- the exact sample size (n) for each experimental group/condition, given as a number, not a range;
- a description of the sample collection allowing the reader to understand whether the samples represent technical or biological replicates (including how many animals, litters, cultures, etc.).
- a statement of how many times the experiment shown was independently replicated in the laboratory.
- definitions of statistical methods and measures:
  - common tests, such as t-test (please specify whether paired vs. unpaired), simple χ2 tests, Wilcoxon and Mann-Whitney tests, can be unambiguously identified by name only, but more complex techniques should be described in the methods section;
  - are tests one-sided or two-sided?
  - are there adjustments for multiple comparisons?
  - exact statistical test results, e.g., P values = x but not P values < x;
  - definition of 'center values' as median or average;
  - definition of error bars as s.d. or s.e.m.

Please complete ALL of the questions below.
Select "Not Applicable" only when the requested information is not relevant for your study.

Materials

Newly Created Materials	Information included in the manuscript?	In which section is the information available? (Reagents and Tools Table, Materials and Methods, Figures, Data Availability Section)
New materials and reagents need to be available; do any restrictions apply?	Yes	Data Availability Section

Antibodies	Information included in the manuscript?	In which section is the information available? (Reagents and Tools Table, Materials and Methods, Figures, Data Availability Section)
For **antibodies** provide the following information:<br>- Commercial antibodies: RRID (if possible) or supplier name, catalogue number and or/clone number<br>- Non-commercial: RRID or citation	Yes	Materials and Methods

DNA and RNA sequences	Information included in the manuscript?	In which section is the information available? (Reagents and Tools Table, Materials and Methods, Figures, Data Availability Section)
**Short novel DNA or RNA including primers, probes**: provide the sequences.	Yes	Table 2

Cell materials	Information included in the manuscript?	In which section is the information available? (Reagents and Tools Table, Materials and Methods, Figures, Data Availability Section)
**Cell lines:** Provide species information, strain. Provide accession number in repository **OR** supplier name, catalog number, clone number, and/**OR** RRID.	Yes	Materials and Methods
**Primary cultures:** Provide species, strain, sex of origin, genetic modification status.	Not Applicable	
Report if the cell lines were recently **authenticated** (e.g., by STR profiling) and tested for mycoplasma contamination.	Yes	Materials and Methods

Experimental animals	Information included in the manuscript?	In which section is the information available? (Reagents and Tools Table, Materials and Methods, Figures, Data Availability Section)
**Laboratory animals or Model organisms:** Provide species, strain, sex, age, genetic modification status. Provide accession number in repository **OR** supplier name, catalog number, clone number, **OR** RRID.	Not Applicable	
**Animal observed in or captured from the field:** Provide species, sex, and age where possible.	Not Applicable	
Please detail **housing and husbandry conditions**.	Not Applicable	

Plants and microbes	Information included in the manuscript?	In which section is the information available? (Reagents and Tools Table, Materials and Methods, Figures, Data Availability Section)
**Plants:** provide species and strain, ecotype and cultivar where relevant, unique accession number if available, and source (including location for collected wild specimens).	Not Applicable	
**Microbes:** provide species and strain, unique accession number if available, and source.	Yes	Materials and Methods

Human research participants	Information included in the manuscript?	In which section is the information available? (Reagents and Tools Table, Materials and Methods, Figures, Data Availability Section)
If collected and within the bounds of privacy constraints report on age, sex and gender or ethnicity for all study participants.	Not Applicable	

Core facilities	Information included in the manuscript?	In which section is the information available? (Reagents and Tools Table, Materials and Methods, Figures, Data Availability Section)
If your work benefited from core facilities, was their service mentioned in the acknowledgments section?	Yes	Acknowledgement

Design

Study protocol	Information included in the manuscript?	In which section is the information available? (Reagents and Tools Table, Materials and Methods, Figures, Data Availability Section)

If study protocol has been **pre-registered, provide DOI in the manuscript**. For clinical trials, provide the trial registration number **OR** cite DOI.	Not Applicable	
Report the **clinical trial registration number** (at ClinicalTrials.gov or equivalent), where applicable.	Not Applicable	

Laboratory protocol	Information included in the manuscript?	In which section is the information available? (Reagents and Tools Table, Materials and Methods, Figures, Data Availability Section)
Provide DOI OR other citation details if **external detailed step-by-step protocols** are available.	Not Applicable	

Experimental study design and statistics	Information included in the manuscript?	In which section is the information available? (Reagents and Tools Table, Materials and Methods, Figures, Data Availability Section)
Include a statement about **sample size** estimate even if no statistical methods were used.	Yes	Materials and Methods. The sample size was based on our experience.
Were any steps taken to minimize the effects of subjective bias when allocating animals/samples to treatment (e.g. **randomization procedure**)? If yes, have they been described?	Not Applicable	
Include a statement about **blinding** even if no blinding was done.	Yes	No blinding was done except for those experiments as described in Figure Legends.
Describe **inclusion/exclusion criteria** if samples or animals were excluded from the analysis. Were the criteria pre-established?<br><br>If sample or data points were omitted from analysis, report if this was due to attrition or intentional exclusion and provide justification.	Yes	No data were excluded.
For every figure, are **statistical tests** justified as appropriate? Do the data meet the assumptions of the tests (e.g., normal distribution)? Describe any methods used to assess it. Is there an estimate of variation within each group of data? Is the variance similar between the groups that are being statistically compared?	Yes	Materials and Methods

Sample definition and in-laboratory replication	Information included in the manuscript?	In which section is the information available? (Reagents and Tools Table, Materials and Methods, Figures, Data Availability Section)
In the figure legends: state number of times the experiment was **replicated** in laboratory.	Yes	Figure Legends
In the figure legends: define whether data describe **technical or biological replicates**.	Yes	Figure Legends

Ethics

Ethics	Information included in the manuscript?	In which section is the information available? (Reagents and Tools Table, Materials and Methods, Figures, Data Availability Section)
Studies involving **human participants**: State details of **authority granting ethics approval** (IRB or equivalent committee(s), provide reference number for approval.	Not Applicable	
Studies involving **human participants**: Include a statement confirming that **informed consent** was obtained from all subjects and that the experiments conformed to the principles set out in the WMA Declaration of Helsinki and the Department of Health and Human Services Belmont Report.	Not Applicable	
Studies involving **human participants:** For publication of **patient photos**, include a statement confirming that consent to publish was obtained.	Not Applicable	
Studies involving experimental **animals**: State details of **authority granting ethics approval** (IRB or equivalent committee(s), provide reference number for approval. Include a statement of compliance with ethical regulations.	Not Applicable	
Studies involving **specimen and field samples:** State if relevant **permits** obtained, provide details of authority approving study; if none were required, explain why.	Not Applicable	

Dual Use Research of Concern (DURC)	Information included in the manuscript?	In which section is the information available? (Reagents and Tools Table, Materials and Methods, Figures, Data Availability Section)
Could your study fall under dual use research restrictions? Please check biosecurity documents and list of **select agents and toxins** (CDC): https://www.selectagents.gov/sat/list.htm	Not Applicable	
If you used a select agent, is the security level of the lab appropriate and reported in the manuscript?	Not Applicable	
If a study is subject to dual use research of concern regulations, is the name of the **authority granting approval and reference number** for the regulatory approval provided in the manuscript?	Not Applicable	

Reporting

The MDAR framework recommends adoption of discipline-specific guidelines, established and endorsed through community initiatives. Journals have their own policy about requiring specific guidelines and recommendations to complement MDAR.

Adherence to community standards	Information included in the manuscript?	In which section is the information available? (Reagents and Tools Table, Materials and Methods, Figures, Data Availability Section)
State if relevant guidelines or checklists (e.g., **ICMJE, MIBBI, ARRIVE, PRISMA**) have been followed or provided.	Not Applicable	
For **tumor marker prognostic studies,** we recommend that you follow the **REMARK** reporting guidelines (see link list at top right). See author guidelines, under 'Reporting Guidelines'. Please confirm you have followed these guidelines.	Not Applicable	
For **phase II and III randomized controlled trials**, please refer to the **CONSORT** flow diagram (see link list at top right) and submit the CONSORT checklist (see link list at top right) with your submission. See author guidelines, under 'Reporting Guidelines'. Please confirm you have submitted this list.	Not Applicable	

Data Availability

Data availability	Information included in the manuscript?	In which section is the information available? (Reagents and Tools Table, Materials and Methods, Figures, Data Availability Section)
Have **primary datasets** been deposited according to the journal's guidelines (see 'Data Deposition' section) and the respective accession numbers provided in the Data Availability Section?	Yes	Data Availability Section
Were **human clinical and genomic datasets** deposited in a public access-controlled repository in accordance to ethical obligations to the patients and to the applicable consent agreement?	Not Applicable	
Are **computational models** that are central and integral to a study available without restrictions in a machine-readable form? Were the relevant accession numbers or links provided?	Not Applicable	
If publicly available data were reused, provide the respective **data citations in the reference list**.	Not Applicable	
